# Costs of Endoscopic vs Open Vein Harvesting for Coronary Artery Bypass Grafting: A Secondary Analysis of the REGROUP Trial

**DOI:** 10.1001/jamanetworkopen.2022.17686

**Published:** 2022-06-21

**Authors:** Todd H. Wagner, Brack Hattler, Eileen M. Stock, Kousick Biswas, Deepak L. Bhatt, Faisal G. Bakaeen, Kritee Gujral, Marco A. Zenati

**Affiliations:** 1 Health Economics Resource Center, Veterans Affairs Palo Alto Health Care System, Menlo Park, California; 2 Department of Surgery, Stanford University, Stanford, California; 3 Rocky Mountain Regional Veterans Affairs Medical Center, Aurora, Colorado; 4 Division of Cardiology, University of Colorado, Denver; 5 Office of Research and Development, VA Cooperative Studies Program Coordinating Center, Perry Point, Maryland; 6 Division of Cardiovascular Medicine, Brigham and Women’s Hospital and Harvard Medical School, Boston, Massachusetts; 7 Department of Thoracic and Cardiovascular Surgery, Cleveland Clinic, Cleveland, Ohio; 8 Division of Cardiac Surgery, Brigham and Women’s Hospital and Harvard Medical School, Boston, Massachusetts; 9 Veterans Affairs Boston Healthcare System, Boston, Massachusetts

## Abstract

**Importance:**

Value-based purchasing creates pressure to examine whether newer technologies and care processes, including new surgical techniques, yield any economic advantage.

**Objective:**

To compare health care costs and utilization between participants randomized to receive endoscopic vein harvesting (EVH) or open vein harvesting (OVH) during a coronary artery bypass grafting (CABG) procedure.

**Design, Setting, and Participants:**

This secondary economic analysis was conducted alongside the 16-site Randomized Endo-Vein Graft Prospective (REGROUP) clinical trial funded by the Department of Veterans Affairs (VA) Cooperative Studies Program. Adults scheduled for urgent or elective bypass involving a vein graft were eligible. The first participant was enrolled in September 2013, with most sites completing enrollment by March 2014. The last participant was enrolled in April 2017. A total of 1150 participants were randomized, with 574 participants receiving OVH and 576 receiving EVH. For this secondary analysis, cost and utilization data were extracted through September 30, 2020. Participants were linked to administrative data in the VA Corporate Data Warehouse and activity-based cost data starting with the index procedure.

**Interventions:**

EVH vs OVH, with comparisons based on intention to treat.

**Main Outcomes and Measures:**

Discharge costs for the index procedure as well as follow-up costs (including intended and unintended events; mean [SD] follow-up time, 33.0 [19.9] months) were analyzed, with results from different statistical models compared to test for robustness (ie, lack of variation across models). All costs represented care provided or paid by the VA, standardized to 2020 US dollars.

**Results:**

Among 1150 participants, the mean (SD) age was 66.4 (6.9) years; most participants (1144 [99.5%] were male. With regard to race and ethnicity, 6 participants (0.5%) self-reported as American Indian or Alaska Native, 10 (0.9%) as Asian or Pacific Islander, 91 (7.9%) as Black, 62 (5.4%) as Hispanic, 974 (84.7%) as non-Hispanic White, and 6 (0.5%) as other race and/or ethnicity; data were missing for 1 participant (0.1%). The unadjusted mean (SD) costs for the index CABG procedure were $76 607 ($43 883) among patients who received EVH and $75 368 ($45 900) among those who received OVH, including facility costs, insurance costs, and physician-related costs (commonly referred to as provider costs in Centers for Medicare and Medicaid and insurance data). No significant differences were found in follow-up costs; per 90-day follow-up period, EVH was associated with a mean (SE) added cost of $302 ($225) per patient. The results were highly robust to the statistical model.

**Conclusions and Relevance:**

In this study, EVH was not associated with a reduction in costs for the index CABG procedure or follow-up care. Therefore, the choice to provide EVH may be based on surgeon and patient preferences.

**Trial Registration:**

ClinicalTrials.gov Identifier: NCT01850082

## Key Points

**Question:**

Is there any difference in health care costs for patients who undergo endoscopic vein harvesting rather than open vein harvesting during a coronary artery bypass surgical procedure?

**Findings:**

In this secondary analysis of cost outcomes from a randomized clinical trial involving 1150 participants undergoing a coronary artery bypass procedure, the use of endoscopic vein harvesting was not associated with lower costs at discharge or over time compared with open vein harvesting. None of the cost end points were significantly different.

**Meaning:**

This study’s findings suggest that the choice to use endoscopic vein harvesting does not increase economic burden and may be based on surgeon and patient preferences.

## Teaser

This secondary analysis of cost outcomes from the Randomized Endo-Vein Graft Prospective (REGROUP) clinical trial compared health care costs between participants randomized to receive endoscopic vein harvesting vs open vein harvesting during a coronary artery bypass grafting procedure.

## Introduction

Coronary artery bypass grafting (CABG) remains a treatment option for patients with ischemic heart disease. During the procedure, saphenous veins are frequently used for grafts. Harvesting the saphenous vein was traditionally performed using open vein harvesting (OVH), but endoscopic vein harvesting (EVH) has become the dominant approach since its introduction in the 1990s. The Randomized Endo-Vein Graft Prospective (REGROUP) clinical trial^1^ found that the harvesting technique did not change the performance characteristics of the vein graft with regard to cardiac end points. Both EVH and OVH had comparable rates of major adverse cardiac events (death, revascularization, or myocardial infarction) over a median follow-up of 2.78 years.^1^ Overall, leg infections were rare, but leg infections were approximately twice as common among participants receiving OVH vs EVH (3.1% vs 1.4%; relative risk, 2.26; 95% CI, 0.99-5.15), although this difference was not statistically significant.

Value-based purchasing has encouraged more analyses of the relative costs of newer technologies, approaches, and techniques. There is a desire to examine new surgical approaches, such as EVH, to assess whether they yield any intended or unintended economic consequences. The objective of this secondary analysis of cost outcomes from the REGROUP clinical trial was to compare health care costs between participants randomized to receive EVH or OVH during a coronary artery bypass grafting (CABG) procedure. We hypothesized that EVH would be associated with lower discharge and follow-up costs (including intended and unintended events) compared with traditional OVH.

## Methods

### Data

**Figure 1.**
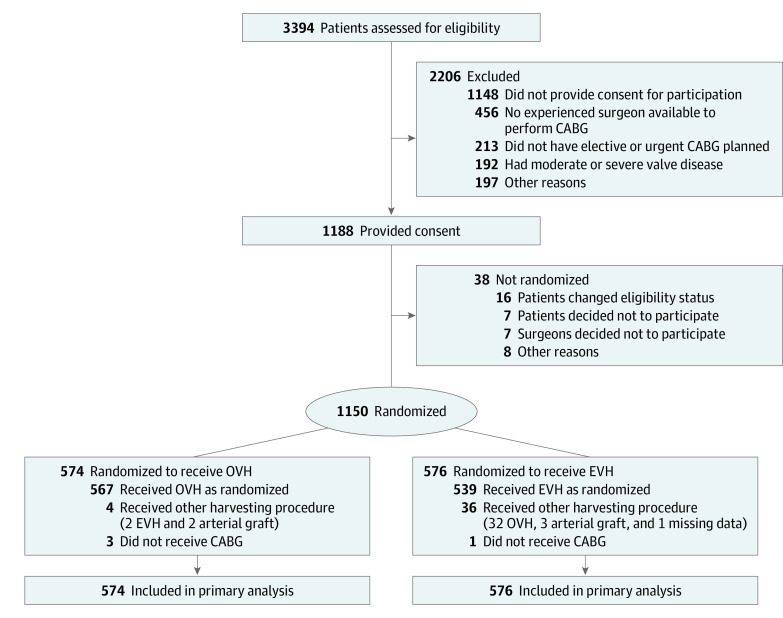
Participant Flowchart CABG indicates coronary artery bypass graft; EVH, endoscopic vein harvesting; and OVH, open vein harvesting.

The REGROUP clinical trial, funded by the Department of Veterans Affairs (VA) Cooperative Studies Program, randomized 1150 participants (574 received OVH and 576 received EVH) at 16 VA medical centers. Adults scheduled for urgent or elective bypass involving a vein graft were eligible for participation. All patients provided written informed consent to participate in the clinical trial. The REGROUP clinical trial followed the Consolidated Standards of Reporting Trials (CONSORT) reporting guideline for randomized clinical trials (Figure 1). The VA Palo Alto Research and Development Committee and the Stanford University Institution Review Board approved the study protocol, with a waiver of informed consent to use VA administrative data for the current secondary analysis.

The REGROUP clinical trial was designed to assess whether OVH was superior to EVH based on a primary outcome of time to first major adverse cardiac event (a composite of all-cause death, myocardial infarction, and repeat revascularization).^1^ As described elsewhere,^1^ the power analysis was based on a composite end point of 16.5% for EVH and 8.5% for OVH, leading to a target of 1150 participants. The first participant was enrolled in September 2013, with most sites completing enrollment by March 2014. The last participant was enrolled in April 2017. For this secondary economic analysis, we extracted cost and utilization data through September 30, 2020. The mean (SD) follow-up was 33.0 (19.9) months, with a maximum follow-up of 85 months.

Economic end points were not specified in the original clinical trial protocol (Supplement 1). With supplemental funding from the VA Cooperative Studies Program, we linked participants to administrative data in the VA Corporate Data Warehouse^2^ starting with the index procedure. The analysis used a VA perspective. The cost data for VA-provided care came from the VA Managerial Cost Accounting System,^3^ which includes cost estimates for all VA care, including inpatient, outpatient, and pharmacy. The Managerial Cost Accounting System uses activity-based cost methods, in which the care provided to each patient is linked with unit costs. The system tracks care as measured by detailed inputs, including time in the operating room, staff time, supplies, medications, laboratory tests, and overhead. This costing method is considered among the best for economic evaluations because it reflects the resources used to provide care, and it is highly precise.^4^ These data have been extensively used in research, including in analyses examining the costs of other surgical approaches to CABG.^5,6^ All costs were standardized to 2020 US dollars. The VA administrative data were linked to clinical and patient data collected during the clinical trial. For VA-purchased care, we used data from the Fee Basis Claims System^7^ and the Program Integrity Tool.^8^

### Outcomes

Two separate cost outcomes were assessed. First, we analyzed discharge costs for the index procedure. This outcome included information costs in the surgical suite and any added costs for differences in length of stay or critical care. In previous work,^5,6,9^ separating the costs of the index stay from subsequent care provided greater insights into the costs of the index stay itself, and this approach avoided the statistical challenges of combining a high-cost admission with follow-up costs that include zeros. We graphed the distribution of discharge costs using kernel densities.^10^

Second, we analyzed downstream costs for follow-up care after the index stay. We combined VA costs into 90-day follow-up periods up to the last follow-up at 85 months (mean [SD], 33.0 [19.9] months). The first 90-day period excluded the cost of the index discharge. The 90-day periods were preferred over shorter 30-day periods, which exhibited considerably more unexplained variability (greater SEs) resulting from 3.3 times more patient observations with zero costs in the shorter period. If patients had an inpatient stay that spanned two 90-day periods, the costs were assigned to each period proportionately based on the length of stay. In the follow-up periods, we examined total VA costs along with subtotals for inpatient care and, more specifically, inpatient medical-surgical care. We also estimated total outpatient costs, outpatient medical-surgical costs, and outpatient pharmacy costs. Subtotals were based on the inpatient treating specialties and outpatient clinic.^11,12^ We then examined VA-purchased care, which has expanded since the passage of the Veterans Access, Choice, and Accountability Act (commonly referred to as the Choice Act)^13,14^ and the VA Maintaining Internal Systems and Strengthening Integrated Outside Networks (MISSION) Act.^13,15^ For follow-up care, we also examined VA health care utilization; this measure can sometimes provide insights into mechanisms that may underlie any cost differences.

### Statistical Analysis

We followed intention-to-treat principles throughout the analysis. We examined the characteristics of patients who received EVH and OVH. Next, we compared unadjusted mean costs by randomized treatment group. For the regression analysis, the protocol did not specify a preferred statistical model, so we used a Box-Cox transformation,^16^ a modified Park test,^17,18^ and empirical goodness-of-fit tests to guide our choice. We then compared results from different statistical models to test for robustness (ie, lack of variation across models). For the analysis of discharge costs, the Box-Cox model suggested a log transformation, the modified Park test was inconclusive, and the Hosmer-Lemeshow statistics for model fit favored an ordinary least squares approach. Thus, we chose ordinary least squares as the primary model and used a generalized linear model with a log link and gamma error distribution in the sensitivity analysis. For the follow-up costs, because the fit statistics did not favor a specific model, we used a linear model with a person random effect to account for the nonindependence of the error terms. We used generalized estimating equations with a log link, gamma error distribution, and exchangeable correlation matrix in the sensitivity analysis. For the utilization outcomes, which included inpatient stays, unique days with an outpatient visit, unique days with an outpatient visit to a medical or surgical clinic, and pharmacy prescriptions, we used a Poisson model with a person random effect. The significance threshold was *P* = .05, and 2-tailed tests were used for all analyses. Stata software, version 16.1 (StataCorp LLC), was used for analysis.

The Managerial Cost Accounting System data are computed using local staff time and wages. To remove variation due to wage differences across the 16 sites, we included a site fixed effect in the regression models. We controlled for the presence of diabetes, hypertension, hyperlipidemia, depression, chronic kidney disease, and chronic liver disease at baseline. These conditions have been associated with a higher prevalence of adverse events, higher expected costs, and worse patient outcomes, and any imbalance in randomization could have confounded the results. We also controlled for patient age, sex (male or female), and self-reported race and ethnicity (American Indian or Alaska Native, Asian or Pacific Islander, Black, Hispanic, non-Hispanic White, and other race and/or ethnicity), as collected by study coordinators on the clinical trial case report forms. We collapsed race and ethnicity categories into non-Hispanic White vs all other racial and ethnic groups given the small samples and the results of analyses revealing no differences in total costs by each racial and/or ethnic group. Costs may be associated with mortality and imbalanced survival, especially when death is common, which can add challenges to interpreting a cost analysis.^19^ As reported previously,^1^ the mortality rate during the active follow-up period did not exceed 10% and was not statistically different across the groups, so we made no adjustments for mortality.

The clinical trial protocol did not prespecify any subgroup analyses. We explored 6 subgroups: diabetes, hypertension, hyperlipidemia, depression, chronic kidney disease, and chronic liver disease. For these exploratory post hoc analyses, we used a threshold of *P* = .01 as a penalty for multiple comparisons.

## Results

### Participant Characteristics

Participant characteristics are shown in Table 1. Further details on the participants and the surgical procedures have been previously published.^1^ Among 1150 participants, the mean (SD) age was 66.4 (6.9) years; 1144 participants (99.5%) were male, and 6 (0.5%) were female. With regard to race and ethnicity, 6 participants (0.5%) self-reported as American Indian or Alaska Native, 10 (0.9%) as Asian or Pacific Islander, 91 (7.9%) as Black, 62 (5.4%) as Hispanic, 974 (84.7%) as non-Hispanic White, and 6 (0.5%) as other race and/or ethnicity; data were missing for 1 participant (0.1%). At randomization, 1036 participants (90.1%) had hypertension, 993 (86.3%) had hyperlipidemia, and 577 (50.2%) had diabetes. Depression (297 participants [25.8%]), chronic kidney disease (157 participants [13.7%]), and chronic liver disease (42 participants [3.7%]) were less common. There were no significant demographic or clinical differences between patients who received EVH vs OVH.

**Table 1.** Participant Characteristics

Characteristic	Participants, No. (%)	
	Open vein harvesting	Endoscopic vein harvesting
Total participants, No.	574	576
Age, mean (SD), y	66.6 (7.1)	66.2 (6.7)
Sex		
Male	571 (99.5)	573 (99.5)
Female	3 (0.5)	3 (0.5)
Race and ethnicity^a^		
Non-Hispanic White	484 (84.3)	490 (85.1)
Other race and/or ethnicity	90 (15.7)	86 (14.9)
Diabetes	297 (51.7)	280 (48.6)
Hypertension	515 (89.7)	521 (90.5)
Hyperlipidemia	502 (87.5)	491 (85.2)
Depression	147 (25.6)	150 (26.0)
Chronic kidney disease	74 (12.9)	83 (14.4)
Chronic liver disease	22 (3.8)	20 (3.5)

^a^ Variables are specified as they were included in the statistical regression models. For these models, race and ethnicity categories were collapsed into non-Hispanic White vs other race and/or ethnicity (including American Indian or Alaska Native, Asian or Pacific Islander, Black, Hispanic, other, and missing).

### Discharge Costs

**Figure 2.**
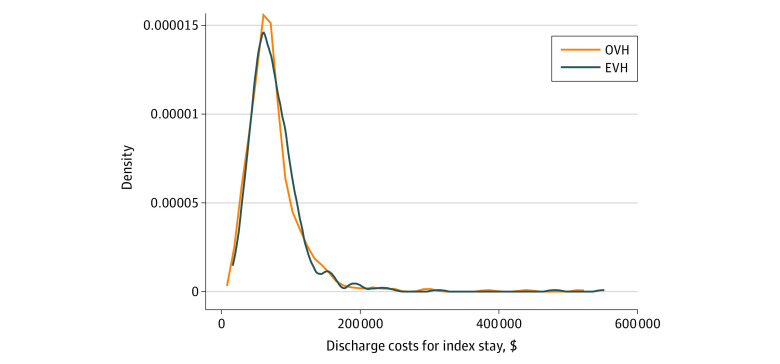
Distribution of Unadjusted Costs of Index Admission for Coronary Artery Bypass Graft Distribution was measured using kernel density. Mean (SD) costs were $75 368 ($45 900) for endoscopic vein harvesting (EVH) and $76 607 ($43 883) for open vein harvesting (OVH).

The unadjusted mean (SD) discharge costs for CABG were $76 607 ($43 883) per patient among those in the EVH group and $75 368 ($45 900) per patient among those in the OVH group, including facility costs, insurance costs, and physician-related costs (commonly referred to as provider costs in Centers for Medicare and Medicaid and insurance data). Follow-up costs were highest in the year after the index procedure; mean follow-up costs then decreased to approximately $5000 per quarter, or $20 000 per year (Figure 2). Despite the skewed variation in costs, there were no significant differences in discharge costs, and high costs among patients were equally likely in both groups. The regression models yielded a similar result; EVH was $1253 (95% CI, −$3564 to $6070) more expensive than OVH, but this difference was not statistically significant. The results were highly robust to the statistical model. In the generalized linear model, OVH was 2.8% ($2158) more expensive than EVH, but this difference was also not significant. There were no differences in discharge costs among any of the clinical subgroups (eTable 1 in the Supplement).

### Follow-up Costs and Utilization

**Figure 3.**
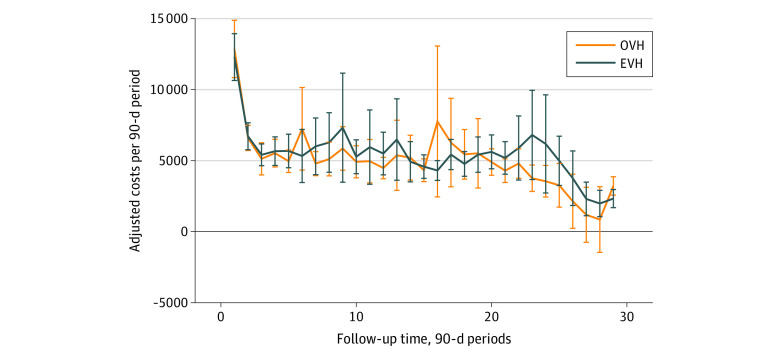
Mean Adjusted Follow-up Costs per 90-Day Period The mean (SD) follow-up was 33.0 (19.9) months. Each follow-up period was 90 days from the date of the index procedure. Ten follow-up periods represent approximately 2.5 years; 20 follow-up periods, approximately 5 years; and 30 follow-up periods, approximately 7.5 years. Whiskers represent 95% CIs. EVH indicates endoscopic vein harvesting; and OVH, open vein harvesting.

We found no significant differences in follow-up costs over a mean (SD) of 33.0 (19.9) months. The marginal estimate and 95% CIs are shown in Table 2. Per 90-day follow-up period, the total added mean (SE) cost for EVH was $302 ($225) per patient ($4351 [95% CI, $4023-$4679] vs $4049 [95% CI, $3757-$4341] for OVH), a nonsignificant difference. Compared with OVH, the use of EVH added costs of $1238 per patient in the unadjusted analysis and $1439 per patient in the adjusted analysis (Figure 3); however, these differences were not statistically significant. The sensitivity analyses using generalized estimating equation models revealed the results were highly robust to the statistical model (eTable 2 in the Supplement). The sample size decreased with follow-up time (eg, 1149 participants in the first 90-day period vs 2 participants in the last 90-day period), leading to less power to detect differences in the longer-term follow-up (eTable 3 in the Supplement includes the sample sizes for follow-up periods).

**Table 2.** Added Costs for Endoscopic Vein Harvesting Over Time

Analysis	Adjusted estimate (95% CI)		Difference
	Open vein harvesting	Endoscopic vein harvesting	
**Costs, $^a^**			
VA-provided care			
Total	4049 (3757-4341)	4351 (4023-4679)	302
Inpatient			
Total	438 (376-501)	480 (414-546)	42
Medical-surgical	395 (334-455)	434 (370-498)	40
Outpatient			
Total	3612 (3334-3891)	3863 (3548-4178)	250
Medical-surgical	2018.15 (1888-2149)	2074 (1933-2215)	56
Prescriptions	649 (523-775)	655 (551-759)	6
VA-purchased care	1608 (1099-2117)	1569 (1071-2067)	−39
**VA utilization^b^**			
Inpatient admissions, No.	1.8 (1.8-1.9)	1.8 (1.8-1.9)	0
Days with an outpatient visit, No.			
Total^a^	11.0 (10.5-11.4)	11.2 (10.7-11.7)	0.3
Medical-surgical^b^	3.8 (3.6-4.0)	3.9 (3.7-4.1)	0.1
Prescriptions, No.	6.4 (6.1-6.7)	6.5 (6.2-6.8)	0.2

Abbreviation: VA, Department of Veterans Affairs.

^a^ Costs are per person. The cost analysis used a linear model with a person random effect, controlling for follow-up period, age, sex, race and ethnicity, diabetes, hypertension, hyperlipidemia, depression, chronic kidney disease, chronic liver disease, and VA hospital site.

^b^ The utilization analysis used a Poisson model with a person random effect, controlling for follow-up period, age, sex, race and ethnicity, diabetes, hypertension, hyperlipidemia, depression, chronic kidney disease, and chronic liver disease.

Additional VA resource utilization among patients who received EVH is shown in Table 2. There were no differences in the number of inpatient VA admissions (mean, 1.8 [95% CI, 1.8-1.9] for both groups), total number of unique days with an outpatient visit (mean, 11.2 [95% CI, 10.7-11.7] for EVH vs 11.0 [95% CI, 10.5-11.4] for OVH; difference, 0.3 days), number of unique days with an outpatient medical-surgical visit (mean, 3.9 [95% CI, 3.7-4.1] for EVH vs 3.8 [95% CI, 3.6-4.0] for OVH; difference, 0.1 days), or number of outpatient prescriptions (mean, 6.5 [95% CI, 6.2-6.8] for EVH vs 6.4 [95% CI, 6.1-6.7] for OVH; difference, 0.2 prescriptions). No differences were found among any of the clinical subgroups (eTable 1 in the Supplement).

## Discussion

Contrary to our hypothesis, this secondary analysis of cost outcomes from the REGROUP clinical trial found no evidence that EVH was associated with lower discharge costs compared with OVH. In the index hospitalization, the unadjusted mean cost of CABG with OVH was $75 368, which included hospital as well as physician and surgeon costs. Use of EVH added $1238 and $1439 in costs relative to OVH in the unadjusted and adjusted analyses, respectively, but neither difference was statistically significant. As noted in the introduction, there was a higher number of leg infections in the OVH vs EVH group,^1^ but these infections were not associated with significantly higher costs for the index stay. Although the distribution of discharge costs was skewed (Figure 1), OVH and EVH had largely overlapping distributions, and nonsignificant differences were also found in generalized estimating equation models that addressed the skewness.

Also contrary to our hypothesis, follow-up costs were not statistically different for EVH and OVH. Both the cost and utilization results were consistent, and the cost analyses were precise. Although it is often difficult to make definitive statements about a null effect, the conclusions are likely valid for 2 reasons. First, the data were obtained from a large multisite clinical trial in which patients were randomized within site to receive either OVH or EVH, removing any chance of selection bias from either the patient or physician. Second, the analysis had complete follow-up data for veterans who used the VA and used cost data from an activity-based cost accounting system. These cost data provided considerably more precision and accuracy than alternative data, such as payments or charges,^4,20^ and they have been used for analysis in other clinical trials of CABG.^5,6^

Follow-up costs were highest in the year after the index procedure (Figure 2); mean follow-up costs then plateaued to approximately $5000 per quarter, or $20 000 per year (in 2020 dollars). A recent study^21^ that followed up VA patients who were randomized to receive on-pump or off-pump bypass reported similar mean costs over 10 years. In that analysis, mean costs plateaued at approximately $20 000 through year 7, with a slight increase in mean costs between 7 and 10 years.^21^

In 2015, Oddershede et al^22^ published results from a cost-effectiveness analysis using a Markov model simulation. Their model synthesized the best evidence at the time and concluded that EVH cost an additional £8219 per quality-adjusted life-year based on 2014 values (equivalent to approximately $13 740 in 2020). The use of EVH was cost-effective at a threshold of £30 000 per quality-adjusted life-year (equivalent to approximately $38 511 in 2020) in 60.4% of their simulations, but the authors noted uncertainty in these estimates.^22^ A major factor in this uncertainty was the small difference in expected event rates. The results of our study, in combination with the clinical end points reported elsewhere,^1,23^ highlight the lack of any significant differences between EVH and OVH. In other words, neither approach was superior to the other based on clinical or economic criteria. Thus, the choice to use EVH or OVH may be best guided by surgeon and patient preferences.

### Limitations

This study has several limitations. First, we included information on VA-provided and VA-purchased care. However, we did not have cost data from other insurers. Previous analyses^5,9^ found that veterans who participate in a surgical clinical trial typically have high reliance on the VA; although it is feasible to obtain Medicare fee-for-service claims, an increasing proportion of Medicare beneficiaries enroll in Medicare Advantage, for which we do not have data. We also do not have access to private insurance claims for veterans not eligible for Medicare. Second, this study lacked patient outcome data. Although costs are part of the value equation, we would ideally have had information on patient outcomes, such as quality of life, beyond our survival data. The survival and major cardiac adverse event end points have been published elsewhere and revealed no significant differences between OVH and EVH.^1,23^ Although our study had a mean follow-up period of 33 months, most participants were still alive at follow-up and were thus censored. Future analyses, based on a pending proposal, will examine longer-term follow-up. Third, the study was conducted within the VA and included mostly men. The results may not be generalizable to women.

## Conclusions

In this secondary analysis of cost outcomes from the REGROUP clinical trial, the use of EVH was not associated with a reduction in costs for the index CABG procedure or follow-up care. These results suggest that the choice to use EVH vs OVH may be based on surgeon and patient preferences.

## Notes

Supplement 1.
Trial Protocol and Statistical Analysis Plan

Supplement 2. **eTable 1.** Subgroup Analyses for 6 Chronic Conditions **eTable 2.** Adjusted Follow-up Costs From GEE Models With a Log Link and a Gamma Error Distribution **eTable 3.** Number of Participants Followed Up by 90-Day Follow-up Period

Supplement 3.
Data Sharing Statement

## References

[1] Zenati MA, Bhatt DL, Bakaeen FG, ; REGROUP Trial Investigators. Randomized trial of endoscopic or open vein–graft harvesting for coronary-artery bypass. N Engl J Med. 2019;380(2):132-141. doi:10.1056/NEJMoa1812390 30417737

[2] Veterans Health Administration. Corporate Data Warehouse (CDW). US Department of Veterans Affairs. Updated December 1, 2020. Accessed May 6, 2022. https://www.data.va.gov/dataset/Corporate-Data-Warehouse-CDW-/ftpi-epf7

[3] Veterans Health Administration. Managerial Cost Accounting (MCA). US Department of Veterans Affairs. Updated February 16, 2022. Accessed May 6, 2022. https://www.herc.research.va.gov/include/page.asp?id=managerial-cost-accounting

[4] Barnett PG. An improved set of standards for finding cost for cost-effectiveness analysis. Med Care. 2009;47(7)(suppl 1):S82-S88. doi:10.1097/MLR.0b013e31819e1f3f 19536018

[5] Wagner TH, Sethi G, Holman W, . Costs and quality of life associated with radial artery and saphenous vein cardiac bypass surgery: results from a Veterans Affairs multisite trial. Am J Surg. 2011;202(5):532-535. doi:10.1016/j.amjsurg.2011.06.011 21872209

[6] Wagner TH, Hattler B, Bishawi M, ; VA #517 Randomized On/Off Bypass (ROOBY) Study Group. On-pump versus off-pump coronary artery bypass surgery: cost-effectiveness analysis alongside a multisite trial. Ann Thorac Surg. 2013;96(3):770-777. doi:10.1016/j.athoracsur.2013.04.074 23916805

[7] Veterans Health Administration. DSS Fee Basis Claims System (FBCS). US Department of Veterans Affairs. April 20, 2020. Accessed May 6, 2022. https://www.oit.va.gov/Services/TRM/ToolPage.aspx?tid=8604

[8] Veterans Health Administration. Community care data—Program Integrity Tool. US Department of Veterans Affairs. Updated April 18, 2022. Accessed May 6, 2022. https://www.herc.research.va.gov/include/page.asp?id=choice-pit

[9] Wagner TH, Hattler B, Bakaeen FG, ; VA #517 Randomized On/Off Bypass (ROOBY) Study Group. Costs five years after off-pump or on-pump coronary artery bypass surgery. Ann Thorac Surg. 2019;107(1):99-105. doi:10.1016/j.athoracsur.2018.07.075 30273569

[10] Tarter ME, Lock MD. Model-Free Curve Estimation. Chapman & Hall; 1993.

[11] Wagner TH, Chen S, Barnett PG. Using average cost methods to estimate encounter-level costs for medical-surgical stays in the VA. Med Care Res Rev. 2003;60(3)(suppl):15S-36S. doi:10.1177/1077558703256485 15095543

[12] Phibbs CS, Bhandari A, Yu W, Barnett PG. Estimating the costs of VA ambulatory care. Med Care Res Rev. 2003;60(3)(suppl):54S-73S. doi:10.1177/1077558703256725 15095546

[13] Rose L, Aouad M, Graham L, Schoemaker L, Wagner T. Association of expanded health care networks with utilization among Veterans Affairs enrollees. JAMA Netw Open. 2021;4(10):e2131141. doi:10.1001/jamanetworkopen.2021.31141 34698845 PMC8548943

[14] Veterans Access, Choice, and Accountability Act of 2014, HR 3230, 113th Congress (2013-2014). Accessed May 6, 2022. https://www.congress.gov/bill/113th-congress/house-bill/3230

[15] VA MISSION Act of 2018, S 2372, 115th Congress (2017-2018). Accessed May 6, 2022. https://www.congress.gov/bill/115th-congress/senate-bill/2372/text

[16] Sakia RM. The Box-Cox transformation technique: a review. *J R Stat Soc D Stat*. 1992;41(2):169-178. doi:10.2307/2348250

[17] Mullahy J. Much ado about two: reconsidering retransformation and the two-part model in health econometrics. J Health Econ. 1998;17(3):247-281. doi:10.1016/S0167-6296(98)00030-7 10180918

[18] Manning WG, Mullahy J. Estimating log models: to transform or not to transform? J Health Econ. 2001;20(4):461-494. doi:10.1016/S0167-6296(01)00086-8 11469231

[19] Lin DY. Linear regression analysis of censored medical costs. Biostatistics. 2000;1(1):35-47. doi:10.1093/biostatistics/1.1.35 12933524

[20] Shwartz M, Young DW, Siegrist R. The ratio of costs to charges: how good a basis for estimating costs? Inquiry. 1995-1996;32(4):476-481. 8567084

[21] Quin JA, Wagner TH, Hattler B, . Ten-year outcomes of off-pump vs on-pump coronary artery bypass grafting in the Department of Veterans Affairs: a randomized clinical trial. JAMA Surg. 2022;157(4):303-310. doi:10.1001/jamasurg.2021.7578 35171210 PMC8851363

[22] Oddershede L, Andreasen JJ. Endoscopic vein harvesting for coronary artery bypass grafting is safe and reduces postoperative resource consumption. J Cardiovasc Dis Diagn. 2014;2(5):171-177. doi:10.4172/2329-9517.1000171

[23] Zenati MA, Bhatt DL, Stock EM, . Intermediate-term outcomes of endoscopic or open vein harvesting for coronary artery bypass grafting: the REGROUP randomized clinical trial. JAMA Netw Open. 2021;4(3):e211439-e211439. doi:10.1001/jamanetworkopen.2021.1439 33720367 PMC7961312

