# Supplementary material for: Costs of Endoscopic vs Open Vein Harvesting for Coronary Artery Bypass Grafting: A Secondary Analysis of the REGROUP Trial
Source: JAMA Netw Open. 2022 Jun 21;5(6):e2217686. doi: 10.1001/jamanetworkopen.2022.17686 (PMC9214587; doi:10.1001/jamanetworkopen.2022.17686)
Supplement: Supplement 1. — Trial Protocol and Statistical Analysis Plan [file jamanetwopen-e2217686-s001.pdf]

CSP# 588 – VERSION CONTROL LOG

Changes from Version 1, June 1, 2013 to Version 1.1, August 1, 2013		
**Page**	**Section**	**Description of Change**
All	All	Version number changed to 1.1 and issue date changed to August 1, 2013.
17	Schedule of Events	Form 00 from Contact Information changed to Screening Log.
17	Schedule of Events	Divided Form 06 into two separate forms – Form 06 Post Operative Assessments and Form 07 Discharge Assessments.
17	Schedule of Events	Renumbered the remaining forms.
19	Interim Follow-up	Removed the word "either" from the last sentence.
33	Sample Size Calculation	Changed the percent's were changed with regards to the proportion of subjects who will not experience MACE between the two treatment arms – 16.5% in the EVH arm was changed to 83.5% and 8.5% in the OVH arm was changed to 91.5%.
**Changes from Version 1.1, August 1, 2013 to Version 1.2, October 1, 2013**		
All	All	Version number changed to 1.2 and issue date changed to October 1, 2013
	Table of Contents	Removed CV's from Appendix C and replaced with "Definition of MI"
V	Executive Committee Members	Changed email address for Deepak Bhatt to DLBHATTMD@post.harvard.edu
9	Study Flow Diagram	Removed CIRB and replaced with IRB
15	Subject Assessments	Under "Initial Assessment of Coronary Arteries", added "In addition, the surgeon will be asked to describe a surgical plan indicating which bypass grafts are planned after reviewing the cardiac catheterization results"
16	Subject Assessments	Under "Post-operative and Diagnostic Test Evaluation", changed the collection of troponin levels to cardiac biomarkers – troponin levels or CPK levels are now both acceptable for the verification of an MI.

17	Schedule of Assessments	Changed timing of Form 14 “Termination” from Visit 49 to As Needed.<br><br>Changed Form 19 to “Confirmation of Myocardial Infarction by Local Site”.<br><br>Created form now called Form 20 “Confirmation of Myocardial Infarction by Clinical Events Committee”.<br><br>Renumbered Form 20 “Cause of Death” to Form 21.
19	Monitoring Serious Adverse Events	Under section d, removed SharePoint and replaced with DataFax system.
20	Subject Management	Made clarification regarding the post-op ECG results by adding the following: Per standard clinical care and most recent guidelines [Thyegesen 2012], cardiac biomarkers (preferably cardiac Troponin I or T) will be obtained if the 12-lead EKG demonstrates evidence of type 5 [Thyegesen 2012] myocardial infarction, including new pathologic Q waves or new Left Bundle Branch Block. The cardiac biomarker will be obtained every eight hours until a downward trend is seen in the level.
A-5	Informed Consent	Modified the collection of troponin levels (48 hours post-op) to be performed only if clinically indicated as follows::<br><br>“On the first and second day after your surgery, you will have an electrocardiogram - also known as an EKG. This test is routinely performed on CABG patients but the results will also be used for research purposes. If the EKG indicates there is new damage to your heart, known as a myocardial infarction (MI), then approximately two tablespoons of your blood will be drawn to check cardiac biomarkers. This is because cardiac biomarker levels will become increased in the blood when there is new heart damage. This test will be repeated every eight hours only if the level is increasing. Once it is determined the level is decreasing or remains in a normal range no further levels will be drawn. This blood test will help determine if you have had any new damage to your heart. Cardiac biomarker tests are frequently performed on CABG patients but the results will also be used for research purposes.”
A-8	Informed Consent	Modified section under “Information about you is protected in the following way” to read as follows:<br><br>Your research records will be kept indefinitely or until the law allows

		their destruction in accordance with the VA Record Control Schedule (www1.va.gov/VHAPUBLICATIONS/RCS10/rcs10-1.pdf). Records will be destroyed, when allowed, in the following manner.<br><br>Paper records will be shredded. Electronic records will be destroyed in a manner in which they cannot be retrieved. The data from this study will be entered into a VA CSP Data Repository managed by the VA CSP Data Coordinating Center in Perry Point, MD and used for future IRB approved research.
C-1	Definition of MI	New Appendix C to include the guidance for the definition of Type 5 Myocardial Infarction (MI related to CABG). Previous Appendix C, "Curricula Vitae" has been removed.
D-1	BRDP	Removed Craig Kreisler as statistical programmer and added Mike Beam as Computer Assistant
D-4	BRDP	Updated "Schedule of Assessments" table to reflect changes made to the protocol.
F-1	Vein Harvester Qualifications	Changed/modified former Appendix F: " Participation/Harvester Qualifications" and replaced with : "Site Selection Process: Vein Harvester Qualification Policy"

FORM REVISION RECORD
FOR
CSP# 588 REGROUP STUDY

**Changes from Version 1, Dated June 1, 2013 to Version 1.1 dated August 1, 2013**		
**FORM**	**Page**	**DESCRIPTION OF CHANGE**
1	ALL	Change to footer: Version # and date changed to “Version 1.1, August 1, 2013”
	1	Reworded instruction on Question 9 (“unless special approval obtained for enrollment” now reads “without special approval …”
	1	Removed “<” and added “less than” on Question 11.
	2	Changed instruction on Question 15 to: “mark an x in the one box that best describes the reason”
	2	Added an additional option to Question 15: “subject refused to sign informed consent”
	3	Changed instruction on Question 17 to: “mark an x in the one box that best describes the reason”
2	ALL	Change to footer: Version # and date changed to “Version 1.1, August 1, 2013”
	1	At top of form, changed “enrolled subjects” to “randomized subjects”
	4	Add Peak Troponin T to Question 26.
	4	Added to question 32: “If experiencing angina classify by CCSS: Class I, Class II, Class III, Class IV”
	7	Changed “Serum Potassium” (Question 61) to “Potassium” with option to check either “Serum” or “Plasma”
	7	Changed “GFR Calculated” (Question 64) to “eGFR (IDMS) < 60
	7	Added extra boxes to Question 66
	9	Added instruction to Question 80: “If none, skip questions 81-83, calculate total score and sign form”
	10	Removed “None” as an option for Questions 81 and 82.
3	ALL	Added “Visit No” to header and changed footer: Version # and date changed to “Version 1.1, August 1, 2013”
4	ALL	Added “Visit No” to header and changed footer: Version # and date changed to “Version 1.1, August 1, 2013”

5	ALL	Change to footer: Version # and date changed to “Version 1.1, August 1, 2013”
	2	Added details to Question 14 (Vein mapping). If yes, performed by, when performed, if preoperative, when.
	2	Added Questions 20 and 21: FiO2 and PO2
	3	Added Questions 27 and 28: FiO2 (mid-procedure) and PO2 (mid-procedure)
	4	Added Questions 38, 39, 40, and 41: PaCO2 (mid-procedure), ETCO2 (mid-procedure), FiO2 (mid-procedure), PO2 (mid-procedure).
	4	Added Questions 48 and 49: FiO2 and PO2
	5	Added Question 52: Vein harvester
	2 - 8	Renumber questions starting at question 20
6	ALL	Changed title from Postoperative and Discharge Assessment” to Postoperative Assessments”
	ALL	Change to footer: Version # and date changed to “Version 1.1, August 1, 2013”
	1,2	Moved question 2 and 13 “Troponin I” to question 21 “Peak Troponin I”
	1	Question 2 (changed from question 3) was “ECG evidence of ischemia” and now is “ECG evidence of new pathologic Q waves or new LBBB”
	2	Question12 (changed from question 14) was “ECG evidence of ischemia” and now is “ECG evidence of new pathologic Q waves or new LBBB”
	3	Questions 23 thru 31 (discharge assessments) were moved to a new form: Form 07 – Discharge Assessment
	1,2	Renumbered questions
7	ALL	New form: Discharge Assessment<br>Questions 23 thru 28 and questions 30 and 31 from Form 06 were moved to Form 07. Question 29 from form 6 “Coumadin” has been removed.
8	ALL	Previously form 07 remains unchanged except for new form number and changed footer: Version # and date changed to “Version 1.1, August 1, 2013”
9	ALL	Previously form 08 remains unchanged except for new form number and changed footer: Version # and date changed to “Version 1.1, August 1, 2013”
10	ALL	Previously form 09 changed to reflect new form number and changed footer: Version # and date changed to “Version 1.1, August 1, 2013”
	1	Removed Visit # on form and replaced with “Date of Discharge Assessment” for Questions 1 thru 4 and “Date of Follow-up Assessment” for Questions 5 thru 11.
11	ALL	Previously form 10 remains unchanged except for new form number and changed footer: Version # and date changed to “Version 1.1, August 1, 2013”

12	ALL	Previously form 11 remains unchanged except for new form number and changed footer: Version # and date changed to "Version 1.1, August 1, 2013". Visit # was added to header.
13	ALL	Previously form 12 remains unchanged except for new form number and Changed footer: Version # and date changed to "Version 1.1, August 1, 2013"
14	ALL	Previously form 13 remains unchanged except for new form number and changed footer: Version # and date changed to "Version 1.1, August 1, 2013". Visit # was added to header.
15	ALL	Previously form 14 changed to reflect new form number and changed footer: Version # and date changed to "Version 1.1, August 1, 2013"
	1	Question 5 added: "Brief description of the serious adverse event"
	1,2	Renumbered questions 6 thru 9. Added Question 10 "Is a Follow-up Serious Adverse Event Form required?"
16	ALL	Previously form 15 changed to reflect new form number and changed footer: Version # and date changed to "Version 1.1, August 1, 2013"
	1	Question 5 – changed "Recovering/Resolving" to "Ongoing – Recovering/Resolving", and changed "not Recovered/Not Resolved" to "Ongoing – Not Recovered/Not Resolved"
	1	Added Question 6 "Is another follow-up form expected for this SAE?"
17	ALL	Previously form 16 remains unchanged except for new form number and changed footer: Version # and date changed to "Version 1.1, August 1, 2013".
18	ALL	Previously form 17 changed to reflect new form number and changed footer: Version # and date changed to "Version 1.1, August 1, 2013"
	1	Moved question 2 "Event Number" into header of the form.
	1	Removed question 8.
19	ALL	Previously form 18 changed to reflect new form number and changed footer: Version # and date changed to "Version 1.1, August 1, 2013"
	1	Added MI # to the header information.
20	ALL	Previously form 19 remains unchanged except for new form number and changed footer: Version # and date changed to "Version 1.1, August 1, 2013".
**Changes from Version 1.1, Dated August 1, 2013 to Version 1.2 dated October 1, 2013**		
1	ALL	Change to footer: Version # and date changed to "Version 1.2, October 1, 2013"
	1	Question 5 modified to include the "availability of a participating surgeon available for the procedure"
2	ALL	Change to footer: Version # and date changed to "Version 1.2, October 1, 2013"
	1	Question 3: Race – added category of "Hispanic"

	4	Question 26: Changed Peak Troponin to: "Peak Cardiac Biomarkers" this includes Troponin I OR Troponin T OR, CPKs
	7	Question 51, "Stensos %": Added box to value
	7	Added Question 54, "Intent to bypass" and Question 54a "If yes, Type of conduit to be used"
	7-11	Renumbered questions 54 thru 85
	8	Question 66 "C-Reactive Protein" added box to value
3	ALL	No changes to form: Version # and date changed to "Version 1.2, October 1, 2013"
4	ALL	No changes to form: Version # and date changed to "Version 1.2, October 1, 2013"
5	ALL	Change to footer: Version # and date changed to "Version 1.2, October 1, 2013"
	3	Question 23: Added "If no, provide reason: ACT already therapeutic, Thrombocytopenia, Pre-op Plavix use, other specify".
	4	Question 35: Added instruction "add total length of incisions – not the bridging"
	8	Added new question 59: "Bypass graft completed as indicated on Form 2, Q54"
	8,9	Renumbered questions 60 thru 68.
6	ALL	Change to footer: Version # and date changed to "Version 1.2, October 1, 2013"
	1	Question 2: Added 2A, B, C: Cardiac Biomarkers
	3	Question 12: Added 2A, B, C: Cardiac Biomarkers
	4	Removed Question 21: "Peak Troponin I"
7	ALL	No changes to form: Version # and date changed to "Version 1.2, October 1, 2013"
8	ALL	No changes to form: Version # and date changed to "Version 1.2, October 1, 2013"
9	ALL	No changes to form: Version # and date changed to "Version 1.2, October 1, 2013"
10	ALL	Change to footer: Version # and date changed to "Version 1.2, October 1, 2013"
	2	Question 9: Added instruction "If yes, complete Form 15 – SAE"
11	ALL	Change to footer: Version # and date changed to "Version 1.2, October 1, 2013"
	1	Question 2: Changed instruction from "If yes, complete form 15" to "If yes, complete form 15 and 19"
12	ALL	Change to footer: Version # and date changed to "Version 1.2, October 1, 2013"
	1	Question 4: Changed instruction from "If yes, complete form 15" to "If yes, complete form 15 and 19"
13	ALL	No changes to form: Version # and date changed to "Version 1.2, October 1, 2013"
14	ALL	No changes to form: Version # and date changed to "Version 1.2, October 1, 2013"
15	ALL	No changes to form: Version # and date changed to "Version 1.2, October 1, 2013"
16	ALL	No changes to form: Version # and date changed to "Version 1.2, October 1, 2013"

17	ALL	No changes to form: Version # and date changed to "Version 1.2, October 1, 2013"
18	ALL	No changes to form: Version # and date changed to "Version 1.2, October 1, 2013"
19	ALL	Change to footer: Version # and date changed to "Version 1.2, October 1, 2013"
	1	Removed Question 3: Gender
	1	Changed Troponin levels to "Cardiac Biomarkers"
	2	Removed question 5 and created new form to capture this question (Form 20)
20	ALL	Change to footer: Version # and date changed to "Version 1.2, October 1, 2013"
	1	New Form "Classification of Myocardial Infarction". Questions from Form 19 have been removed from that form and are now recorded on the new Form 20.
21	ALL	Change to footer: Version # and date changed to "Version 1.2, October 1, 2013"
	1	Changed Form # from 20 to 21

VA Cooperative Studies Program Protocol #588

RANDOMIZED ENDO-VEIN GRAFT PROSPECTIVE - REGROUP TRIAL

October 1, 2013

Version 1.2

Study Chairperson: Marco A. Zenati, MD, MSc, FETCS, VAMC Boston, MA

- P R I V I L E G E D A N D C O N F I D E N T I A L -
Not to be Disseminated Beyond Official
Committee Function and Use

TABLE OF CONTENTS

Page

Glossary i

Executive Summary iii

Executive Committee v

I. Introduction and Background 1

II. Preliminary Research 5

III. Study Objectives 7
- A. Primary Endpoint 7
- B. Primary Hypothesis 7
- C. Secondary Objective 7
- D. Secondary Hypothesis 7

IV. Importance to the VA 7

V. Summary of the Study Design 8
- Figure 1. Study Flow Diagram 9

VI. Subject Population 10
- A. Inclusion Criteria 10
- B. Exclusion Criteria 10
- C. Recruitment and Screening 10

VII. Saphenous Vein Harvesting Techniques 11
- A. Technique of Open Vein Harvesting 11
- B. Technique of Endoscopic Vein Harvesting 12

VIII. Human Rights and Informed Consent 13

IX. Evaluation Procedures 14
- A. Screening 14
- B. Randomization 15
- C. Subject Assessments 15
  - Table 2. Schedule of Events 17

X. Post-Discharge Follow-up Assessments 18
- A. Six Week Visit 18
- B. Interim Follow-Up 18
- C. Monitoring Serious Adverse Events 18
- D. Missed Visits 19

E. Termination ... 19

XI. Quality Control Procedures ... 20
A. Standardization/Validation of Measurements ... 20
B. Subject Management ... 20
C. Protocol Noncompliance ... 21
D. Site Performance Monitoring ... 21

XII. Data Management and Case Report Forms ... 22
A. Assessments, CRFs and their Frequency of Administration and Collection ... 22
B. Data Collection and Data Entry ... 22
C. Study Documentation and Records Retention ... 23
D. Data Security Plan ... 23
E. Data Sharing Plan ... 24

XIII. Feasibility of the study within the VA System ... 25

XIV. Requirements for Participating Centers ... 25

XV. Study Organization, Administration and Monitoring ... 27
A. Monitoring Bodies ... 27
B. Monitoring Subject Safety ... 28
C. Monitoring Subject Intake and Probation/Termination of Participating Sites ... 28
D. Alternate Plan if Recruitment Goals are not Met ... 29
E. Monitoring Medical Center Performance ... 29
F. Monitoring of Safety, Efficacy and Futility ... 30

XVI. Good Clinical Practices ... 30
A. Role of GCP ... 30
B. Summary of Monitoring and Auditing Plans ... 30

XVII. Biostatistical Considerations ... 31
A. Expected Treatment Effects ... 31
B. Sample Size Calculation for the REGROUP Study ... 31
C. Duration of Study and Number of Participating Sites ... 32
D. Statistical Analysis Plan ... 32
E. Interim Monitoring ... 34
F. Criteria for Study Termination ... 34
G. Handling of Missing Data ... 35
H. Reporting of Any Deviations from the Original Statistical Plan ... 35

XVIII. Publications ... 35
A. Publication Policy ... 35
B. Planned Publications ... 36

XIX. References ... 36

Appendices

A. Informed Consent Document A-1

B. Budget B-1

C. Denfition of MI C-1

D. Biostatistical and Research Data Processing D-1

E. Research Data Forms E-1

F. Site Selection Process: Vein Harvester Qualifications F-1

	GLOSSARY
ABI	Ankle-Brachial Index
BIRLS	Beneficiary Identification Records Locator Subsystem
BMI	Body Mass Index
CABG	Coronary Artery Bypass Grafting
CEC	Clinical Events Committee
CI	Confidence Interval
CPRS	Computerized Patient Record System
CRF	Case Report Form
CRNP	Certified Registered Nurse Practitioner
CSPCC	Cooperative Studies Program Coordinating Center
CSPCRPCC	Cooperative Studies Program Clinical Research Pharmacy Coordinating Center
CSSEC	Cooperative Studies Scientific Evaluation Committee
DBMS	Database Management System
DCF	Data Clarification Form
DMC	Data Monitoring Committee
EACTS	European Association for Cardiothoracic Surgery
EC	Executive Committee
ESC	European Society of Cardiology
EVH	Endoscopic Vein Harvest
GCP	Good Clinical Practices
HDL	High Density Lipoproteins
HIPAA	Health Insurance Portability and Accountability Act
HRC	Human Rights Committee
ICF	Informed Consent Form
IRB	Institutional Review Board
ISMICS	International Society for Minimally Invasive Cardiothoracic Surgery
ITA	Internal Thoracic Artery
ITT	Intent-to-Treat
LAD	Left Anterior Decending
LAO	Left Anterior Oblique
LDL	Low Density Lipoproteins
LOI	Letter of Intent
MACE	Major Adverse Cardiac Events
MAR	Missing at Random
MDWS	Medical Domain Web Services
MI	Myocardial Infarction
OCT	Optical Coherence Tomography
OMT	Optimal Medical Therapies
OR	Operating Room / Odds Ratio
OVH	Open Vein Harvest
PA	Physician Assistant
PCI	Percutaneous Coronary Intervention
QA	Quality Assurance
QOL	Quality of Life
RAO	Right Anterior Oblique
RCT	Randomized Control/Clinical Trial
REGROUP	Randomized Endo-Vein Graft Prospective (CSP#588)
ROOBY	Randomized On/Off Bypass (CSP#517)
SAE	Serious Adverse Event

SAP	Statistical Analysis Plan
SAQ	Seattle Angina Questionnaire
SCIP	Surgical Care Improvement Project
SI	Site Investigator
SMART	Site Monitoring, Auditing and Review Team
SQL	Structured Query Language
SQWM	Surgical Quality Workflow Manager
STS	Society of Thoracic Surgeons
SVG	Saphenous Vein Graft
VA	Veterans Administration
VAMC	Veterans Affairs Medical Center
VASQIP	VA Surgery Quality Improvement Program
VCSS	Venous Clinical Severity Score
VINCI	Veteran Informatics and Computing Infrastructure
VSSC	VHA Support Service Center

EXECUTIVE SUMMARY

Background: Coronary artery bypass grafting (CABG) is the most common major surgical procedure in the United States with over 300,000 cases performed each year. To restore blood flow to the heart, vascular conduits from another part of the body are procured to create a bypass around critically blocked coronary arteries. The left internal thoracic artery is the conduit of choice for CABG due to its superior long-term patency. However, almost all patients referred for CABG require additional grafts to provide complete revascularization. This necessitates the harvest of other vessels, most commonly the saphenous vein which is used almost ubiquitously in contemporary CABG with an average of two vein grafts per CABG procedure. In the last 10 years, Endoscopic Vein Harvesting (EVH) has been recommended as the preferred method over the traditional open harvesting technique (OVH) because it provides a minimally invasive approach. However, more recent investigations indicate potential for reduced long-term bypass graft patency and worse clinical outcomes with EVH. The long term impact of EVH on clinical outcomes has never been investigated on a large scale using a definitive, adequately powered, prospective Randomized Clinical Trial (RCT) with long-term follow-up.

Objectives: The primary efficacy end point is the composite rate of death from any cause, myocardial infarction or repeat revascularization (Major Adverse Cardiac Events – MACE) *throughout the multi-year Study period*. Each randomized subject (either in the Endoscopic or in the Open vein harvesting group) will be followed after the index CABG to capture the time-to-MACE event, where an 'EVENT' will be defined as either death (all cause) or a myocardial infarction or a revascularization procedure during the follow-up period. The primary hypothesis is that a significantly smaller proportion of CABG subjects with vein grafts harvested by the open technique will experience a MACE event compared to CABG subjects with vein grafts harvested by the endoscopic technique during the follow-up period. The secondary efficacy end point is the MACE rate at *one and three-years post-CABG*. We believe that the proposed CSP# 588 REGROUP Trial will be uniquely positioned to fill a significant gap in existing knowledge regarding the long term MACE rates of EVH in CABG and improve the quality of the care we provide to our Veterans and more broadly to all patients undergoing coronary revascularization. In addition, we believe that CSP# 588 findings will significantly impact the VA and national cardiac surgery coronary revascularization guidelines.

Design: CSP #588 - REGROUP is a randomized, intent-to-treat, two-arm, parallel design, multicenter study. Cardiac Surgery Programs at Veterans Affairs Medical Centers (VAMC) with expertise in performing both EVH and OVH will be invited to participate in the study. Subjects requiring elective or urgent CABG using cardiopulmonary bypass with use of at least one SVG will be screened for enrollment using established inclusion/exclusion criteria. Enrolled subjects will be randomized to one of the two arms (EVH or OVH) after an experienced vein harvester is identified and assigned. Assessments will be collected at multiple time points including: baseline, intraoperatively, postoperatively, at discharge or 30 days after surgery if still hospitalized. Assessment of leg wound complications will be completed at the time of discharge and at six-week post-surgery. Telephone follow-ups will occur at three-month intervals post-surgery until the participating sites are decommissioned at the end of the trial period (which would be approximately 4.5 years after the site initiations). For long-term MACE outcomes, passive follow up for MACE using VA clinical and administrative databases (CPRS, VASQIP, etc) will be performed centrally by the Study Chair's office for another two years.

<u>Sample Size and Study Duration</u>: This study will enroll approximately 1150 subjects requiring CABG at 16 VA Medical Centers with expertise in both techniques of vein harvesting. Assuming an enrollment rate of two subjects/medical center/month, total enrollment will take approximately three years to complete. With at least one-year follow-up period for the last subject randomized and two additional years of passive follow-up by the chair's office, the total duration of the study will be approximately six and half years.

<u>Subject Population</u>: Any subject requiring a non-emergent CABG will be considered for entry into the study. Subjects who are hemodynamically unstable, have moderate to severe valvular disease or are unwilling or unable to provide informed consent will be excluded.

<u>Treatments</u>: *Open Vein Harvesting* is the traditional method of saphenectomy for CABG. It is performed under direct vision using a single long incision or, more commonly, multiple smaller incisions (referred to as "bridging" technique) along the course of the vein. This approach minimizes manipulation and direct trauma to the conduit but is associated with potential for discomfort and leg wound healing complications. *Endoscopic Vein Harvesting* is a minimally invasive procedure that was developed to eliminate the need for long incisions associated with OVH. EVH reduces the risk of wound infections and other leg wound complications but may be more traumatic to the conduit than OVH.

CSP#588
Randomized Endo-Vein Graft Prospective REGROUP Trial

Executive Committee Members

Marco A. Zenati, MD MSc FETCS
Professor of Surgery
Harvard Medical School
Chief of Cardiac Surgery, VABHCS
Boston, MA
Office: 857 203-6202
Email: Marco_Zenati@hms.harvard.edu

Rosemary F. Kelly, MD
Division of Cardiothoracic Surgery
University of Minnesota, MMC 207
420 Delaware Street, SE
Minneapolis, MN 55455
Office: 612-725-2148
Email: kelly071@umn.edu

Deepak L. Bhatt, MD MPH
Professor of Medicine, Harvard Medical School
Chief of Cardiology, VA Boston Healthcare System
1400 VFW Parkway, 5B113
West Roxbury, MA 02132
Office: 857-203-6840
Fax: 857-203-5550
Email: DLBHATTMD@post.harvard.edu

Jennifer M. Gabany, CRNP, MSN, CCRC
National Nurse Coordinator, CSP 588 REGROUP
Division of Cardiothoracic Surgery
VA Boston, MA
Email: Jennifer.Gabany@va.gov

Cindy L. Colling, R.Ph., MS
CSPCRPCC (151-I)
2401 Centre Avenue SE
Albuquerque, NM 87106-4180
Office: 505-248-3200
Email: Cynthia.colling@va.gov

Jerene M. Bitondo, PA-C
Chief Physician Assistant
Massachusetts General Hospital
Cox 630 55 Fruit Street
Boston, MA 02114
Office: 617-724-4825
Email: jbitondo@partners.org

Faisal G. Bakaeen, MD, FACS
Baylor College of Medicine,
One Baylor Plaza
Houston, TX 77030
Office: 713 794-7892
Email: fbakaeen@bcm.edu

J. Michael Gaziano, MD, MPH
MAVERIC (151 MAV)
VA Boston Healthcare System
150 South Huntington Avenue
Jamaica Plain, MA 02130
Office: 617-278-0848 (Brigham and Woman's Hospital) 857-364-9500, x4201 (VA)
Email: Michael.Gaziano@va.gov

Kousick Biswas, PhD
VA Maryland Healthcare System
CSPCC 151E
Building 362T
Boiler House Road
Perry Point, MD 21902
Office: 410-642-2411, x5283
Email: Kousick.Biswas@va.gov

I. INTRODUCTION AND BACKGROUND

In recent years, a consensus has emerged in the literature and in the Cardiac Surgical community that a definitive, adequately powered, prospective randomized controlled trial (RCT) is necessary to definitively assess the long-term clinical outcomes of CABG patients whose veins were harvested endoscopically. The need for a RCT was discussed first by the Duke Clinical Research Institute group in their seminal 2009 study published in the *New England Journal of Medicine* (Lopes 2009). Based on the results of a CSP #517 ROOBY sub-analysis, our group in Boston also recommended a RCT of EVH vs. OVH (Zenati 2011). Furthermore, an Editorial authored by the Committee that drafted the original 2005 ISMICS guidelines recommending EVH, has now expressed significant concerns on the long-term patency and adverse effects of EVH and recommends a RCT (Cheng 2010). Professor Angelini's group in Bristol, UK in an Editorial in *Nature Medicine* supports a large RCT on EVH vs. OVH (Patel 2009). Kempfert in an Expert Review of EVH discusses the need for a RCT with long-term outcomes to definitively assess EVH's safety profile (Kempfert 2011); in addition, Mariani from The Netherlands also advocates a RCT (Mariani 2011). All three external reviewers of our original Letter of Intent for CSP# 588 strongly supported our proposal for a RCT. We believe that a large multicenter prospective randomized trial taking into consideration important confounding factors will be indispensable before clearer guidelines can be formulated in favor or to the disadvantage of EVH.

Coronary artery bypass grafting is the most common major surgical procedure in the U.S. with over 300,000 cases performed each year. To restore blood flow to the heart, vascular conduits from another part of the body are procured to create a bypass around critically blocked coronary arteries. The internal thoracic artery (ITA) remains the conduit of first choice due to its superior long-term patency leading to superior clinical outcomes, which is the result of near-perfect integrity of its intima layer (Loop 1986). The left anterior descending (LAD) coronary artery is the most important coronary artery, supplying up to 70% of the left ventricle. Therefore, the left ITA-LAD bypass graft is currently the gold standard for surgical revascularization, with patency rates of 96% at one year, 90% at three years and 86% at 10 years (Loop 1989). However, almost all patients referred for CABG require additional grafts to provide complete revascularization. This necessitates the harvest of other vessels, most commonly the saphenous vein which is used ubiquitously in contemporary CABG with an average of two vein grafts per CABG procedure (Class IIa, Level B) (Brown 2010; Barner 2008).

The traditional method of vein harvesting with an open longitudinal incision along the course of the greater saphenous vein has been associated with complications including dehiscence, cellulitis, lymphangitis, drainage, edema, pain, hematomas, skin necrosis, and infection (Bitondo 2002). These in turn lead to delayed wound healing, increased length of hospital stay, higher cost of postop care, and greater patient discomfort. Bitondo and colleagues showed that OVH is associated with a 25% risk of leg wound complications, creating an important clinical and economic burden (Bitondo 2002; Goldsborough 1999). As an alternative, EVH was first introduced clinically in 1996 (Lumsden 1996) and has since been reported to reduce leg wound complications and improve patient satisfaction while decreasing resource utilization (Bonde 2005). Encouraging short-term (≤ six months) clinical (less wound morbidity, less pain, better cosmetic results, and improved patient satisfaction) and graft patency outcomes have been described (Andreasen 2008; Kiaii 2002; Perrault 2004; Puskas 1999; Yun 2004, Markar 2010). In a recent "Best Evidence Topic" study (Tennyson 2010), a review of the literature showed EVH reduced the level

of postop pain (pain score for EVH=0.52±0.95; OVH=1.02±1.51; p=0.03) and wound complications (range from 3% to 7.4% for EVH and 13% to 19.4% for OVH). These clinical benefits were associated with high levels of patient satisfaction.

Since its clinical introduction in 1996, EVH has increased in popularity to become the preferred method of SVG harvesting in the United States. According to the Society of Thoracic Surgeons' National Database (www.sts.org), EVH was utilized in ~80% of CABG procedures performed in the United States in 2008. A recent Editorial published in *The New England Journal of Medicine* predicted the "demise of open vein harvesting" in the near future in favor of EVH (Aranki 2009). In the U.S., EVH is almost universally performed by mid-level practitioners (Physician Assistants or Nurse Practitioners), rather than attending surgeons, residents, or fellows, and generally takes longer to learn than OVH. No universally accepted metrics for proficiency are available and training is usually provided by vendors. Cadwallader and associates undertook a systematic review and meta-analysis of the use of EVH vs. OVH in the United Kingdom. They concluded that EVH has a role in vein harvesting but is clearly *operator dependent.* EVH is therefore only preferable to OVH when performed by an experienced practitioner (Cadwallader 2009). EVH requires video equipment and a video tower for the endoscope. Before initiating EVH, some centers administer an intravenous bolus of 5,000 international units (IU) of unfractionated heparin to prevent vein thrombosis: this practice is based on limited evidence from a small, single-center study that showed a decrease in fibrin clots in veins harvested with EVH when a bolus of intravenous heparin was administered prior to the initiation of EVH (Brown 2007).

EVH can be performed with one of the commercially available systems that follow similar technical steps (VasoView®, available since 1996 and first marketed by Origin Med Systems, then by Boston Scientific/Guidant Cardiac Surgery, and currently by MAQUET Cardiovascular, Wayne, NJ www.maquet.com [estimated EVH U.S. market share 90%]; Virtuosaph™, introduced in 2005, Terumo Cardiovascular Systems Corporation, Ann Arbor, MI www.terumo-cvs.com/virtuosaph [approximate estimated EVH U.S. market share 8%]; all using CO_2 insufflation for visualization and dissection. Briefly, a 1.5 to 2.0 cm incision is made medially above or below the knee, depending on the length of the vein required. Harvesting is performed under video guidance with a rigid endoscope and is directed towards the groin region as far proximally as possible. If a longer segment of a vein is required, the endoscope may also be directed distally through the same incision. Side branches are divided by using bipolar cauterizing scissors or a bisector. EVH requires CO_2 to insufflate the subcutaneous cavity in the lower extremity and frequent use of bipolar cautery in the vicinity of the saphenous vein in order to divide the side branches; neither insufflation nor bipolar energy is required for OVH. The use of cautery has been proposed to cause thermal injury to the vessel wall, which may impair the graft quality by compromising the viability of endothelial cells and resulting in platelet aggregation and thrombosis. Encouraging short-term (≤six months) clinical and graft patency outcomes have been described (Andreasen 2008; Kiaii 2002; Perrault 2004; Puskas 1999; Yun 2004).

There is concern that relatively longer manipulation times and the use of rigid devices in EVH may cause direct mechanical injury to vein grafts. Traditional surgical principles for handling vascular tissue emphasize a "no-touch" approach during dissection to minimize the risk of intimal endamage (Gundry 1980). EVH inherently requires forces to be applied to the vein that are usually avoided in open harvest, including traction, adventitial stripping and venous compression. There is a lack of unanimity on

the role of EVH on premature graft loss (Ouzunian 2010) which may be explained by variability of techniques and level of experience among centers. Desai and associates, in a prospective pilot study using Optical Coherence Tomography (OCT) imaging, noted that veins procured by novice harvesters had nearly 50% more discrete injuries than veins procured by experienced harvesters (Brown 2007). Rousou and colleagues recently reported that, compared to OVH, EVH adversely affects vein endothelial function (Rousou 2009). They used epifluorescence multiphoton microscopy (a technique that measures endothelial viability and functionality in real time with greater sensitivity than other methods) and demonstrated endothelial and smooth muscle cell damage in vein grafts with reduced endothelial cell viability, attenuated calcium mobilization, and nitric oxide production in the EVH group. Endothelial dysfunction enhances thrombogenicity and may lead to early thrombosis and accelerated SVG failure. Compromised endothelial integrity is the primary determinant in the interrelated pathogenesis of thrombosis, intimal hyperplasia and atherosclerosis within the SVG (Thatte 2001). Considering all the basic science and clinical arguments, together with increased risk of adverse events that inevitably follows graft failure, the goal of harvesting SVG with as near-perfect integrity of its intima layer as possible seems prudent until available evidence demonstrates otherwise.

Because the enthusiastic adoption of EVH preceded any professional consensus on this topic, in 2005 an *ad hoc* Committee of the International Society for Minimally Invasive Cardiothoracic Surgery (ISMICS www.ISMICS.org) published a consensus statement on the use of EVH vs. OVH in CABG (Allen 2005). Based on reports of short-term comparable rates of MACE, angiographic SVG patency, and quality of the harvested conduit with the two techniques in both randomized and non-randomized trials, the Members of the ISMICS Consensus Committee suggested that either EVH or OVH can be used to procure SVG conduits for CABG. In addition, the Consensus Committee also recommended that EVH be the "standard of care" (Class I, Level A) in order to reduce wound-related complications, improve patient satisfaction, and to decrease postop pain, length of hospital stay, and use of outpatient wound-management resources. EVH is listed in a respected reference textbook entitled: "Evidence Based Cardiology –Third Edition" edited by Yusuf as a Class IIa (Class of Recommendation IIa: conflicting evidence and/or divergence of opinion about the efficacy with weight of evidence in favor of efficacy) recommendation based on level B evidence (Brown 2010).

Based on widespread concerns for excessive trauma to the SVG during EVH, in July of 2009, Lopes and associates from the Duke Clinical Research Institute (DCRI) reported in *The New England Journal of Medicine* on the long-term follow-up results of the Project of Ex-vivo Vein Graft Engineering via Transfection IV trial (PREVENT-IV) (Lopes 2009). The rate of vein-graft failure was significantly higher in those subjects who underwent EVH (46.7% vs. 38.0 %; odds ratio 1.45, 95% CI 1.20-1.76). EVH was also associated with a significantly higher combined rate of mortality, myocardial infarction, and repeat revascularization three years after surgery (20.2% vs. 17.4%; adjusted hazard ratio 1.22, 95% CI 1.01-1.47). The Authors' findings constituted the first published report of EVH resulting in poorer clinical outcomes than the OVH technique. For the first time, EVH was found to be *independently associated* with vein graft failure and adverse clinical outcomes. This paper also provided important long-term follow-up data that contradicted accepted clinical practice and called into question the wisdom of the ISMICS recommendations (Allen 2005). The DCRI Group recently reported an additional sub-analysis of the PREVENT-IV study and reported no difference in angiographic or 5-year clinical outcomes in patients who underwent open versus closed tunnel endoscopic harvesting (47.1% vs. 43.8% p=0.72; 24.5% vs.

26.8% p=0.26) (Van Diepen 2013). These findings suggest that the increased risk associated with EVH reported in the previous PREVENT-IV analysis does not seem to be associated with a specific endoscopic harvesting device.

A meta-analysis of 102 studies (including Lopes'), published in 2010, compared EVH to OVH in CABG (Markar 2010). Results of this meta-analysis showed that long-term graft patency in SVG harvested by OVH was better than those harvested by EVH (pooled odds ratio = 1.25, p=0.0039).

More recently, the Task Force on Myocardial Revascularization of the European Society of Cardiology (ESC) and the European Association for Cardiothoracic Surgery (EACTS) published their updated Guidelines on Myocardial Revascularization (Wjins 2010). According to this consensus statement, incorporating new evidence accumulated since 2009, "endoscopic vein graft harvesting cannot be recommended at present as it has been associated with vein graft failure and adverse clinical outcomes". This statement contradicts the 2005 ISMICS guideline and accepted clinical practice. Given the potential implications of the long-term impact of SVG graft failure on CABG outcomes, the role of EVH is currently the subject of substantial controversy in the literature (Aranki 2009; Cheng 2010; Connolly 2009; Patel 2009; Tennyson 2010).

It is well established that vein graft failure (i.e. severe graft stenosis or occlusion) adversely affects long-term clinical outcomes after CABG (Lopes 2012; Buxton 2009; Halabi 2005). In CSP #517 ROOBY, ineffective revascularization (defined as presence of non-FitzGibbon "A" graft to one of the main coronary territory) was associated with worse composite clinical outcomes at one-year (Table 1) (Hattler 2012).

Table 1. ROOBY 1 Year Graft Patency Results

Endpoint	One or more ineffectively revascularized territories no./total no. (%)	All 3 coronary territories effectively revascularized no./total no. (%)	Relative Risk (95% CI)	*p* value
1-yr composite endpoint*	96/587 (16.4)	46/778 (5.9)	2.77 (1.98 to 3.87)	< 0.001
1-yr non-fatal acute MI	60/587 (10.2)	33/778 (4.2)	2.41 (1.60 to 3.63)	< 0.001
1-yr repeat revascularization	54/587 (9.2)	14/778 (1.8)	5.11 (2.87 to 9.11)	< 0.001

FitzGibbon described clinical outcomes and vein graft failure rates among 1,388 patients who underwent a first CABG surgery from 1969-1994 (FitzGibbon 1996). This study showed that both mortality and vein graft failure rates increased over time, particularly seven years after CABG. At five years, survival rates were around 94% and vein graft failure rate was 25%. Halabi and associates found that early vein graft failure was associated with worse long-term outcomes of death, myocardial infarction and revascularization and that these results were driven by early revascularization (Halabi 2005). In their study, vein graft failure was the strongest predictor of the composite clinical outcome at 10 years. In the PREVENT-IV Trial, vein graft failure occurred in 787 of 1,829 patients (43%) [Alexander

2005]. Using the same PREVENT-IV Trial database, Lopes and associates studied data from 1,829 patients who underwent CABG surgery and had an angiogram performed up to 18 months following surgery (Lopes 2012). They demonstrated that vein graft failure was associated with an increased risk for the composite of death, myocardial infarction or repeat revascularization with an adjusted HR of 5.23. The composite outcome was driven by high rates of repeat revascularization in the patients with vein graft failure. It should be noted that in all of these studies, ITA-LAD was used in the vast majority of patients (>93%) according to the predominant practice and recommendations (Class I, Level C) (Brown 2010).

II. PRELIMINARY RESEARCH

Results of the VA CSP #517 ROOBY trial were recently published (Shroyer 2009). We have previously published the results of two preplanned sub-analyses of the ROOBY trial (Zenati 2006; Zenati 2007). More recently, we published the results of a pre-planned sub-analysis examining clinical and SVG patency outcomes in ROOBY trial patients who underwent either EVH or OVH (Zenati 2011). From February 2002 through April 2007, the ROOBY trial enrolled 2,203 patients. Beginning in April 2003, prospective collection of data regarding SVG harvesting technique was begun. A total of 1,471 patients (564 EVH, 907 OVH) had the harvesting technique recorded and had a SVG used as a bypass conduit. The 30-day composite end point was known for all these patients. One-year composite follow-up was determined for 96% (555 EVH, 859 OVH) of these patients. Follow-up angiography was obtained in 894 subjects (341 EVH, 553 OVH). For both the population of patients with their SVG harvest approach recorded (n = 1,471) and the sub-set with one-year cardiac catheterization (n = 894), the pre-operative patient characteristics were generally balanced between the EVH and the OVH groups. Almost all patients were male (99%), a reflection of the VA cardiac surgical patient population. Sixty-eight percent of patients had three vessel coronary artery disease and approximately 83% of patients had preserved left ventricular function. Less than 15% of study patients required urgent surgery. The quality of the harvested SVG was assessed as “good” in 81.6% of EVH and 85.6% of OVH patients, “intermediate” in 15.7% of EVH and 12.7% of OVH, and “poor” in 2.8% of EVH and 1.7% of OVH (p = NS). For the short-term composite end point there was no significant difference between EVH vs. OVH. More OVH than EVH patients (1.3%) suffered renal failure (1.3% vs. 0.0%; p=0.01) and more OVH patients needed new mechanical support (1.7% vs. 0.4%; p=0.02). There were no differences between EVH and OVH groups with respect to one-year composite outcome. For the subgroup of patients with one-year cardiac catheterization results, the rate of repeat revascularization was significantly higher in the EVH group than in the OVH group (6.7% vs. 3.4%, p<0.05), while the rates of non-fatal MI or death were similar. A mean of 2.02 SVGs were placed per patient, and a total of 1,807 SVGs were assessed for patency. The incidence of a patient having one or more occluded SVGs on follow-up angiography was 41.3% in the EVH group compared with 28.0% in the OVH group (p<0.0001). Overall SVG patency was 74.5% in the EVH group, significantly worse than the 85.2% rate in the OVH group (p<0.0001). Using multi-variable regression analysis, EVH was not identified as an independent and statistically significant predictor of the one-year composite outcome after holding other factors constant. Further, no interaction was identified between the SVG harvesting technique (EVH vs. OVH) and the use of an on-pump vs. off-pump approach (p = NS). Additionally, sensitivity analysis found no differential EVH vs. OVH impact for high volume vs. low volume EVH centers.

In conclusion, our sub-analysis of the CSP #517 dataset demonstrated that the one-year SVG occlusion rate was 25.5% in the EVH group and 14.8% in the OVH group (p<0.001). The MACE rate was 8.2% in the EVH group vs. 4.8% in the OVH group (p=0.061), and the rate of repeat revascularization by PCI or redo-CABG was 6.7% in the EVH group vs. 3.4% in the OVH group (p<0.001). There was no interaction between EVH and on- or off-pump CABG by multivariate logistic regression analysis. Both the PREVENT-IV and ROOBY sub-analyses suffer from the same limitation: the basis for randomization was not the SVG harvest modality (EVH vs. OVH). In addition, neither study accounted for other potentially important variables, such as device-related and experience-related EVH variables.

Furthermore, we recently published a meta-analysis of long-term EVH vs. OVH graft patency including the results of our recent publication (Zenati 2012); in the five long-term observational studies from 1996 to 2011 included in our analysis, SVG failure was expressed as the combination of angiographic occlusion and severe stenosis (FitzGibbon grades B+O). There were a total of 6,866 SVGs assessed by angiography in the five pooled studies. Both random and fixed effects models showed significantly lower graft patency with EVH. The random effect pooled OR was 1.62 (95% CI = 1.22 – 2.15; p = 0.0009) and the fixed effect pooled OR was 1.49 (95% CI = 1.33 – 1.68; p<0.0001) favoring OVH. We concluded that available evidence consistently identifies compromised SVG patency when the conduit was harvested with the EVH technique.

Because of safety concerns regarding endoscopic vein-graft harvesting, the US Food and Drug Administration issued a request to analyze the Society of Thoracic Surgeons Adult Cardiac Surgery Database for endoscopic and open vein-graft harvesting–related outcomes. The resulting study was published in JAMA in 2012 (Williams 2012). In this study 235,394 Medicare patients undergoing isolated CABG surgery in 934 surgical centers between 2003 and 2008 were examined with a median 3-year follow-up. Fifty-two percent of patients received endoscopic vein-graft harvesting. In a propensity score–adjusted analysis that minimized the influence of confounding between groups, there were no significant differences between endoscopic vein-graft harvesting and open vein-graft harvesting in 3-year mortality (13.2% vs 13.4%, respectively) or a composite of death, myocardial infarction, and revascularization (19.5% vs 19.7%, respectively). Compared with open vein-graft harvesting, endoscopic vein-graft harvesting was associated with a 13% lower harvest wound infection rate. Multiple sophisticated statistical techniques including sensitivity and subpopulation analyses confirm the robustness of the central findings. The study by Williams et al is important for several reasons. First is its sheer size and statistical power. Second, this investigation represents a snapshot picture of contemporary CABG surgery in the United States because it includes so many diverse sites with widely varying practice styles. The basic finding is that endoscopic vein-graft harvesting shows no difference in long-term mortality or need for revascularization.

Taken together, the major subanalysis studies cited above (PREVENT-IV, ROOBY and STS) only establish uncertainty (or *equipoise*) over the safest and most effective vein harvesting technique for CABG over a long-term follow up and lay the foundations for our proposed randomized study.

III. Study Objectives

A. **Primary Endpoint:**

To investigate the impact of SVG harvesting techniques – OVH vs. EVH on MACE, a composite end point of all-cause mortality, nonfatal myocardial infarction and repeat revascularization, over the active follow-up period of the study postoperatively .

B. **Primary Hypothesis:**

A significantly smaller proportion of CABG subjects with SVGs harvested by open technique will experience MACE post-surgery compared to CABG subjects with SVGs harvested by endoscopic technique during the active follow-up period.

C. **Secondary Objective:**

i. To investigate the impact of SVG harvesting techniques – OVH vs. EVH on MACE, a composite endpoint of all-cause mortality, nonfatal myocardial infarction and repeat revascularization, at one and three-year postoperatively .

ii. To investigate the impact of SVG harvesting techniques – OVH vs. EVH on MACE, a composite endpoint of all-cause mortality, nonfatal myocardial infarction and repeat revascularization, over the entire follow-up period (active and passive) of the study postoperatively.

D. **Secondary Hypothesis**:

i. One-year composite MACE rate will be 6 percentage points lower in the open harvesting group and three-year composite MACE rates will be at least 8-10 percentage points lower in the open vein harvesting group compared to the endoscopic vein harvesting group.

ii. A significant smaller proportion of CABG subjects with SVGs harvested by open technique will experience MACE post-surgery compared to CABG subjects with SVGs harvested by endoscopic technique during the entire follow-up period

Other objectives are:

1. Investigate the impact of the two harvesting techniques - open vs. endoscopic - on clinical indicators of leg wound complications and subject satisfaction at six-weeks post-surgery; The hypothesis is the leg wound complications will be lower and satisfaction will be higher in the EVH group compared to OVH group.
2. Compare subject quality of life scores according to the SVG harvest technique at six-week post-surgery; the hypothesis is the subjects' quality of life scores will be higher in the EVH group compared to the OVH group.
3. Determine the role of vein harvester experience on clinical outcomes

IV. Importance of the Study Topic to the VA and Its Patients

CABG is the most common major surgical procedure in the U.S. and the VAMC Health System, and EVH is now the preferred modality of SVG harvesting in both the private sector and the VAMC (72%

EVH adoption rate in the VHA based on a 2009 survey commissioned by Dr. Gunner at VACO). Decisions regarding the choice of coronary revascularization (CABG vs. percutaneous coronary intervention [PCI]) rest primarily on the anticipated failure rate of CABG versus PCI. The recent ARTS II trial (Serruys 2010) showed comparable freedom from MACE in selected patients undergoing multi-vessel PCI versus CABG.

In order to offer the safest and most durable revascularization strategy for veterans requiring CABG surgery, it is imperative to provide definitive evidence on the long-term clinical outcomes of EVH in order to minimize harvest site morbidity (e.g. leg wound infection, hospital readmission, pain, mobilization, and appealing cosmetic results) while preserving long-term clinical outcomes. The quality of the harvested conduit is an important aspect to consider when comparing harvesting techniques. These features ultimately determine long-term patient morbidity and mortality rates following CABG surgery (Buxton 2009).

V. Summary of the Study Design

This study is a randomized, intent-to-treat, two-arm, parallel design, multicenter study to compare clinical outcomes of major adverse cardiac events (MACE) of CABG subjects treated with SVG harvested with EVH and OVH during the trial period. Cardiac Surgery Programs at Veterans Affairs Medical Centers (VAMCs) with expertise in performing both EVH and OVH will be invited to participate in the study (EVH Program established for more than two years and at least 100 successful EVH cases performed by each mid-level provider or other designated individual involved with the study) (Desai 2011). Subjects requiring elective or urgent CABG using cardiopulmonary bypass with use of at least one SVG will be screened using established inclusion/exclusion criteria. Subjects will be randomized to one of the two arms (EVH or OVH) after an experienced vein harvester is identified and assigned to the case. Assessments will be collected at multiple time points including: baseline, intraoperatively, postoperatively, at discharge or 30 days after surgery if still hospitalized. Assessment of leg wound complications will be completed at the time of discharge and at six-week post-surgery. Quality of life self- assessments will be completed at baseline, six weeks, and one year (by mail or telephone). Telephone follow-ups will occur at three-month intervals post-surgery until the participating sites are decommissioned at the end of the trial period (which would be approximately 4.5 years after the site initiations). For long-term MACE outcomes, passive follow up for MACE using VA clinical and administrative databases (CPRS, VASQIP, etc.) will be performed centrally by the Study Chair's office for another 2 years.

The other secondary outcome measures are MACE at one and three-year post surgery, leg wound healing complications and Quality of Life.

Two leading vendors (Maquet and Terumo) are currently marketing EVH device technologies for CABG. Device-related and procedural information will be collected during the study.

Subjects will receive concomitant optimal medical therapies (OMT) in both groups as recommended by the current 2011 American College of Cardiology/American Heart Association guidelines [Smith 2011). OMT will include formal smoking cessation counseling and the administration of aspirin, beta blockers, angiotensin converting enzyme inhibitors, and lipid-lowering medications.

FIGURE 1. **STUDY FLOW DIAGRAM**

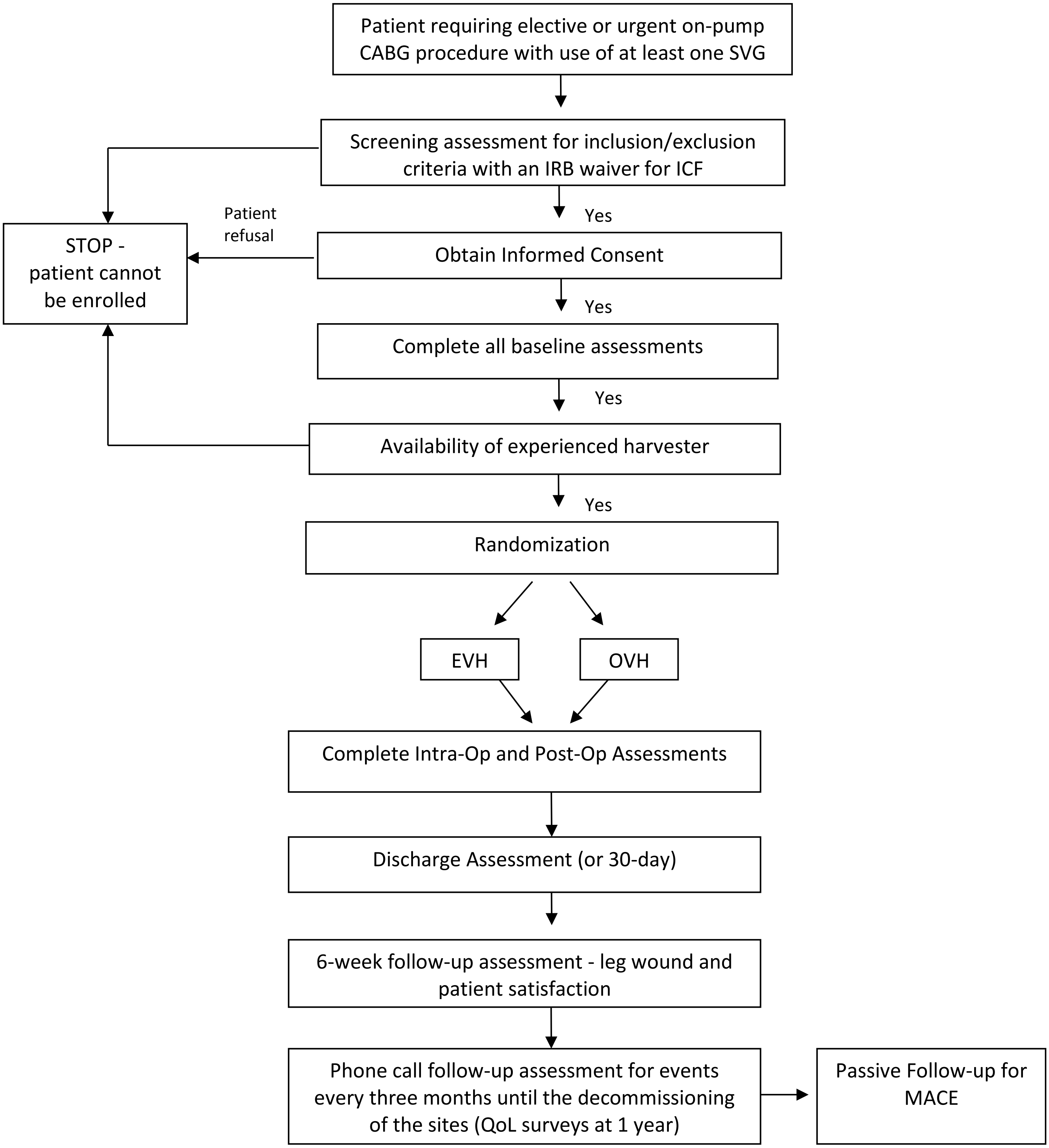

VI. Subject Population

All subjects who are candidates for CABG and who will undergo surgery at a VAMC with demonstrated expertise for both OVH and EVH and qualify to participate in CSP-sponsored research will be invited to participate in the REGROUP study.

A. Inclusion Criteria:

- Age 18 years or older
- Elective or Urgent CABG-only
- Median sternotomy approach
- At least one coronary bypass planned using saphenous vein graft for conduit
- Experienced EVH/OVH harvester and participating surgeon available for procedure

B. Exclusion Criteria:

- Combined valve procedure planned
- Moderate or severe valve disease (see definition of moderate/severe valve)
- Hemodynamically unstable or in cardiogenic shock
- Enrolled in another therapeutic or interventional study
- Off-pump CABG procedure planned
- Limited life expectancy < 1 year
- History of lower extremities venous stripping or ligation
- Inability to provide informed consent

C. Recruitment and Screening

We propose a prospective, multicenter, two-arm, randomized clinical trial (RCT). A block randomization technique will ensure equal distribution of subjects, within each harvester, within each site, in both arms of the trial. The randomization schema will be generated using SAS (SAS, Cary, NC).

Participating sites will be chosen based on availability of EVH/OVH harvesters (see Appendix F), but also based on CABG volume as well as ability and willingness of the local site surgeon investigator to meet enrollment goals. The local site surgeon investigators and research coordinators will be trained on study specific recruitment at the Investigator Meeting. Subsequently, each local site surgeon investigator will present the study protocol to the appropriate local clinical care providers for subjects undergoing CABG surgery at their site so those involved in the care of the subjects are familiar with the protocol requirements. Each site will have one full time dedicated research coordinator to facilitate enrollment. Each site will obtain a waiver of informed consent/HIPAA for screening purposes from the local IRB and following this, will work with the clinical care team to identify subjects referred for CABG surgery who meet study criteria. Every CABG-only subject the site surgeon investigator deems as

meeting study criteria will be approached. After preliminary consultation and screening with a potential participant's clinical team, the participating site's research coordinator will approach each non-emergent subject scheduled for a CABG-only procedure to discuss enrollment in the REGROUP study. After providing the potential subject adequate time to read the documents and to ask any questions, the research coordinator will obtain informed consent and collect all the baseline assessments. Randomization to either the EVH or OVH technique, stratified by harvester within site, will occur at the time of surgery after a qualified harvester is assigned to the case.

We recognize the VA policy to include women and minorities in clinical research. Although we do not anticipate a large number of women or minorities to be recruited for this study due to the demographics of subjects receiving care at the VA, efforts will be made to recruit both women and minorities for the REGROUP trial. At present, there are no ongoing or submitted research studies that directly relate to the REGROUP trial.

VII. Saphenous Vein Harvesting Techniques

The greater saphenous vein lies in the subcutaneous fat on the medial aspect of the leg from the sapheno-femoral junction to the medial malleolus. Below the knee, the vein is accompanied by the saphenous nerve which should be preserved during harvest. The vein is usually larger proximally with a thicker, more fibrotic wall, while distally the vein is healthier but may be small. The vein wall is thicker than that of an arterial conduit and the media is nourished by the *vasa vasorum* so that smooth muscle necrosis is usual after harvesting and it is replaced by fibrous tissue that converts the vein into a rather rigid tube. This obviously restricts vasomotion after grafting. The endothelium of the vein is frequently damaged or lost even with meticulous harvesting and preservation (Barner 1990) but regenerates over weeks (Busch 1986). Immediately prior to harvest, the lower extremities are circumferentially prepped with antiseptic solution (povidone-iodine or Hibiclens®) and the feet are placed in sterile stockinettes. Vein mapping is not used routinely and the selection of extremity is determined by presence of varicosities, any previous surgery, and the quality of skin and tissue.

A. Technique of Open Vein Harvesting

The conventional open harvesting technique requires an incision the length of the vein to be harvested or alternatively can be achieved by multiple small incisions ("bridging"). Dissection should be as atraumatic as possible, forceps should handle only the adventitia of the vein and stretch trauma minimized. Branches are controlled with clips or ligature. Cautery is *rarely* used during OVH to divide vein branches, but only to provide wound hemostasis before the incision is closed. Following harvest, the vein is marked by placing a soft vascular bulldog clamp on the new distal end or by cannulating the new proximal end. The vein is gently distended with a physiologic solution at low pressure (no more than 150mmHg, ideally using specially designed pressure-limiting syringes). Closure of the incision is performed in layers with absorbable sutures followed by subcuticular closure. At the end of the operation, the leg wound is typically covered with a cotton gauze dressing and an elastic ace wrap is applied to the entire leg. The "bridging" OVH technique variant was shown to be associated with similar rates of leg wound complications versus open OVH (Carpino 2000) and requires more technical expertise and a longer training period.

The OVH technique is highly reproducible and can be performed by surgical trainees, including medical students, and mid-level providers like Physician Assistants (PA) or Certified Registered Nurse Practitioners (CRNP). OVH is associated with variable (2-18%) degrees of morbidity, such as wound infection, especially in high-risk patient subsets (obese, diabetics, females), non-infective wound healing disturbances, postoperative pain, and poor mobility. Such morbidity prolongs the length of hospital stay, increases health care costs, and reduces patient satisfaction (Carpino 2000; Markar 2010).

B. Technique of Endoscopic Vein Harvesting

The preparation for EVH is similar to OVH but there is an additional requirement for video equipment and a video tower for the endoscope. Before initiating EVH, some centers administer 5,000 IU of intravenous heparin to prevent SVG thrombosis: this practice is based on limited evidence from a small, single center study that showed a decrease in fibrin clots in SVG harvested with EVH when low-dose heparin was used (Brown 2007). EVH can be performed with one of the commercially available systems that follow similar technical steps (VasoView®, available since 1996 and first marketed by Origin Med Systems, then by Boston Scientific/Guidant Cardiac Surgery, and currently by MAQUET Cardiovascular, Wayne, NJ www.maquet.com [estimated EVH U.S. market share 90%]; Virtuosaph™, introduced in 2005, Terumo Cardiovascular Systems Corporation, Ann Arbor, MI www.terumo-cvs.com/virtuosaph [approximate estimated EVH U.S. market share 8%]; all using CO_2 insufflation for visualization and dissection (the system is "open" for the Terumo device). Briefly, a 1.5 to 2.0 cm incision is made medially above or below the knee, depending on the length of the vein required. Harvesting is performed under video guidance with a rigid endoscope and is directed towards the groin region as far proximally as possible. If three segments of vein are required, the endoscope may also be directed distally through the same incision. Side branches are divided by using bipolar cauterizing scissors or a bisector. EVH requires CO_2 to insufflate the subcutaneous cavity in the lower extremity and frequent use of bipolar cautery in the vicinity of saphenous vein in order to divide the side branches; neither insufflation nor bipolar energy is required for OVH. The use of cautery has been proposed to cause thermal injury to the vessel wall, which may impair the graft quality by compromising the viability of endothelial cells and resulting in platelet aggregation and thrombosis (Rousou 2009). After the vein has been freed circumferentially from surrounding tissue and all branches have been divided, a small puncture is made under endoscopic guidance proximally over the SVG. The proximal end is clamped, divided, and then ligated. After removing the vein from the leg, SVG is gently distended manually with a distending solution and the side branches are ligated with 4-0 silk ties. Any avulsed branches are either repaired by carefully approximating the adventitial layer with 7-0 Prolene sutures or excluded if fortuitously located between vein graft segments. The incision is closed with absorbable subcutaneous and subcuticular sutures and then wrapped with an elastic ace bandage. The SVG is then placed in a storage solution (i.e. heparinized blood, physiological pH balanced salt solution or GALA solution) until it is ready for use. Incisions are closed after harvest with or without drains.

Best practice EVH technique will be recommended at all sites and will include:
Optimal preoperative preparation, including vein mapping whenever possible, plan the EVH procedure as having 3 separate stages: (a) choosing the incision site and making the incision, (b) dissecting the vessel and vessel tributaries, and (c) dividing the vessel branches.

Incision: decide on the best place to make the incision and mark the site, keep the length of the skin incision to a minimum, consider making the incision to correspond with tension lines of the skin.
Heparin: an intravenous heparin bolus of a minimum of 1,000IU to a maximum of 5,000IU will be used at the beginning of the harvest.
CO_2 insufflation: use the lowest tunnel pressure possible to reduce the risk of CO_2 embolism, monitor central venous pressure, use appropriate monitoring to be alerted to CO_2 –related events. The trocar cuff should be kept delated or minimally inflated to avoid interruption of blood flow inside the SVG.
Dissection of the vessel: establish a regular sequence of dissection; use short, gentle motions, ensure that side branches are thoroughly dissected to allow adequate length during branch division, apply appropriate pressure with the opposing hand to promote ease of dissection along the vessel
Division of branches: establish a regular sequence for dividing the branches, consider making a fasciotomy along the tunnel if the space is very tight, before dividing the branch consider whether it is of adequate length to clip or tie, keep energy settings as low as possible during branch division
Vessel removal and preparation: make sure all branches and connective tissue are free from the vein before removing it, use appropriate technique for distal ligation of the vessel, take care **not to stretch** the vessel when removing it from the tunnel, once the vessel is extracted and prepared, place it in the specified solution until ready for use in the surgery.

VIII. Human Rights Issues and Informed Consent

After a subject has been deemed eligible for the trial through screening, the research coordinator or site investigator will obtain informed consent from the subject. After the clinical care team meets with the subject to discuss the CABG procedure the research coordinator at each site will introduce him/herself and explain the study to the subject and present the detailed consent form if the subject gives permission to discuss the research study. The consenting process for the research study will occur at a separate time point, after the informed consent for clinical care has been completed. Subsequently, the site investigator or a designee will review and discuss the study with the subject and answer any questions that the subject might have. The general purpose of the study, along with detailed information about the treatment comparisons, the randomization process, the study timeline, including what is expected of the subject, and the rights of study subjects will be clearly described. The harvesting techniques (both EVH and OVH) and the associated risks with the techniques will also be addressed. The importance of subject confidentiality will be stressed, and the process for maintaining confidentiality will be described. This discussion will be held in an area that provides the subject time to focus on reading information about the study and asking questions without feeling rushed or uninformed. Any family member the subject requests to be present will be included. At this time, the surgeon(s) participating in the study will also meet with the subject to discuss the study and answer any questions about the two different types of procedures for vein harvesting that might be performed.

The site investigator or research coordinator will ensure that the subject understands every aspect of the trial, including its risks and benefits, prior to signing the informed consent.

If the subject agrees, his/her consent to participate in the study will be recorded on the CSP# 588 Informed Consent Form (VA form 10-1086, See Appendix A – Informed Consent Form). The original will be kept in the site investigator's study file for that subject and a copy will be placed in the subject's

medical record. Copies of the signed consent form will be provided to the subject, the Research Office at the participating site (if required by the IRB), and faxed or mailed via UPS/FedEx to the Perry Point CSPCC at the time of enrollment in the study.

Informed consent requires that the subject understand the details of the study and agrees, without coercion, to participation in the study. To obtain informed consent, the following information shall be provided to each subject:

- Name of the study
- Name(s) of the Site Investigators
- Explanation that the study involves research
- Explanation of the purpose of the study
- Explanation of the treatment procedures
- Description of randomization
- Description of the risks and benefits of participation in the study
- New findings that may affect willingness to maintain participation in the study
- A description of alternatives to participation in the study
- Explanation that all records will be kept confidential, but that records may be examined by representatives of the VA
- Who to contact for questions about the research and about subjects' rights
- Who to contact in the event of research-related injury
- A statement that participation in the study is voluntary and that a decision not to participate or to withdraw from the study after initially agreeing involves no penalty, loss of benefits or reduction in access to medical care
- The consequences of a subject's decision to withdraw from the study, and a description of the procedure for orderly termination of participation

In conjunction with the informed consent procedure, subjects will review and be asked to sign the Authorization for Release of Protected Health Information Form as required by the Health Insurance Portability and Privacy Act (HIPAA).

IX. Evaluation Procedures

A. Screening

The research study coordinator will be primarily responsible for identifying each non-emergent subject scheduled for a CABG-only procedure with planned SVG harvesting. A diagnostic catheterization must be performed within six months prior to the scheduled operation to be used as part of the baseline assessments. The participating surgeon(s) must agree that the subject is eligible for either study arm before randomization based on the inclusion/exclusion criteria defined by the protocol. This includes a review of the medical history for any lower extremity issues that would prevent the harvest of an effective SVG such as varicose veins, etc. Following subject informed consent, the baseline risk assessment, clinical data and subject self-reported symptom status and health related quality of life data will be obtained by the study team.

B. Randomization

All participating vein harvesters must meet the minimum EVH/OVH volume criteria [at least 100 EVH cases with low conversion rates (<5%) as part of an EVH program established for more than two years] to be eligible to enroll subjects in this study. Unless an urgent medical condition exists, the subject's surgery will be scheduled to occur at the earliest possible date based on expert harvester availability and other center circumstances. The randomization procedure will occur after the participating expert harvester is assigned to the subject. Subjects will be randomized within the assigned participating harvesters to one of the two study harvesting technique arms (EVH or OVH).

Recognizing that the subject's assignment to a participating vein harvester will have already occurred, the study randomization to either EVH or OVH will be done by a telephone call to the Perry Point Cooperative Studies Program Coordinating Center (CSPCC). A block randomization scheme will be used to randomize subjects in two treatment groups. A random sequence of block sizes will be used to reduce the chances of guessing future allocations.

C. Subject Assessments

Baseline assessments will be collected prior to surgery and randomization including Body Mass Index, Ankle-Brachial Index and Venous Clinical Severity Scale. Intraoperative assessments will be collected during the CABG procedure, 24 and 48 hours postoperatively as well as assessments at the time of hospital discharge or 30 days post-surgery, whichever occurs first. Subjects will return for a six-week clinic visit to assess the condition of their leg incision and healing status. Any necessary medical care required will be administered per the Institution's standard practice.

Subjects will receive a phone call every three months for follow-up for events until subject termination. Subjects will complete QoL surveys again at one year either by telephone or mail. If the subject reports a major adverse cardiac event (e.g. MI, repeat revascularization) after discharge from the hospital, then records will be obtained for final adjudication. Ascertainment for death will include review of VA central databases.

Following is a list of assessments/study details that we plan on collecting:

Screening Record: To compare subjects screened (but not enrolled) to subjects enrolled in this study, a comprehensive screening assessment will be completed for all potentially eligible subjects scheduled to receive an on-pump CABG-only procedure at a participating center by the study research coordinator.

Initial Assessment of Coronary Arteries: A cardiac catheterization will need to be performed within six months prior to randomization into the REGROUP trial. A local catheterization laboratory reading and a local clinical team assessment (of target vessels denoting those vessels planned to be bypassed) will be required. SYNTAX score will be calculated for each catheterization. In addition, the surgeon will be asked to describe a surgical plan indicating which bypass grafts are planned after reviewing the cardiac catheterization results.

Subject Risk Characteristics: To evaluate the impact of EVH vs. OVH procedures upon subject subgroups for a possible differential benefit, clinical data elements (i.e. height, weight, co-morbidity data, functional status, previous heart problems), cardiac catheterization and angiographic data, operative risk summary data (including operative death data), operative data, resource data (i.e. date and times of hospitalization, operation began and operation ended), socioeconomic data, and laboratory information will be collected. STS and VASQIP scores will be calculated by the site research coordinator with the oversight of the local site surgeon investigator.

Intraoperative Assessment: Details about vessels bypassed, including size and quality of conduits used, quality of target arteries and suture technique will be collected. Intraoperative complications will be recorded during the first 24 and 48 hours post-surgery and will include intraoperative bleeding complications, use of new intra-aortic balloon pump or assist device, unplanned cardiac arrest, blood product usage, as well as other variables.

Post-operative Lab and Diagnostic Test Evaluation: Twelve-lead EKGs will be obtained on the first two post-op days as noted in the detailed data forms. This isa routine test, commonly performed as part of usual postoperative clinical care for CABG patients. In the event the 12-lead EKG indicates either new pathologic Q waves or a new Left Bundle Branch Block, cardiac biomarker levels (preferably cardiac Troponin) will be collected from the subject every eight hours until a downward trend is seen. The peak cardiac biomarker value and reference range (including the 99th percentile of the Upper Reference Limit- URL) will be collected on the postoperative data collection Form 6.

30-Day Operative Mortality Assessment: Subjects will be noted to have died in-hospital or discharged alive. For subjects discharged alive, their vital status will be reassessed at 30 days post-CABG.

30-Day Morbidity Assessment: The presence/absence of major post-operative complications that occurred prior to discharge or within 30 days of CABG will be recorded. Leg wound healing assessments and infection data will also be collected at discharge.

In-hospital Resource Use: Several in-hospital indicators including operating room time, extubation time (with reintubation times noted), SICU length of stay, pre-operative and postoperative length of stay, and total blood product use (both intraoperatively and post-operatively) will be measured.

See Schedule of Events below:

Table 2. Schedule of Events

FORM	SCREEN	BASELINE (pre op) Visit 00	INTRA OP Visit 00	POST OP Visit 00	DC- 30 DAY Visit 01	6 WK Visit 02	3 MO Visit 03	6 MO Visit 06	9 MO Visit 09	12 Mo Visit 12	… Every 3 Mo	45 MO Visit 45	49 MO Visit 49	AS NEEDED
00 – Screening Log	X													
01 – Screening and Randomization	X													
02 – Baseline Information		X												
03 – Seattle Angina Questionnaire		X				X				X				
04 – VR-12		X				X				X				
05 – Intraoperative Data Collection			X											
06 – Post Operative Assessments				X										
07 – Discharge Assessments					X									
08 –Leg Incision Pain Questionnaire					X									
09 – Leg Incision Pain 6 week						X								
10 – Leg Incision Assessment					X*	X*								
11 – Mace Event (6 week)						X								
12 – Phone Call Follow-up							X	X	X	X	X	X	X	
13 – MACE Event Form													X	
14 - Termination														X
15 - SAE														X
16 – SAE Follow-up														X
17 – Harvester Experience														X
18 – Protocol Noncompliance														X
19 – Confirmation of MI by Local Site														X
20 – Confirmation of MI by Clinical Events Committee														X
21 – Cause of Death by Clinical Events Committee														X
86 - Consent	X													

* Form 10 is collected at two time points (discharge and 6 weeks), do not fax form until the six week assessment has been completed.

X. Post-Discharge Follow-up Assessments

A. Six Week Visit

Subjects will be assessed for leg wound complications approximately 6 weeks post-surgery during a clinic visit (this visit will take place between 4 weeks to 8 weeks post-surgery). Problems and/or procedures related to their cardiac health (i.e., acute myocardial infarctions, revascularization procedures, or a clinically indicated cardiac catheterization) will be obtained, Self-assessment satisfaction survey will be repeated at this time as well as Quality of Life questionnaires. Any necessary medical care required will be administered per the Institution's standard practice.

B. Interim Follow-Up

Every three months during the active follow-up period post-surgery, the site study coordinator will contact the study subjects by telephone to determine whether they have experienced any problems and/or procedures related to their cardiac health (i.e., acute myocardial infarctions, revascularization procedures, or a clinically indicated cardiac catheterization). These interim calls will collect MACE data, maintain rapport with subjects and let them know that the study team at the sites is interested in their progress. At one year the subject will complete QoL surveys by telephone or mail.

C. Monitoring Serious Adverse Events

a. Role of the Local Site Investigator in Reporting Serious Adverse Events (SAE)
The local site investigator is responsible for following CSP reporting requirements:

- Complying with the study procedure for reporting serious adverse events;
- Reviewing the accuracy and completeness of all SAEs reported; and
- Closely monitoring research subjects for any new SAEs.

b. Study Intervention
For the purpose of this study, the intervention for this study is defined as the *vein harvesting procedure for a saphenous vein graft in coronary artery bypass*.

c. Definition of a Serious Adverse Event (SAE)
A serious adverse event is an adverse event that results in one of the following outcomes:

- Result in death;
- Is life-threatening;
- Requires inpatient hospitalization or prolongation of existing hospitalization;
- Results in a persistent or significant incapacity or substantial disruption of the ability to conduct normal life functions;
- Important medical events that may not result in death, be life-threatening, or require hospitalization may be considered serious when, based upon appropriate medical judgment, they may jeopardize the subject and may require medical or surgical intervention to prevent one of the outcomes listed in this definition;

- SAEs will be reported regardless of their relationship to the study intervention.

d. SAE Monitoring and Reporting
Research subjects will be monitored at each study contact (i.e., phone call and follow-up clinic visits). Serious adverse events will be collected and recorded on the appropriate electronic case report form. Active monitoring of SAEs will begin as soon as a research subject signs the Informed Consent and will continue through end-of-study for each subject.

Serious adverse events require expedited reporting. Expedited reporting is defined as the completion and submission of the appropriate electronic case report form to the study's DataFax system within three (3) business days of the Local Site Investigator being aware of the SAE. The Study Pharmacist (or designee) is responsible for evaluating all SAEs for subject safety concerns by the close of business the next day after receipt of the SAE.

e. Reporting Serious Adverse Events to the Data Monitoring Committee (DMC)
The Perry Point CSP Center and CSP Clinical Research Pharmacy Coordinating Center will prepare aggregated SAE reports for the DMC annually or on a schedule set by the Committee.

D. Missed Visits

If the subject fails to come to the clinic for the six-week follow-up visit, the research coordinator at the site will call the subject on the same day or, at the latest, the next working day to inquire about the reason for the missed visit and to reschedule the subject's appointment as soon as possible. The research coordinator will continue trying to contact the subject until the subject receives an appointment or formally withdraws from the study. If the subject still refuses, the research coordinator will try to complete as much of the six-week assessments as possible on the phone by interview and/or by mail (e.g., the VR-12, Seattle Angina Questionnaire).

E. Termination

All subjects will be followed up actively until the sites are decommissioned. After the six-week clinic visit, each subject will be contacted by phone, every three months, to collect information on any MACE event, leg wound status, etc. If the subject cannot be contacted, his/her CPRS records will be assessed to check for any MACE event. If the subject dies or refuses to continue participation, he/she will be terminated. A termination form will be used to record the termination information for these subjects. At the end of the active follow-up phase (approximately 4.5 years from study start-up) each continuing subject's status will be recorded in a termination form to indicate official termination of the active follow-up phase of the study.

All subjects terminated from the active follow-up phase of the study will be continually followed by the study's national nurse coordinator where existing VA administrative databases will be mined for MACE for another two years (passive follow-up phase).

XI. Quality Control Procedures

A. Standardization/Validation of Measurements

Prior to the start of the study enrollment, all of the site investigators, harvesters and research coordinators will be provided with in-depth trainings on different aspects of the conduct of the study during a “kick-off” meeting to ensure proper understanding of the technical aspects of the protocol, to ensure uniformity in the completion and submission of the case report forms and to ensure uniformity in implementing and performing the study procedures.

During this meeting, the site investigators and harvesters will receive a half-day of Good Clinical Practices (GCP) training and the research coordinators will receive one day of GCP training. If any of the research coordinators from the participating sites is unable to attend the kick-off meeting, a repeat GCP training will be arranged. The GCP trainings will be provide by the SMART team from the Pharmacy Coordinating Center, Albuquerque, New Mexico. In any event, until this training is completed, the sites will not be allowed to begin randomization for the study.

Site investigators and research coordinators will also receive informed consent and study procedures training by the Perry Point Cooperative Studies Program staff during the kick-off meeting. The Perry Point Nurse Coordinator will provide a more detailed training on the study specific informed consent procedures. Other Perry Point Coordinating Center Staff will provide training on study procedures including the use of the study SharePoint portal, data collection on the DataFax platform, randomization and assessment schedules. Specific training sessions will be as follows:

- Study Procedures and Definitions
- EVH/OVH Best Practices
- Serious Adverse Event Reporting
- Strategies for subject contact and follow-up visits
- Data capture using DataFax

B. Subject Management

This research study will be conducted in full accordance with ethical principles of human research, including the provisions of the World Medical Association Declaration of Helsinki.
All CABG subjects will be screened for study eligibility using the same inclusion/exclusion criteria as defined in this protocol. Those subjects who qualify will be engaged in the study consent process by the clinical care team in collaboration with the research coordinator and led by the surgeon investigator. For those who agree to participate, local sites will adhere to their institution’s established best clinical practices in the care of the CABG subjects with exception to allowing for randomization of the harvesting technique at the time of surgery. Quality of life questionnaires will be completed at baseline, at six-week and at one- year post CABG. Subjects will be followed throughout their surgery and at discharge for research data collection including any serious adverse events (e.g. major adverse cardiac events). The subjects will be monitored the first and the second day post-operatively with 12-lead electrocardiograms . Per standard clinical care and most recent guidelines [Thyegesen 2012], cardiac biomarkers (preferably cardiac Troponin I or T) will be obtained if the 12-lead EKG demonstrates

evidence of type 5 [Thyegesen 2012] myocardial infarction, including new pathologic Q waves or new Left Bundle Branch Block. The cardiac biomarker will be obtained every eight hours until a downward trend is seen in the level. Usual inpatient post-operative care will follow institutional standards. Information on subject satisfaction and leg wound complications will be obtained at discharge and six weeks post-CABG. Local site coordinators will contact the subjects every three months to collect information by phone regarding the subject's health status throughout the active follow-up .

Subjects will be encouraged to seek medical attention as instructed upon discharge from the hospital. All efforts will be made to obtain follow-up information on subjects who have visited a hospital, underwent procedures or have been treated for serious adverse events in a non-study-related hospital(s). Non-study hospital related materials will be collected and reviewed at the study coordinating centers.

Throughout the duration of the study participation, the subject will be encouraged to maintain a point of contact with the local site investigator and the study coordinator for research related activities and questions. The study team at each site will maintain a dedicated point of contact for all subjects seeking information during their study participation. The study coordinator at each site will communicate any necessary medical information to the surgeon investigator and the clinical care team.

Long-term secondary MACE outcomes will be collected by passive follow-up using VA databases for an additional two years after the completion of the active follow-up phase.

C. Protocol Non-compliance

Any protocol non-compliance will be reported immediately to the Chairman's office and the Perry Point CSPCC. Each of these groups then reserves the right to forward notification, as required by local policy and regulation. Examples of protocol non-compliance include failure to obtain informed consent, failure to adhere to exclusion criteria, failure to report a serious adverse event, or no surgery is performed after randomization etc.

D. Site Performance Monitoring

In order to assure the successful conduct and completion of this study, all sites must adhere to certain performance standards. The Perry Point CSPCC and the Chairman's Office will jointly set performance standards and monitor site activities to assure that these standards are met.

All cooperative studies are on probation during the first year of enrollment. Studies that do not recruit at least 90% of the target enrollment during the first year are in danger of having monetary support stopped.

In order to meet the target enrollment, recruitment activities at the participating sites will be monitored aggressively. The Perry Point CSPCC will issue monthly recruitment reports to the Executive Committee and the Chairman's Office. The study Chair will contact the underperforming sites to identify problems and to recommend solutions.

XII. DATA MANAGEMENT AND CASE REPORT FORMS

A. Assessments, Case Report Forms (CRFs) and their Frequency of Administration and Collection

Please refer to Table 2 for a list of assessments and their frequencies of administration and collection.

B. Data Collection and Data Entry

Data management will be performed by the VA CSPCC Perry Point, MD using DataFax, a data management software. The CSPCC will have overall responsibility for the data at the end of the study.

All data will be collected at the study sites on source documents, which will be entered at the site into paper CRFs. The blank CRFs will be supplied by the VA CSPCC Perry Point, MD. CRFs are to be completed on an ongoing basis during the study. The medical chart and the source documents are the source of verification of data. CRFs should be completed according to the instructions in the study operations manual. The local site investigator is responsible for maintaining accurate, complete and up-to-date records for each subject. The local site investigator is also responsible for maintaining any source documentation related to the study, including any films, ECG tracings, computer discs or tapes.

Completed CRFs will be faxed by center personnel on a regular basis to the DataFax system at the VA CSPCC Perry Point, MD. DataFax allows the clinical centers to retain the original CRF and source documents while providing a faxed image to the VA CSPCC. Data within the faxed image are then checked for accuracy/completeness and entered into the study's database using DataFax software. Data received at the VA Perry Point CSPCC will be reviewed, verified and edited before being entered into the main study database. If incomplete or inaccurate data are found, a data clarification request will be forwarded to the clinical site for a response. Sites will resolve data errors before refaxing the corrected CRFs to the VA CSPCC. All corrections and changes to the data will be reviewed before being entered into the main study database. The VA CSP, Study Chair and the participating sites will receive reports at least monthly regarding the quality and quantity of data submitted to the VA Perry Point CSPCC.

Site investigators agree to routine data audits by the staff of the VA CSP monitoring unit, as well as by the CSPCC staff. The VA CSP monitors will routinely visit each site to assure that data submitted on the appropriate forms are in agreement with source documents at the sites. They will also verify that subject informed consent for study participation has been obtained and documented in the subject's progress notes, all essential documents required by GCP regulations are on file, and sites are conducting the study according to the research protocol. Any inconsistencies will be resolved, and any changes to the data forms will be made using established VA CSPCC Perry Point procedures.

When the study is completed and all data have been entered into the clinical database and the database has been checked for quality assurance and is locked, the CSPCC statisticians will perform statistical analyses of the data in accordance with the Statistical Analysis Plan (SAP). Periodically, during the study, CSPCC will prepare various summary reports of the data so that progress of the study can be monitored. These reports will be prepared for the Data Monitoring Committee (DMC) and other committees, as appropriate.

C. Study Documentation and Records Retention

Study documentation includes all paper CRFs, data clarification forms, source documents, monitoring logs and appointment schedules, investigator correspondence and regulatory documents (e.g., signed protocol and amendments, IRB correspondence and approved consent form and signed informed consent forms, Statement of Investigator form, etc.).

Source documents include all recordings of observations or notations of clinical activities and all reports and records necessary for the evaluation and reconstruction of the study. Thus, source documents include, but are not limited to laboratory reports, subject completed assessments, progress notes, hospital charts or pharmacy records and any other reports or records of any procedure performed in accordance with the protocol.

Whenever possible, the original recording of an observation should be retained as the source document; however, a photocopy is acceptable provided that it is a clear, legible, and exact duplication of the original document.

Research records for all study subjects including medical history and physical findings, laboratory data, and results of consultations with the primary care physician are to be maintained by the investigator in accordance with the VA record control schedule until notified by CSPCC. These records are to be maintained in compliance with IRB, State and Federal requirements, whichever is longest. It is the investigator's responsibility to retain copies of the completed CRFs until notified in writing by CSPCC that they can be destroyed. In all instances, the site must get permission from CSPCC prior to disposition of any study documentation and materials.

All records with identifiers will be stored indefinitely in accordance with the VA Records Control Schedule.

D. Data Security Plan

To maintain subject confidentiality, all data submitted to CSPCC for the current study will be coded using alpha-numeric identifiers only. Only on-site research staff and the sponsor's delegated program officials will have access to records that may identify subjects. Paper research and clinical records will be stored on site in a locked cabinet in a secure location. Electronic records will be accessible only by data management staff, clinical monitors and active site personnel who have furnished the required training and credentials. Permissions will be maintained by the CSPCC data management staff and can be revoked at any time. Subject information will not be released without written permission, except as necessary for monitoring by the FDA, the VA CSP monitoring unit or the Sponsor.

By participating in this protocol, the local site investigator agrees that within local regulatory restrictions and ethical considerations, the Sponsor or any regulatory agency may consult and/or copy study documents in order to verify data.

All data collected for this study will be handled and used in compliance with both the VA and the CSP data security plans. All subject level data will be treated as protected health information. Study personnel at CSPCC and at participating sites will be required to complete annual training courses. These courses will cover good clinical practices, human subjects' protection, cyber security, and privacy policy. Any data security breaches will be immediately reported. Subject level data will never be stored on portable storage devices unless it is encrypted, explicitly authorized, and use specific.

All private information will be kept on an encrypted, password protected server to which a small number of people will have access. Access to the cross-walk file linking the subject's identifiers and their study data will be restricted to the clinical site and to the approved personnel at the Chair's Office and Coordinating Center. This file will be destroyed according to CSP policy.

All data will be stored within the VA firewall and will be password protected at all times. Hard copy data will be sent via a traceable mail system (i.e., UPS), via a courier, or via secure fax. Access to these secure fax servers is restricted to the VA Perry Point coordinating center personnel with approved access to the system. All secure fax servers are compliant with VA directive 1605.1 and 6500. All data security incidents will be reported in accordance with VA policy within one hour of discovering the incident to:

- The District (local) Information Security Officer (ISO)
- The VA Perry Point CSPCC Data Security Officer
- The local IRB.

Administrative and healthcare utilization data on consenting participants will be extracted from the VA national database resources. The VA national data resources include, but are not limited to: the National Patient Care Database (OPC, PTF), VA-Medicare/Medicaid merge, national Laboratory and Pharmacy extracts (DSS, PBM), Corporate Data Warehouse (Health Data Repository), Medical Domain Web Services (MDWS), Patient Care Services Clinical Data Warehouse, Surgical Care Improvement Project (SQIP), Surgical Quality Workflow Manager (SQWM), VA Computerized Patient Record System (CPRS), Veteran Informatics and Computing Infrastructure (VINCI), VHA Support Service Center (VSSC), VA Surgery Quality Improvement Program (VASQIP), and the VA Vital Status File. Additionally, IT and Informatics initiatives to build the tools that will allow electronic medical record data extraction are being developed and will be used to obtain VISTA-level data to enhance the breadth of information and disease characterization that is lacking in the current national databases. This data will be downloaded/transferred with the appropriate permissions for use of these VA national databases, or individual VA Medical Centers, to the VA Central Research Database.

E. Data Sharing Plan

After the main results of this study have been published, de-identified data from this study may be shared with other VA investigators, other Federal health agencies, or academic institutions for the purpose of additional analyses provided this use has been approved by the appropriate VA oversight committee and there is an agreement in place that defines the limits of this use.

XIII. Feasibility of the study within the VA System

As seen in the sample size section, approximately 1,150 subjects will be required for this study. It is believed by the Planning Committee that 16 participating centers recruiting over a 3 year period could enroll this number of subjects. Each center would be required to recruit a minimum of 72 subjects over the three year recruitment period. This amounts to 24 subjects per year or two per month. This level of two subjects per month is what the Planning Committee decided was reasonable to expect for the sites. We have successfully identified 16 high CABG volume (>100 CABG/year) cardiac surgery centers in the VHA with prior experience in CSP studies and experience in EVH/OVH procedures who are willing to join our study. In addition, at least five centers can be available to replace any center that would not be able to participate.

From October 1, 2008 to September 30, 2009 (Fiscal Year 2009) 3,952 CABG-only procedures were performed in 40 cardiac surgery centers in the VHA. The average number of CABG-only procedures per site was 99/year or 8/month. Based on the REGROUP Trial inclusion/exclusion criteria, a screening log was maintained for a six month period (June to December 2011) at the Cardiac Surgery Program at the West Roxbury, Massachusetts VAMC: an average of 4 CABG subjects/month were found to be eligible for inclusion in the study or 50% of CABG procedures. Recent CSP CABG studies, such as CSP #474 (Radial) and CSP #517 (ROOBY), successfully randomized an average of 2 subjects per center per month while enrolling only isolated CABG subjects and excluding subjects with unsuitable coronary targets. We conclude that it is reasonable to assume that each of the 16 sites participating in CSP 588 will enroll two subjects/month. All 16 selected sites have performed at least 100 CABG/year for two consecutive years, contributing to an average of 1600 CABG/year in the 16 sites. To recruit the 384 subjects per year required for our study, 23% of this average number of CABG procedures will need to be entered into the study. Assuming conservatively that for the individual centers, the percentage of eligible subjects to recruit will be approximately 30-35% of all CABG procedures. Based on the limited inclusion/exclusion criteria, the sample size goal is perceived by the Planning Committee to be achievable.

XIV. Requirements for Participating Centers

All participating centers must be able and willing to adhere to the study protocol. The minimum requirements for participating medical centers include:

- Site Principal Investigator: Each center must identify their site's principal surgeon investigator who enthusiastically supports the study and is willing to devote sufficient time and energy to ensure that the study's goals are met. For VA medical centers, the site surgeon investigator must have at least a 5/8^{ths} VA appointment for receiving VA research funds.

- EVH/OVH Harvester: Each center/study surgeon investigator must identify their site's harvester(s). The harvester(s) must have performed a minimum of 100 EVH procedures with a conversion rate to OVH < 5%to qualify for participation in the REGROUP trial. Each harvester must also demonstrate competency in OVH or the study surgeon investigator directly attending the case must perform the OVH in the same manner as provided during the course of usual care

absent a clinical trial. A subcommittee of the Executive Committee will convene and review each site's harvester qualifications and issue guidance/recommendations for sites as needed.

- Enrollment Volume: Each center must provide documentation that it will be able to recruit 24 subjects receiving a CABG-only procedure per year into the study who meet all inclusion/exclusion criteria. This total will be 72 subjects receiving CABG-only procedures over the three-year recruitment period.

- Administrative Support: Each center must provide assurance by the Chief of Surgery Service and/or the Chief of Staff that their site investigator will receive full administrative support.

- Multiple Participating Surgeons: At each center, there should be at least one (and preferably two or more) cardiothoracic surgery attending faculty team members participating that agree to randomize and operate on all consenting, eligible subjects with a planned CABG-only on-pump procedure using a median sternotomy incision. Although participating surgeons may enter/leave the study (after approval by the Chairman's office and Perry Point CSP Coordinating Center), the center must make every effort to recruit and to retain at least one qualified participating surgeon to enroll subjects in this study.

- Local Approvals and Reporting Required: Acceptance and approval of the protocol and the informed consent document with only minor changes by the site investigator, the local VA medical center's R&D Committee, and the local IRB. Copies of the meeting minutes indicating approval by the local R&D Committee must be submitted to the Perry Point CSP Coordinating Center prior to enrolling subjects at the local center.

- Global Monitoring and Reporting Responsibilities Delegated: By agreeing to participate in the study, centers delegate responsibility for global monitoring of the ongoing study to the Data Monitoring Committee, the Cooperative Studies Scientific Evaluation Committee (CSSEC), the Institutional Review Board, and the Perry Point CSP Coordinating Center. However, the local Research and Development Committee and the local IRB of the center will require the site investigator to submit annual reports concerning the status of the study for local monitoring purposes.

- Research Study Coordinator: The site investigator must make every effort to recruit and retain an enthusiastic research study coordinator, preferably one experienced in clinical trials, who will work diligently with the site investigator to meet the study's goals. Moreover, this local study coordinator must work collaboratively with the Chairman's office staff, including the National Nurse Coordinator.

XV. Study Organization Administration and Monitoring

A. Monitoring Bodies

The groups charged with monitoring the various aspects of the study will be the Executive Committee, the Data Monitoring Committee (DMC), and the local site IRBs. These committees will meet at regular intervals according to the current Cooperative Studies Program guidelines: prior to the beginning of subject enrollment and at least every twelve months thereafter. In addition, the CSP Site Monitoring, Auditing and Review Team (SMART), located at the CSP Clinical Research Pharmacy Coordinating Center (CSPCRPCC), will monitor the trial for GCP compliance.

The Executive Committee is the management and decision-making body for the operational aspects of the study and will monitor the performance of participating medical centers and the quality of data collected. The Executive Committee will formulate publication plans and will oversee the publication and presentation of all data from the study. The Committee must grant permission before any study data may be used for presentation or publication.

The Data Monitoring Committee (DMC) will review the progress of the study and will monitor subject intake, outcomes, serious adverse events, and other issues related to subject safety. The DMC makes recommendations to the Director of the Clinical Science Research and Development (CSRD) Service about whether the study should continue or be stopped. The DMC will consist of experts in the fields of Endoscopic Vein Harvesting, Cardiothoracic Surgery, Cardiology, clinical trials, biostatistics, and ethics. These experts will not be participants in the trial and will not have participated in the planning of the protocol. The DMC will consider safety or other circumstances as grounds for early termination, including either compelling internal or external evidence of treatment differences or the unfeasibility of addressing the study hypothesis (e.g., poor subject enrollment, poor adherence to protocol).

At each of its meetings during the study period, the DMC will review the randomization rates and assess the difference between the actual and the projected rates, as well as the impact of these assessments on overall trial size. An assessment of whether the trial should be continued will be made followed by recommendations, as appropriate. All serious adverse events will be reported on a regular basis to the DMC for their review. Unexpected serious adverse events may be reported to the DMC in an expedited manner based upon the consensus of the Study Chairman, the Study Biostatistician, and the Perry Point CSPCC Director. The Study Biostatistician will provide the appropriate data to the DMC at specified intervals for this purpose.

The Clinical Events Committee (CEC) will consist of one cardiologist and two cardiac surgeons who will meet semi-annually (either in person or via conference calls). This committee will systematically evaluate all study deaths for cardiac versus non-cardiac causes. The national nurse coordinator will prepare a Clinical Events packet for each subject death which will consist of notes, labs, tests from the subject's medical record and death certificates or autopsy reports when available. In addition, this committee will evaluate and confirm all reported myocardial infarctions (MI) using the published AHA/ACC/ECS Universal criteria (Thygesen 2007). This may be accomplished using notes, labs and diagnostic tests from the subject's medical record. To assure compliance with data security

procedures, all necessary documentation will be “scrubbed” to remove subject identifiers and any reference to treatment arm received.

The local sites IRB will be the study’s primary IRB and the IRB of record for the study. It will be responsible for the initial and continuing IRB reviews of the study. The local site IRB must review and approve amendments (changes to inclusion/exclusion criteria, protocols, informed consents, etc.), deviations, and review reports about serious adverse events and problems, complaints, terminations, etc. and that the investigators must provide the local site IRB all supporting documentation. The CSPCC will be responsible for providing the local site IRB with all materials that are required for each review and to respond to local IRB’s queries and requests for additional materials. The local site IRB approves the original informed consent template and any requested changes to the informed consent forms.

The Human Rights Committee (HRC) at the Coordinating Center will review the study prior to its initiation to the local site IRB to ensure proper protection of the subjects’ rights and safety. The CSPCC HRC will also conduct at least one site visit during the study to interview study subjects to assess whether subject rights are being fully protected.

CSP SMART will provide GCP training at the kick-off meeting and will conduct initiation visits at all sites. It also will conduct a GCP site review and a for cause audit of a participating site if requested by any of the monitoring bodies. At a minimum, each site will be visited at least once during the study by SMART. The local site IRB will receive a copy of all SMART monitoring reports.

The Quality Assurance Section at the Perry Point CSPCC will provide central monitoring of study sites to ensure compliance with Good Clinical Practice. Monitoring may include but is not limited to the informed consent process, data validation, source verification, and safety reporting. Additional site-specific monitoring may be conducted if triggered by poor study performance. Site performance findings may result in on-site visits by the CSPCC QA Nurse Specialist or other CSPCC central monitoring personnel to evaluate the need for additional site training to remedy compliance concerns.

The Study Group, which consists of all site investigators, participating harvesters, and research coordinators, will meet annually to discuss the progress of the study and any problems encountered during the conduct of the trial.

B. Monitoring Subject Safety

The Perry Point CSPCC and CSPCRPCC will provide summaries of all serious adverse events reported to the Study Chairman and DMC at least twice a year.

C. Monitoring Subject Intake and Probation or Termination of Participating Sites

The Study Chairman and the Study Biostatistician will monitor the intake rate and operational aspects of the study. Participating medical centers will continue in the study only if adequate subject intake is maintained. The Executive Committee may take action leading to the discontinuation of subject enrollment at a center with the concurrence of the CSPCC Director. If recruitment is not proceeding at an appropriate rate, the Study Chairman and Study Biostatistician will

scrutinize the reasons for inadequate subject participation. Based on this information, the Executive Committee may choose to drop centers or add additional centers. The DMC and Director of CSRD will be notified regarding the dropping or adding of centers. Participating sites that do not enroll at least 24 subjects during the first 12 months of the study will be placed on probation and given an opportunity to improve within a reasonable period. After the first 12 months, participating sites that do not reach 75% of enrollment target during any six-month evaluation time will be placed on probation and given an opportunity to improve within a reasonable period. If a medical center is placed on probation, the Study Chairman will confer with the site personnel and visit the site, if necessary, to help improve the rate of recruitment. If there is no improvement in accrual during the probation period, the site may be subject to reduced funding or possible termination as a study site. To plan for the possible termination of a site(s) and the addition of a new site(s), back up sites with IRB approval will be identified prior to study initiation to minimize the delay in adding a new site. The Executive Committee will only take actions leading to discontinuation of a center with the concurrence of the CSPCC Director. If a center is terminated from the trial, resources will be reallocated to other centers or used to start up a backup site.

D. Alternate Plan if Recruitment Goals are not Met

After the study has been in the recruitment phase for three months, the Study Chairman and the National Nurse Coordinator will contact the local site investigators and research coordinators to identify any common obstacles to subject recruitment and identify steps that might be taken to reduce those obstacles. If recruitment has fallen short of anticipated goals by month six due to low CABG volume at the participating centers, the Study Chairman will make a proposal to the Executive Committee to alter the inclusion criteria from CABG only on-pump procedures to additionally include on-pump CABG plus valve procedures.

E. Monitoring Medical Center Performance

Each participating site will be monitored for data quality, completeness of follow-up and adherence to the protocol. Regularly scheduled conference calls (at least monthly) with the sites, CSPCC and Chairman's office will be held to address data collection, protocol procedures and other issues. Strict adherence to the protocol will be expected of every participating center and will be monitored by the DMC, the Executive Committee, and the CSPCC. Documentation of protocol noncompliance will be required and any medical center with repeated protocol noncompliance issues will be recommended to the Executive Committee for termination. If a participating site investigator feels that adherence to the protocol will in any way be detrimental to a particular subject's health or well-being, the interest of the subject will take precedence over continued study participation. In addition, CSPCC, the Executive Committee and the DMC will monitor protocol adherence centrally. The Executive Committee will consider recommending a full GCP audit to be conducted by SMART for any site with repeated protocol noncompliance issues and will consider terminating the site from the trial.

Data quality and completeness of data retrieval will be closely monitored on an ongoing basis by the Coordinating center. The study biostatistician will present interim monitoring reports to the Executive Committee and the DMC that will include the following types of information:

- Subject intake
- Randomization
- Breaches of protocol
- Adherence and compliance of study protocol
- Missed study visits
- Completeness of follow-up
- Audit and site visit results

If a site is identified as an outlier in terms of data quality, a site conference call or site visit will be initiated to assess the reasons why problems are occurring and how they can be corrected. If the problems continue, the site may be placed on probation or terminated from the study.

F. **Monitoring of safety, efficacy and futility**

As previously noted, the DMC will review the accumulating data and be responsible for determining whether or not to recommend that the trial be stopped for efficacy, futility or safety. Data summaries will be prepared for the DMC for these purposes. Frequent summaries of serious adverse events will be prepared for the DMC for monitoring of safety, i.e., at least twice a year. To aid the DMC in their deliberations, other relevant information specific to CSP# 588 will be made available. Complete details of the interim monitoring plans for the study are given in Section XVII Biostatistical Considerations below.

XVI. Good Clinical Practices

A. Role of GCP

This trial will be conducted in compliance with Good Clinical Practice (GCP) regulations. The intent of these regulations is to safeguard subjects' welfare and assure the validity of data resulting from the clinical research. The VA Cooperative Studies Program will assist Local Site Investigators (LSIs) in complying with GCP requirements through its Site Monitoring, Auditing and Resource Team (SMART) based in Albuquerque, NM. SMART serves as the Quality Assurance arm of CSP for GCP compliance. Study site personnel will receive GCP training at the study organizational meeting. SMART will provide training, manuals and materials to assist study personnel in organizing study files and will be available throughout the trial to advise and assist LSIs regarding GCP issues.

B. Summary of Monitoring and Auditing Plans

a. Monitoring Visits
   (1) Initiation visits at each site soon after study start-up
   (2) Additional monitoring visits may be conducted as deemed necessary by study leadership or SMART.

b. Audits
   (1) Routine audits – independent site visits to one or more sites per year as determined by SMART.

(2) For-Cause audits –independent audit of a site as requested by study leadership or CSP Central Office.
(3) Audits may be scheduled or unannounced.

XVII. Biostatistical Considerations

A. Expected Treatment Effects

In the ROOBY study (CSP # 517) (Shroyer 2009), one year MACE rates were 9.9% in the OVH group and 15.3% in the EVH group (p=0.0025). The executive committee for the REGROUP study assumes that during the REGROUP study, 15.5% of the subjects in the EVH group will experience MACE in the first year post surgery. The committee also expects a 6 percentage point improvement in the one-year MACE rate in the OVH group.

B. Sample size calculation for the REGROUP study

To detect the expected 6 percentage point difference in one-year MACE rates between EVH (15.5%) and OVH (9.5%), a sample size of **545 in each group** will be required at 85% power, 5% type-I error rate and with a two-sided test. A sensitivity analysis was also done with various scenarios which are shown in the Table 3. Since, it would be possible to capture the majority of the MACE from the VA databases even if the subjects drop out before the one-year clinic visit, a relatively small inflation factor of 5% is used to inflate the sample size to account for the drop-outs. Approximately, 1150 subjects need to be randomized in the study to achieve the said power.

	Case 1	Case 2	Case 3	Case 4	Case 5	Case 6	Case 7	Case 8	Case 9
Tails	2	2	2	2	**2**	2	2	2	2
Proportion 1 (%)	9.9	9.9	9.9	9.5	**9.5**	9.5	10	10	10
Proportion 2 (%)	15.3	15.3	15.3	15.5	**15.5**	15.5	16	16	16
Alpha	0.05	0.05	0.05	0.05	**0.05**	0.05	0.05	0.05	0.05
Power (%)	90	85	80	90	**85**	80	90	85	80
Allocation ratio	1:1	1:1	1:1	1:1	**1:1**	1:1	1:1	1:1	1:1
Sample Size	792	677	592	637	**545**	476	659	563	492
Total Sample Size	1584	1354	1184	1274	**1090**	952	1318	1126	984
**Attrition = 5%**	**1667**	**1425**	**1246**	**1341**	**1147**	**1002**	**1387**	**1185**	**1036**
Attrition = 10%	1760	1504	1316	1416	**1211**	1058	1464	1251	1093
Attrition = 15%	1864	1593	1393	1499	**1282**	1120	1551	1325	1158

Table 3: Various sample size scenarios for the REGROUP study

Based on the one-year MACE rate difference between EVH and OVH groups as obtained from CSP 517, if we assume at the end of the active follow-up period (approximately 4.5 years from the beginning of the recruitment process) difference in survival rates (i.e., the proportion of subjects who will not experience

MACE) between the two harvesting groups will be approximately 8 percentage point (i.e., 83.5% in the EVH and 91.5% in OVH), a sample size of 532 per group will be required to achieve a power of 97.5%. So, the sample size of 1150 (with approximately 7% attrition rate accounted for) will provide adequate power to the time-to-MACE event primary outcome.

C. Duration of Study/Number of Participating Sites

Based on the experience of previous CSP studies on CABG performed in the last decade (2000-2010) (e.g. CSP #517 ROOBY and CSP #474 RADIAL), a two subject/month/site enrollment rate was assumed to be a reasonable enrollment rate for this study. With this assumption, various scenarios were created to find an optimum balance between the number of sites, the study duration (which includes enrollment period and follow-up period) and the estimated budget. The chosen scenario was with 16 sites around the country where both EVH and OVH are currently practiced. With 16 sites and two subjects/month/site rate of enrollment, the enrollment period was found to be approximately, three years which would be followed by a minimum of one-year follow-up for the last subject randomized into the study. This will allow approximately 4 – 4.5 years of active follow-up of all randomized subjects followed by additional 2 years of passive follow-up using the VA databases. The total duration for the 16 participating sites will be approximately 6.5 years.

D. Statistical Analysis Plan

1. Intent-to-treat (ITT) analysis

Intent-to-treat population is defined as the population of subjects who will be randomized to either of the harvesting technique groups – EVH or OVH. The subjects will be categorized (in terms of their harvesting technique group assignment) based on their initial randomized group irrespective of conversion before surgery and will be included in analyses irrespective of their status – completer or drop out of the study before completion. Analyses of all outcome measures – primary and secondary – will use ITT population.

All statistical tests will be 2-sided and the primary MACE outcome will be tested at 5% level of significance. SAS will be used to conduct all the statistical analyses.

2. Primary Outcome Measure

MACE, the composite endpoint that includes Death (all cause), Myocardial Infarction (MI) and Revascularization for myocardial ischemia, will be the primary outcome measure after randomization and the index CABG. Each randomized subject (either in Endoscopic or in Open harvesting group) will be followed after the index CABG to capture the time-to-MACE event where an ‘EVENT’ will be defined as either death (all cause) or an MI or a revascularization procedure during the follow-up period. Total follow-up period will be 6.5 years of which the first 4.5 years will be active follow-up (using in-clinic visit or by telephone) period and will be carried out by the site personnel. The remaining 2 years will be passive follow-up which will be carried out centrally by the chair’s office staff using the VA patient database. A minimum of 1 year of active follow-up will be used for subjects who will be randomized at the tail end of the 3-year projected enrollment period of the study. The subjects

who will either be lost to follow-up or will not experience an 'EVENT' before the end of the follow-up period will be considered as right censored.

The primary analysis of such time-to-Mace event data will include the events only from the active follow-up period (which is 4.5 years). The secondary analysis will include events from the entire 6.5 years of follow-up period.

Survival analysis techniques will be used to analyze the time-to-MACE event data for both primary and secondary analyses.

Kaplan-Meier analysis, a nonparametric method, will be used to estimate the survival (not experiencing MACE) over time in the two harvesting groups and a log-rank statistic will be used to test the equality of the survival function estimates in the two groups (the null hypothesis).

Cox's Proportional Hazards models will be used to investigate the effect of harvesting technique on the time until MACE adjusted for other potential influential variables, such as, age, gender, harvester's experience etc.

3. Secondary Outcomes Measures

One and Three-year MACE: This composite endpoint consists of

i. Death (all cause);
ii. Myocardial Infarction and
iii. Revascularization for myocardial ischemia.

Subjects who suffer any one of these three outcomes within the first year or in the first three years after the index CABG will be counted towards calculating the proportions of subjects with MACE (yes/no) in each harvesting technique group – subjects with endoscopic vein grafting or subjects with open vein grafting. Only the first event will be considered as the MACE event. These two proportions will be compared using a chi-square test.

<u>Post-operative Leg Wound Complications</u>: All subjects in both harvesting technique groups – EVH or OVH – will be examined for complications of the leg wound from harvesting at discharge and at six weeks post-surgery. Post-operative leg wound complication status (yes/no) will be recorded at discharge and at six weeks post-surgery. Proportion of subjects with leg wound complications will be computed for each treatment group and these proportions at each time point will be compared using chi-square tests. The impact of confounding variables, such as, BMI, diabetes status, smoking status, on the post-operative leg wound infection will be analyzed using a logistic regression.

<u>Severity of Incisional Leg Pain</u>: Severe leg pain, due to incisions made during vein grafting, data will be collected at discharge and at six-week post CABG. Proportion of subjects with severe pain (pain score 3 and above = yes) at each time point will be compared using chi-square statistics.

<u>Quality of Life</u>: QoL scores using VR-12 and Seattle Angina Questionnaire will be computed at baseline, six week, and 12 months post-surgery for the subjects in the two harvesting technique groups (EVH and OVH). Subjects in both groups will be categorized as "improved", "no change" and "worse" based on their baseline scores. The proportion of subjects in these three categories will be compared between the two groups using chi-square statistics. The actual scores from these measures will also be used to compare subjects in the two harvesting technique groups using analyses of covariance techniques, where the baseline (pre-surgery) scores will be used as a covariate.

E. Interim Monitoring

An independent oversight committee, a Data Monitoring Committee (DMC), will be monitoring study progress at predetermined time points over the entire duration of the study. The committee will receive analyses of the primary outcome measures and the important secondary outcome measures on a routine basis. In general, this committee meets at six to nine months after the start of subject recruitment and yearly thereafter. So, in total, this committee will meet maximum four times during the four years of the study duration. The committee will receive reports about three weeks prior to their annual meetings and at six monthly intervals in between the annual meetings. Since the primary outcome measure (MACE) are times-to-MACE event, sufficient data for DMC's first review will not be available until the study has been ongoing for at least 2 years. So there will be approximately 8 interim analyses of the primary outcome measure based on which the DMC will decide on study's continuation.

F. Criteria for Study Termination

When repeated significance tests are performed on accumulating data as part of a routine monitoring function, the overall type-I error rate is inflated and the probability of a false positive finding is also increased. A number of methods have been developed to provide guidance on study termination rules based on multiple looks on the primary outcome measures for the review committees while keeping the overall type-I error rate maintained at 5%. For example, Haybittle-Peto or Lan-DeMets group sequential boundaries will provide study stopping guidance/criteria for the DMCs to implement. An example of typical Lan-DeMetes boundary for six looks is illustrated in Figure 2. The DMC will make the final decision on the type of stopping rule that will be used for the REGROUP trial.

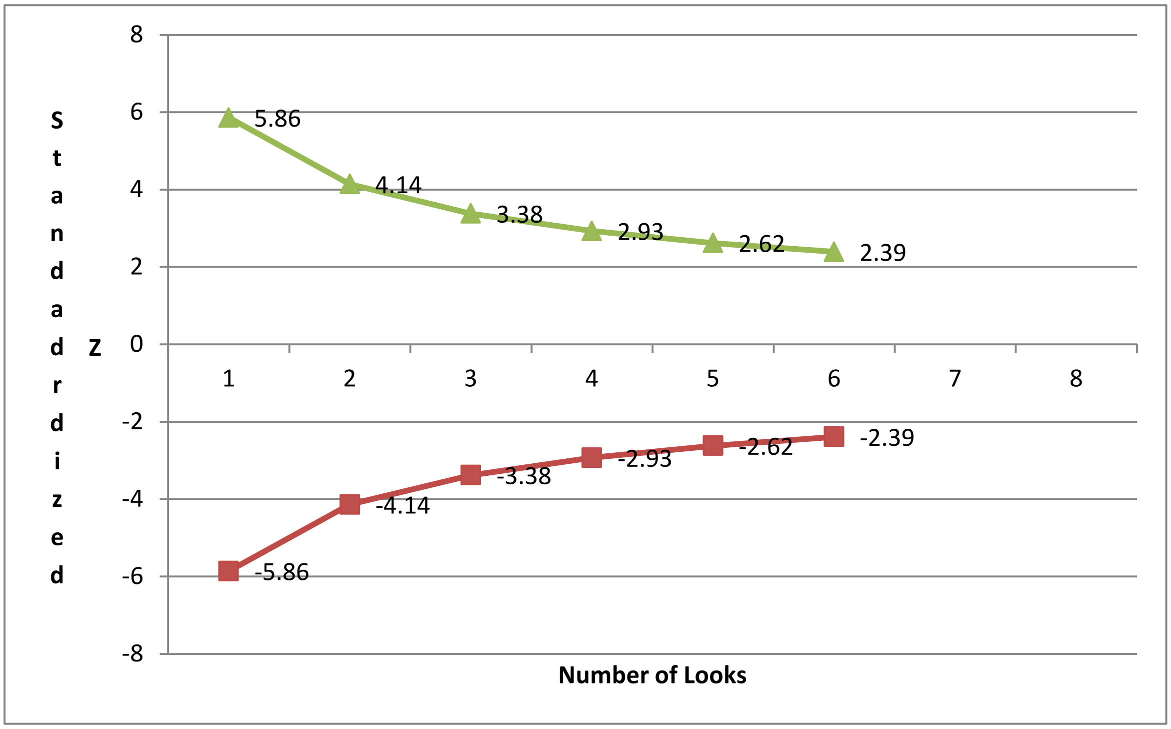

Figure 2: Lan-DeMets group sequential boundary for eight interim looks

This group sequential boundary is used as a guide for study termination at any interim look. If the z-statistic at any interim look falls outside either the lower or the upper boundary, then the study should be considered for termination.

G. Handling of Missing Data

Every effort will be made to minimize the occurrence of missing data, particularly for the primary and main secondary outcome measures. For the primary outcome (MACE), every effort will be made to contact the subjects over the phone every three months until subject termination. In the event of a potential drop out, every effort will be made to capture the MACE data from the VA databases.

H. Reporting of Any Deviations from the Original Statistical Plan

A more detailed statistical analysis plan (SAP) will be generated which will include the details of each statistical analysis plan for each outcome measure along with the suggested table shells for any reports that will be produced during the study and at the end of the study. Any deviations in the statistical plan from the protocol will be specified in the SAP. Any deviations from the SAP will be specified in the main manuscript which will be prepared and published at the end of the study.

XVIII. PUBLICATIONS

A. Publication Policy

It is the policy of the Cooperative Studies Program not to reveal outcome data to site investigators until the data collection phase of the study is complete. This policy is meant to prevent possible biases that might affect data collection. Members of the DMC will be reviewing outcome results to ensure that the study will be terminated early if a treatment is identified as prohibitively dangerous or if a definitive answer is reached prior to the scheduled study termination date.

All presentations and publications resulting from this study will follow CSP policy as specified by the CSP guidelines. The presentation or publication of any or all data collected by site investigators on subjects entered into a Department of Veterans Affairs Cooperative Study is under the direct control of the study's Executive Committee. No individual site investigator has the right to use the study's data to perform analyses or interpretations, or to make public presentations or seek publication of any or all of the data without specific approval of the Executive Committee.

The Executive Committee has the authority to establish any number of publication committees, which usually will comprise of subgroups of site investigators and some members of the Executive Committee, for the purpose of producing manuscripts for presentation and publication. Any presentation or publication related to this study should be circulated to the Executive Committee for review, comments and suggestions at least four weeks prior to submission of the manuscript to the presenting or publishing body.

All publications must give proper recognition to the funding source and should list all study participating site personnel (not necessarily as authors of the manuscript). If an investigator's major salary support and/or commitment is from the VA, it is obligatory that the investigator lists the VA as his/her primary institutional affiliation. Submission of manuscripts or abstracts must follow the usual VA policy; ideally, a subtitle states, "A Department of Veterans Affairs Cooperative Study." The CSP also requires that every manuscript be reviewed and approved by the CSPCC Director prior to submission as a final quality control step. Mechanisms for appeal by an investigator will follow procedures defined by the VA Office of Research and Development.

Participation in a Department of Veterans Affairs Cooperative Studies Program clinical trial is voluntary. Any investigator who cannot accept these operational guidelines regarding publication policy should not volunteer to participate in the study.

B. Planned Publications

Primary publication: Upon completion of the study, a manuscript will be prepared that focuses on the primary outcome, i.e. endoscopic saphenous vein harvesting vs. open saphenous vein harvesting MACE rates during the follow-up period.

Other publications: Other planned publications will include at least the following: Quality of Life with EVH and OVH , leg wound complications, 1 and 3 year MACE.

XIX. REFERENCES

[Alexander 2005] Alexander JH, Hafley G, Harrington RA, PREVENT-IV Investigators. Efficacy and safety of edifoligide, an E2F transcription factor decoy, for prevention of vein graft failure following coronary artery bypass graft surgery: PREVENT-IV: a randomized controlled trial. JAMA 2005;294:2446-54.

[Allen 2005] Allen K, Cheng D, Cohn W, Connolly M, Edgerton J, Falk V, et al. Endoscopic vascular harvest in coronary artery bypass grafting surgery: A consensus statement of the International Society of Minimally Invasive Cardiothoracic Surgery (ISMICS) 2005. *Innovations* 2005;1(2):51-60.

[Andreasen 2008] Andreasen JJ, Nekrasas V, Dethlefsen C. Endoscopic vs. open saphenous vein harvest for coronary artery bypass grafting: a prospective randomized trial. *Eur J Cardiothorac Surg* 2008; 34(2):384-9.

[Aranki 2009] Aranki SF, Shopnick B. Endoscopic versus open vein-graft harvesting. *N Engl J Med* 2009; 361(19):1907

[Barnard 2011] Barnard JB, Keenan DJM. Endoscopic saphenous vein harvesting for coronary artery bypass grafts: NICE guidance. *Heart* 2011;97:327-329.

[Barner 2008] Barner HB. Operative treatment of coronary atherosclerosis. *Ann Thorac Surg* 2008;85:1473-82

[Barner 1990] Barner HB, Fisher VW. Endothelial preservation in human saphenous veins harvested for coronary grafting. *J Thorac Cardiovasc Surg* 1990;100:148-149.

[Brown 2007] Brown EN, Burris NS, Gu J, et al. Thinking inside the graft: applications of optical coherence tomography in coronary artery bypass grafting. *J Biomed Opt* 2007;12(5):051704

[Brown 2007] Brown EN, Kon ZN, Tran R, Burris NS, Gu J, Laird P, et al. Strategies to reduce intraluminal clot formation in endoscopically harvested saphenous veins. *J Thorac Cardiovasc Surg* 2007;134(5):1259-65.

[Brown 2010] Brown ML, Sundt TM. Surgical coronary artery revascularization. In: Yusuf S, Cairns JA, Camm AJ, Fallen EL, Gersh BJ. Evidence-Based Cardiology. 3rd Edition 2010, Wiley-Blackwell, Hoboken, NJ, p.379-391.

[Bush 2002] Bush HL, Jakuboski JA, Curl GR, et al. The natural history of endothelial structure and function in arterialized vein grafts. *J Vasc Surg* 1986;3:204-215

[Bitondo 2002] Bitondo JM, Dagget WM, Torchiana DF, et al. Endoscopic versus open saphenous vein harvest: a comparison of postoperative wound complications. *Ann Thorac Surg* 2002; 73:523-528
[Buxton 2009] Buxton BF, Hayward PAR, Newcomb AE, et al. Choice of conduits for coronary artery bypass grafting: craft or science? *Eur J Cardiothorac Surg* 2009;35:658-670.

[Cadwallader 2009] Cadwallader RA, Walsh SR, Cooper DG, Tang TY, Sadat U, Biyke JR. Great saphenous vein harvesting: a systematic review and meta-analysis of open versus endoscopic techniques. *Vasc Endovasc Surg* 2009;43(6):561-6.

[Campeau 1983] Campeau L, Enjalbert M, Lesperance J, et al. Atherosclerosis and late closure of aortocoronary saphenous vein grafts: sequential angiographic studies at 2 weeks, 1 year, 5to 7 years and 10 to 12 years after surgery. Circulation 1983;68:SII-1

[Campeau 1984] Campeau L, Enjalbert M, Lesperance J, et al. The relation of risk factors to the development of atherosclerosis in saphenous vein bypass grafts and the progression of disease in the native circulation: a study 10 years after aortocoronary bypass surgery. *N Engl J Med* 1984;311:1329-32.

[Carpino 2000] Carpino PA, Khabbaz KR, Bojar RM, Rastegar H, Warner KG, Murphy RE, Payne DD. Clinical benefits of endoscopic vein harvesting in patients with risk factors for saphenectomy wound infections undergoing coronary artery bypass grafting. *J Thorac Cardiovasc Surg* 2000;119(1):69-75.

[Cheng 2010] Cheng DCH, Martin J, Ferdinand FD, Puskas JD, Diegler A, Allen KB. Endoscopic vein-graft harvesting: balancing the risk and benefit. *Innovations* 2010;5:70-73.

[Connolly 2009] Connolly MW, Poston RS. Endoscopic versus open vein-graft harvesting. *N Engl J Med* 2009; 361(19):1907-8

[Cox 1991] Cox JL, Chiasson DA, Gottlieb AI. Stranger in a strange land: the pathogenesis of saphenous vein graft stenosis with emphasis on structural and functional differences between veins and arteries. *Progr Cardiovasc Dis* 1991;34:45-68

[Dao 2012] Dao K, Malgor RD, Montecalvo J, Hines G. The current status of endoscopic vein harvest in cardiac and peripheral vascular surgery. *Cardiology in Review* 2012 ePub ahead of print DOI: 10.1097

[Deppe 2013] Deppe AC, Liakopuolos OJ, Choi YH, et al. Endoscopic vein harvesting for coronary artery bypass grafting: a systematic review with meta-analysis of 27,789 patients. *J Surg Res* 2013;180:114-124.

[Desai 2011] Desai P, Kiani S, Thiruvanthan N, et al. Impact of the learning curve for endoscopic vein harvest on conduit quality and early graft patency. *Ann Thorac Surg* 2011;91:1385-92

[FitzGibbon 1978] FitzGibbon GM, Burton JR, Leach AJ. Coronary bypass graft fate: angiographic grading of 1400 consecutive grafts early after operation and of 1132 after one year. *Circulation* 1978;57:1070-4

[FitzGibbon 1986] FitzGibbon GM, Leach AJ, Keon WJ, et al. Coronary bypass graft fate. Angiographic study of 1,179 vein grafts early, one year and five years after operation. *J Thorac Cardiovasc Surg* 1986;91:773-8

[FitzGibbon 1996] FitzGibbon GM, Kafka HP, Leach AJ, Keon WJ, Hooper GD, Burton JR. Coronary bypass graft fate and patient outcome: angiographic follow-up of 5,065 grafts related to survival and reoperation in 1,388 patients during 25 years. J Am Coll Cardiol 1996;28:616-26.
[Goldman 2011] Goldman S, Sethi GK, Holman W, et al. Radial artery grafts vs. saphenous vein grafts in coronary artery bypass surgery: A randomized trial. *JAMA* 2011;305(2):167-174 [VA CSP #474 main manuscript]

[Goldman 1997] Goldman S, Zadina K, Krasnicka B, et al. Predictors of graft patency 3 years after coronary artery bypass surgery. *J Am Coll Cardiol* 1997;29:1563-1568 [VA CSP #297 main manuscript]

[Goldman 1989] Goldman S, Copeland J, Moritz T, et al. Saphenous vein graft patency one year after coronary artery bypass graft surgery: Effect of antiplatelet therapy. *Circulation* 1989;80:1190-7

[Gundry 1980] Gundry SR, Jones M, Ishihara T, et al. Optimal preparation techniques for human saphenous vein grafts. *Surgery* 1980;88:785-794.

[Halabi 2005] Halabi AR, Alexander JH, Shaw LK, et al. Relation of early saphenous vein graft failure to outcomes following coronary artery bypass surgery. *Am J Cardiol* 2005;96:1254-1259.

[Hayward 2008] Hayward PA et al. Effect of radial artery or saphenous vein conduit for the second graft on 6-year clinical outcome after coronary artery bypass grafting. Results of a randomized trial. *Eur J Cardiothorac Surg* 2008;34:113-7.

[Hattler 2012] Hattler B, Messenger J, Shroyer AL, et al. Off pump coronary artery bypass surgery is associated with worse arterial and saphenous vein graft patency and less effective revascularization: results from the Veterans Affairs randomized on/off bypass (ROOBY) trial. *Circulation* 2012 (published online May 16, 2012)

[Hillis 2012] Hillis LD, Smith PK, Anderson JH, et al. 2011 ACCF/AHA guideline for coronary artery bypass graft surgery: executive summary. A report of the American College of Cardiology Foundation/American Heart Association Task Force on Practice Guidelines. *J Thorac Cardiovasc Surg* 2012;143:143-34.

[Kempfert 2011] Kempfert J, Rastan A, Leontyev S, et al. Current perspectives in endoscopic vessel harvesting for coronary artery bypass. *Expert Rev Cardiovasc Ther* 2011;9(11):1481-1488.

[Kiani 2011] Kiani S, Poston R. In endoscopic vein harvesting bad for saphenous vein graft patency in coronary surgery? *Curr Opin Cardiol* 2011;26:518-522

[Kiaii 2022] Kiaii B, Moon BC, Massel D, Langlois Y, Austin TW, Willoughby A, et al. A prospective randomized trial of endoscopic versus conventional harvesting of the saphenous vein in coronary artery bypass surgery. *J Thorac Cardiovasc Surg* 2002;123(2):204-12.

[Loop 1986] Loop FD, Lytle BW, Cosgrove DM et al. Influence of the internal mammary grafts on 10-year survival and other cardiac events. *N Engl J Med* 1986;78:3141-6

[Loop 1989] Loop FD, Lytle BW, Cosgrove DM. New arteries for old. *Circulation* 1989;79:40-45

[Lopes 2009] Lopes RD, Hafley GE, Allen KB, Ferguson TB, Peterson ED, Harrington RA, et al. Endoscopic versus open vein-graft harvesting in coronary-artery bypass surgery. *N Engl J Med* 2009; 361(3):235-44.

[Lopes 2012] Lopes RD, Mehta RH, Hafley GE, Williams JB, et al. Relationship between vein graft failure and subsequent clinical outcomes following coronary artery bypass surgery. *Circulation* 2012;125:749-756

[Lumsden 1996] Lumsden AB, Eaves FF 3rd, Ofenloch JC, Jordan WD. Subcutaneous, video-assisted saphenous vein harvest: report of the first 30 cases. *Cardiovasc Surg* 1996;4(6):771-6.

[Markar 2010] Markar SR, Kutty R, Edmonds L, Sadat U, Nair S. A meta-analysis of minimally invasive versus traditional open vein harvest for coronary artery bypass graft surgery. *Interactive CardioVascular and Thoracic Surgery* 2010;10:266-270

[Magee 2010] Magee MJ, Alexander JH, Hafley G, Ferguson TB Jr, Gibson CM, Harrington RA, et al. Coronary artery bypass graft failure after on-pump and off-pump coronary artery bypass: findings from PREVENT-IV. *Ann Thorac Surg* 2008;85(2):494-9; discussion 499-500.

[Motwani 1998] Motwani JG, Topol EJ. Aortocoronary saphenous vein graft disease. Pathogenesis, predisposition, and prevention. *Circulation* 1998;97:916-931.

[Oddershede 2012] Oddershede L, Andreasen JJ, Brocki BC, Ehlers L. Economic evaluation of endoscopic versus open vein harvest for coronary artery bypass grafting. *Ann Thorac Surg* 2012;93:1174-80

[Patel 2009] Patel NP, Angelini GD. Open or endoscopic vein graft harvesting – this is the question! *Nature Reviews/Cardiology* 2009;6:738-740.

[Perrault 2004] Perrault LP, Jeanmart H, Bilodeau L, Lespérance J, Tanguay JF, Bouchard D, et al. Early quantitative coronary angiography of saphenous vein grafts for coronary artery bypass grafting harvested by means of open versus endoscopic saphenectomy: a prospective randomized trial. *J Thorac Cardiovasc Surg* 2004; 127(5):1402-7.

[Puskas 2009] Puskas JD, Halkos ME, Balkhy H, Caskey M, Connolly M, Crouch J, et al. Evaluation of the PAS-Port proximal anastomotic system in coronary artery bypass surgery (the EPIC trial). *J Thorac Cardiovasc Surg* 2009;138:125-32.

[Puskas 1999] Puskas JD, Wright CE, Miller PK, Anderson TE, Gott JP, Brown WM 3rd, et al. A randomized trial of endoscopic versus open saphenous vein harvest in coronary bypass surgery. *Ann Thorac Surg* 1999; 68(4):1509-12.

[Rousou 2009] Rousou LJ, Taylor KB, Lu XG, Healey N, Crittenden MD, Khuri SF, et al. Saphenous vein conduits harvested by endoscopic technique exhibit structural and functional damage. *Ann Thorac Surg* 2009; 87(1):62-70.

[Sabik 2005] Sabik JF 3rd, Lytle BW, Blackstone EH, Houghtaling PL, Cosgrove DM. Comparison of saphenous vein and internal thoracic artery graft patency by coronary system. *Ann Thorac Surg 2005*;79:544-51.

[Sepehripour 2011] Sepehripour AH, Jarral OA, Shipolini AR, McCormack DJ. Does a "no touch" technique result in better vein patency? *Interactive Cardiovascular and Thoracic Surgery* 2011;13:626-630

[Serruys 2009] Serruys PW, Morice MC, Kappetein AP, Colombo A, SYNTAX Investigators. Percutaneous coronary intervention versus coronary artery bypass grafting for severe coronary artery disease. *N Engl J Med* 2009;360:961-972

[Serruys 2010] Serruys PW, Onuma Y, Garg S, et al. 5-year clinical outcomes of the ARTS-II. *J Am Coll Cardiol* 2010;55(1):1093-01

[Sianos 2005] Sianos G, Morel MA, Kappetein AP, et al. The SYNTAX Score: an angiographic tool grading the complexity of coronary artery disease. *Eurointerv* 2005;1:219-227.

[Shroyer 2009] Shroyer AL, Grover FL, Hattler B, Collins JF, McDonald GO, Kozora E, ROOBY Investigators. On-pump versus off-pump coronary-artery bypass surgery. *N Engl J Med* 2009; 361(19):1827-37. [VA CSP #517 main manuscript]

[Smith 2011] Smith SC, Benjamin EJ, Bonow RO, et al. AHA/ACCF secondary prevention and risk reduction therapy for patients with coronary and other atherosclerotic vascular disease. 2011 update. A guideline from the American Heart Association and American College of Cardiology Foundation. *Circulation* 2011; 124:2458-2473.

[Taggart 2006] Taggart DP. Coronary artery bypass grafting is still the best treatment for multivessel and left main disease, but patients need to know. *Ann Thorac Surg* 2006;82:1966-1975.

[Thatte 2001] Thatte HS, Khuri SF. The coronary artery bypass conduit: 1. Intraoperative endothelial injury and its implication on graft patency. *Ann Thorac Surg* 2001;72:S2245-52

[Tennyson 2010] Tennyson C, Young CP, Scarci M. Is it safe to perform endoscopic harvest? *Interactive CardioVascular and Thoracic Surgery* 2010;10:625-630.

[Thygesen 2012] Thygesen K, Alpert JS, Jaffe AS and the Joint ESC/ACCF/AHA/WHF Task Force for the Redefinition of Myocardial Infarction, Third Universal definition of myocardial infarction. *Circulation* 2012;126:2020-2035.

[Van Diepen 2013] Van Diepen S, Brennan JM, Allen KB, Hafley GE, et al. No difference in 1-year vein graft failure or 5-year clinical outcomes between open and closed tunnel endoscopic vein harvesting devices: results from the PREVENT-IV trial. Proceedings, The Society of Thoracic Surgeons 49th Annual Meeting, Los Angeles, CA. January 26-30 2013, p. 310.

[Yun 2005] Yun KL, Wu Y, Aharonian V, Mansukhani P, Pfeffer TA, Sintek CF, et al. Randomized trial of endoscopic versus open vein harvest for coronary artery bypass grafting: six-month patency rates. *J Thorac Cardiovasc Surg* 2005;129(3):496-503.

[Williams 2012] Williams JB, Peterson ED, Brennan JM, et al. Association between endoscopic vs open vein-graft harvesting and mortality, wound complications and cardiovascular events in patients undergoing CABG surgery. *JAMA* 2012;308(5):475-484

[Wjins 2010] Wijns W et al. Guidelines on Myocardial Revascularization. The task force on myocardial revascularization of the ESC/EACTS. *Eur Heart J* 2010;31:2501-2555.

[Zenati 2011] Zenati MA, Shroyer AL, Collins JF, Hattler B, Ota T, Almassi GH, Amidi M, Novitzky D, Grover FL, Sonel AF. Impact of endoscopic versus open saphenous graft harvest technique upon CABG patient outcomes in the randomized ROOBY trial. *J Thorac Cardiovasc Surg* 2011;141:338-44 [VA CSP #517 subanalysis]

[Zenati 2006] Zenati MA, Sonel A, Hattler B, Shroyer AL, Collins J, Messenger J, Baltz JH, Mohr L, Gabany JM, Novitsky D, Grover F. Patency outcomes of aortic connectors. *Innovations* 2006; 1(5):255-257 [VA CSP #517 subanalysis]

[Zenati 2007] Zenati MA, Sonel AF, Bjerke R, Shroyer L, Collins J, ROOBY Investigators. Safety of Aprotinin in CABG Patients: Results of the VA multicenter prospective randomized ROOBY trial. *Circulation* 2007;116(16):II-396[VA CSP #517 subanalysis]

[Zenati 2012] Zenati MA, Biswas K, Shroyer AL, Gaziano JM, Quin J, Haime M, Bhatt DL. Long-term patency of vein grafts harvested endoscopically for CABG: a meta-analysis. *J Am Coll Cardiol* 2012;59:E1450

Principal Investigator:

Title: RANDOMIZED ENDO-VEIN GRAFT PROSPECTIVE – REGROUP

Study Chair: Marco Zenati MD, MSc, FETCS

Co-Investigator:

INTRODUCTION

You are being invited to take part in a research study that is being funded by the Department of Veterans Affairs (VA). Before you decide to take part, you should know why the research is being done and how it will be performed. There may be potential risks and benefits if you decide to participate.

Please read this information closely. If you wish, discuss this study with family and friends. If there is anything that if you would like to understand better, ask to speak to someone from the study. Take your time in deciding if you want to participate. If you do decide to take part, we will ask you to sign the last page of this consent form. This means that you received all of the information below, were able to ask questions and discuss concerns with a member of the study team, and would like to take part in the study.

1. Purpose of study:

You are being invited to take part in this research study because it has you need to have coronary artery bypass graft (CABG) surgery. As part of this procedure, you will have a piece of vein removed from one of your legs. This is called vein harvesting. This research study will compare two methods of vein harvesting. Both methods are commonly used and considered safe but it is not known if one method is better than the other. We hope this study will allow us to learn if one method of vein harvesting improves the results of CABG surgery more than the other method.

One method is called “open vein harvesting” (OVH). In this technique, one or more incisions are made along the thigh and calf to remove a vein called the saphenous vein. The incisions may vary in length. With the OVH technique, the surgical team can avoid handling the vein more than necessary in removing it from the leg. After the vein is removed, the incision are sutured closed. The leg is wrapped in a large bandage that will be removed one or two days after the operation. A scar will be visible after the leg heals.

The other method of removing the vein is called "endoscopic vein harvesting" (EVH). This method of removing the vein is newer but has been used for over 10 years. In this technique, a small incision is made either above or below the knee. A small video camera, called an endoscope, is inserted into the incision. Special instruments are then used to remove the vein through this small incision. One or two smaller incisions are made either in groin or calf to help remove the vein. The leg is also wrapped in a large bandage that will be removed one or two days after surgery. The scar that is left by this surgery is smaller. In some cases, the incision heals more quickly and is less painful. Some studies have suggested that there are fewer wound problems when this method is used to harvest the vein.

Because the EVH technique appears to allow the leg to heal faster and causes fewer scars, this is now the more common method of harvesting the vein. However, surgeons have recently questioned if the EVH technique may injure the vein and cause it not to work as well over time. Examples of how this might happen include the heat from the endoscope or pressure from the instruments used during the procedure.

At the present time, it is not known if the EVH technique causes injury to the vein. If EVH actually does injure the vein, it is possible injury that OVH will be a better method of vein harvest. If OVH is a more gentle method of harvesting the vein, it may allow the vein to deliver more blood and oxygen to the heart which may be better for the long-term results of the CABG surgery.

If you agree to participate in this research study you will allow the method of vein harvesting for your surgery to be randomly chosen – such as deciding by using the flip of a coin. You will have a 50/50, or an equal chance of receiving either the open or endoscopic method of vein harvesting. To participate in this study, both you and your surgeon must agree that the method of vein harvest will be randomly picked on the day of surgery. This is the only part of your surgery that will be randomly chosen. However, if you or your surgeon prefers one technique over the other and would like to choose which technique you will have, you cannot participate in the study.

Principal Investigator:

Title: RANDOMIZED ENDO-VEIN GRAFT PROSPECTIVE – REGROUP

Study Chair: Marco Zenati MD, MSc, FETCS

Co-Investigator:

There are no experimental devices or drugs that will be used in this research study. The endoscopic devices used in vein harvesting are approved by the U.S. Food and Drug Administration and have been in use for several years. The instruments will not be used in any experimental manner.

This study will recruit approximately 1,150 research subjects from approximately 16 VA centers across the country. This VA site is one of 16 VA centers selected. Approximately 72 patients will be enrolled over three years at this site.

The study is funded by the Department of Veterans Affairs. The Chief of Cardiac Surgery at the VA Boston Healthcare System is in charge of the overall study (Study Chair). The VA sites will work with the VA Cooperative Studies Program at Perry Point, Maryland to collect and analyze data from this study. The surgeon performing your CABG procedure will oversee the vein harvest. This surgeon is one of the surgeon investigators of this research study for the hospital where you are having surgery.

2. Description of the study, procedures to be used, and how long it will last:
This research study is expected last approximately six years. Your active participation in the project may be as long as three years or may be as short as one year depending on when you enter the study. The study team may also collect information about you through electronic medical records and national public databases for the duration of the study.

If you decide to take part in this study, this is what will happen:

After signing this consent form, the surgeon investigator and study coordinator will review your records to confirm you are eligible to take part in the study. This is called screening. As part of the screening, the coordinator will look at your medical record. This includes, but is not limited to, your past medical history, the results of exams and tests including your heart catheterization and the evaluation by your surgeons.

Principal Investigator:

Title: RANDOMIZED ENDO-VEIN GRAFT PROSPECTIVE – REGROUP

Study Chair: Marco Zenati MD, MSc, FETCS

Co-Investigator:

Before going to surgery you will be asked to complete two baseline questionnaires for research purposes. This will take approximately 20 to 30 minutes. These questionnaires will ask you about your activity levels and how you feel both physically and emotionally. You will be asked to report about your personal feelings. You may skip any of the questions that you do not wish to answer.

You will also have blood pressure measurements of both arms and legs while lying on a table. This is called an Ankle-Brachial Index (ABI) test. This test checks the general condition of your arteries by measuring the difference in blood pressure from your arms compared to your legs. This test will show if there is decreased blood flow to your legs that may cause your leg incisions to heal more slowly or have problems after surgery. The quality of your leg veins will also be measured with a scoring system called venous clinical severity score in which we look at the quality of your veins. These two assessments will be performed at the same time and will take approximately 20 minutes to complete. These tests may be routinely performed for patients going to surgery but we will also collect and report this data for research purposes.

On the day of your surgery in the operating room, and under the supervision of the surgeon investigator, you will be randomized (like flipping a coin) to have the vein removed using either the EVH or OVH procedure. A member of the study team will call a phone number to obtain the randomization assignment. This will take about 10 minutes and will happen just before your surgery begins. You may or may not already be asleep with anesthesia. After you wake up, you will be able to see what type of vein harvest you received. It is possible that both methods may have been used in harvesting your vein.

Once the randomization assignment is known, your CABG surgery will proceed as usual. You will continue to receive the same care that you would receive even if you were not participating in this research study throughout your hospital stay.

The study team will collect information about you from your medical records during and after surgery until you are discharged from the hospital. On the first and second day after your surgery, you will

have an electrocardiogram - also known as an EKG. This test is routinely performed on CABG patients but the results will also be used for research purposes. If the EKG indicates there is new damage to your heart, known as a myocardial infarction (MI), then approximately two tablespoons of your blood will be drawn to check cardiac biomarkers. This is because cardiac biomarker levels will become increased in the blood when there is new heart damage. This test will be repeated every eight hours only if the level is increasing. Once it is determined the level is decreasing or remains in a normal range no further levels will be drawn. This blood test will help determine if you have had any new damage to your heart. Cardiac biomarker tests are frequently performed on CABG patients but the results will also be used for research purposes.

When you are discharged from the hospital, you will be asked to complete a survey that asks you about your leg incisions. When you return in 4-6 weeks for a follow-up visit to the cardiac surgery clinic, you will be asked to complete this survey again. If you had a problem with your leg incisions or any unexpected illnesses you will be asked to provide further information to the study team. If you were treated at a non-VA facility, you may be asked to sign an authorization for release of information from the treating facility. You will also be asked to complete the same two questionnaires that you completed before your operation. Overall, your follow up visit should take approximately one hour.

After your CABG follow-up visit, the study coordinator will contact you by phone every three months and ask you questions about your health. This call may be as short as five minutes or longer depending on how much has changed with your health status. You may also be contacted at other time points to clarify any questions about your health status. This will continue for at least one year and possibly longer depending on when you entered the study and your overall health. The study team will also collect information about your health from existing medical records and several different databases. These databases are listed on the following page, in the Confidentiality section of this consent form.

3. Reasonably foreseeable discomforts or inconveniences of the study:

- Before surgery, you will have ABI measurements as described above (on page 3), which will involve the use of blood pressure cuffs. The tight, squeezing sensation of the cuff on the arm or

leg is commonly uncomfortable. To minimize this mild discomfort, a properly sized blood pressure cuff will be used.

- You will have an electrocardiogram, or EKG, on the first and second day after your surgery. The pads used for the EKG may cause skin irritation and be uncomfortable when they are pulled off the skin. The technician is trained to remove the pads carefully.
- You will have blood drawn for the study. The amount taken will be about 1-2 tablespoons. This blood work will be drawn at the same time as your regular postoperative labs from a catheter already in place from your CABG surgery so no additional needle stick is required.
- You will be asked to complete questionnaires as a part of the study. You may feel uncomfortable answering some of the questions asked on the survey. You may refuse to answer any question that you do not want to answer.
- You will be asked to return to this facility's cardiac surgery clinic for follow-up so your incisions can be examined. This is standard for CABG patients but may be inconvenient if you live a far distance away from this VA facility.

4. Reasonably foreseeable risks of the study:

This consent form only discusses the risks of the research study. The risks associated with the CABG surgery, vein harvesting and anesthesia are not discussed in this consent form. You should talk with your health care providers about risks of the operation. Prior to surgery, the surgical team will discuss the risks of CABG surgery with you in detail. This will include a review of risks associated with the two methods of vein graft harvesting. An anesthesiologist will meet with you before surgery to discuss the risks of anesthesia, blood loss and blood transfusions. If you are a female who could become pregnant, you will have a pregnancy test prior to receiving surgery and anesthesia. This is standard care. If you are found to be pregnant, you will not be able to take part in this study.

Both vein harvesting techniques that are being studied are used commonly for CABG surgery. For this reason, randomizing to one technique over the other does not increase the risk of the CABG surgery.

Principal Investigator:

Title: RANDOMIZED ENDO-VEIN GRAFT PROSPECTIVE – REGROUP

Study Chair: Marco Zenati MD, MSc, FETCS

Co-Investigator:

Risks that are associated with some of the studies for this research are presented below. To understand the level of risk described below the following definitions are used:

- Common - Occurs in 10-25% of people (10 to 25 out of 100 people)
- Rare - Occurs in less than 1% of people (less than 1 out of 100 people)
- Not serious - Not expected to cause lasting harm
- Serious - Possibly causing serious or permanent harm

- There is that an unauthorized person views your health care information. This is called a breach of confidentiality. This risk of possibly serious but is rare. Steps will be taken to minimize this risk, as described in the Confidentiality section below.
- There is a risk that, during transfer of your health information, incorrect health information is obtained. This risk of possibly serious but is rare. Steps will be taken to minimize this risk, as described in the Confidentiality section below.

5. Expected benefits of study:
There are no known direct benefits to you for being in this study.

6. Other treatment available:
You may decide to not participate in this study and undergo your CABG surgery with you or your surgeon deciding the type of vein harvest method you will have instead of being randomized.

7. Use of research results and Confidentiality:
Information collected for this research study will be kept confidential as required by law. Results of the study may be published for scientific purposes, but your personal records and identity will not be revealed unless required by law. A description of this clinical trial will be available on the website: http://www.ClinicalTrials.gov, as required by U.S. Law. You can search this website at any time.

Administrative and healthcare utilization data about you will be collected from several national VA databases. The VA national data resources may include, but are not limited to:

- The National Patient Care Database
- VA-Medicare/Medicaid merge

- National Laboratory and Pharmacy extracts
- Corporate Data Warehouse (Health Data Repository)
- Medical Domain Web Services
- Patient Care Services Clinical Data Warehouse
- VA Surgical Care Improvement Project
- Surgical Quality Workflow Manager
- VA Computerized Patient Record System
- Veteran Informatics and Computing Infrastructure
- VHA Support Service Center
- VA Surgery Quality Improvement Program
- VA Vital Status File

Some of this information will be automatically transferred from the electronic medical record and existing databases to another. In order to ease data transfer and ensure that the correct information is being transferred, information technology (IT) initiatives are being developed. These developments will allow data to be exchanged more accurately and widely. Some of this data will be downloaded or transferred within the VA national databases or individual VA Medical Centers to the VA Central Research Database to be analyzed.

Healthcare information about you that is collected for this study may be shared with other VA investigators, other Federal health agencies, or academic institutions for research purposes. Your personal information will not be included in this data sharing. Before this data can be shared, it must be approved by a VA oversight committee and limits applied for the use of the data.

Information about you is protected in the following way:
Your research records will be kept indefinitely or until the law allows their destruction in accordance with the VA Record Control Schedule (www1.va.gov/VHAPUBLICATIONS/RCS10/rcs10-1.pdf). Records will be destroyed, when allowed, in the following manner.

- Paper records will be shredded
- Electronic records will be destroyed in a manner in which they cannot be retrieved.

Your data will be entered into a data repository and used for future studies approved by an IRB. This repository will be housed and protected by the same VA CSP Data Coordinating Center for the study in Perry Point, MD.

Your research records will be entered into a protected database that will be kept indefinitely after the study is completed. This is required by law. This includes your social security number which will allow for proper identification in using national databases. This information will be safeguarded as outlined by the Privacy Act and Freedom of Information Act.

In order to protect your personal information, any paper records related to your involvement in this research study will be stored in a locked file cabinet in a locked room. Only certain research personnel, including the local site surgeon investigator, research coordinator and the study chair, will have access to this data. Any electronic data will be stored on secured VA computer drives. Only authorized persons will have access to this data.

8. New Findings:
You will be informed of any significant new findings during the study that may affect you or change your mind about staying in the study.

9. Special circumstances:
You will not be required to pay for medical care and services related to this study but may be required to pay for other medical care and services that are part of your usual care and not related to the study. This may include co-payments related to other VA or non-VA services including but not limited to dental services, healthcare supplies, medicines, orthopedic or prosthetic appliances and domiciliary or nursing home care.

You will be compensated $50 when you present for your six week clinic visit for your time and effort while taking part in this study. You will need to consent to the release of personal identifying information to the Fiscal Office of the VA Boston Healthcare System. This includes your name, address, and social security number. It is required so we may provide this compensation. If payment is made to you by the VA an IRS Form 1099 will be generated. The Form 1099 will be generated regardless of the amount you are paid or whether you are paid by check or cash voucher.

10. Rights of Recourse:
In the event that you are injured as a result of your being in this research study, you have the right to receive medical care, including emergency treatment. This care or treatment is governed by federal law and VA policy. You also have the right to file any legal action - for example, if you believe there was negligence in the conduct of the study.

11. Study Monitoring:
Your VA research and medical records may examined by persons approved for this purpose. Examples of persons or groups that might access your information include the Human Studies Subcommittee of this VA facility, the Executive Committee and Data Monitoring Committee for the study and personnel from the VA Cooperative Studies Program (CSP). Because this research study involves things that are regulated by the FDA they may choose to access and inspect your records. There is also a CSP Site Monitoring, Auditing and Review Team that will monitor this trial for compliance. Other federal agencies may access your records as needed for oversight. Regardless, your healthcare information will only be accessible to authorized persons.

Principal Investigator:

Title: RANDOMIZED ENDO-VEIN GRAFT PROSPECTIVE – REGROUP

Study Chair: Marco Zenati MD, MSc, FETCS

Co-Investigator:

RESEARCH SUBJECT'S RIGHTS: I have read or have had read to me all of the above.

The study person named below has explained the study to me and answered all of my questions. I have been advised of the potential discomforts and risks of this study. I have been told of other choices of treatment that I could have.

I understand that if I have any medical questions related to this research study, I should call **Dr. ___________________________** during normal working hours.

I understand that if I have any general questions about this research study, I should call **Dr. ______________________________________** during normal working hours.

I understand that if I have any medical problems that might be related to this study **during normal working hours,** I should call **Dr.________________________. For any problems during non-working hours that are urgent, I should call ______________ and ask the hospital operator to page the on-call Cardiac Surgeon.**

I understand that, if at any point during or after this study I have any questions about my rights as a research subject or I want to discuss problems, complaints, concerns, and questions about this research, obtain information or offer input, I may contact the Research Compliance Officer after hours at ____________________.

I understand that my participation in this study is voluntary, that I do not have to take part in this study and that, if I do take part, I may withdraw from the study at any time. I also understand that, if I refuse to take part or if I decide to withdraw, I will not suffer any penalty, loss of rights, or loss of VA or other benefits that I have a right to receive.

Subject's Name: ____________________ , __________________ **Date:** ___________________

Last First

Soc. Sec. No. ______________________________________

(If research requires documentation in the medical record in accordance with VHA handbook 1907.1 the entire SSN must be obtained.)

VA FORM

JAN 1990 **10-1086**

____

Subject's Initials

VA IRB # ___________________

Approved: ___________________

Expires:

Form valid only if above completed

Principal Investigator:

Title: RANDOMIZED ENDO-VEIN GRAFT PROSPECTIVE – REGROUP

Study Chair: Marco Zenati MD, MSc, FETCS

Co-Investigator:

I voluntarily consent to be in this study. I will receive a signed copy of this consent form.

Subject's Signature	Month	Day	Year	Name (print)
Signature of Person Obtaining Consent	Month	Day	Year	Name (print)
Signature of Witness	Month	Day	Year	Name (print)

CSP#588 REGROUP
SITE (RESEARCH COORDINATOR) BUDGET
36 Months Recruitment
ONE YEAR FOLLOW-UP
August 16, 2012
BASED ON 2 PATIENTS ENROLLED PER MONTH PER SITE
16 SITES

	3 Month Start Up FY 13	Recruitment 12 months FY14	Recruitment 12 months FY 15	Recruitment 12 months FY 16	12 months F/U FY 17	3 months close out FY 18	TOTAL
Research Coordinator GS 11 1.00 FTE	$25,045.00	$105,190.00	$110,450.00	$115,972.00	$121,771.00	$31,965.00	$510,393.00
**SALARY TOTAL**	**$25,045.00**	**$105,190.00**	**$110,450.00**	**$115,972.00**	**$121,771.00**	**$31,965.00**	**$510,393.00**
Supplies	$1,500.00	$1,500.00	$1,500.00	$1,500.00	$1,500.00	$0.00	$7,500.00
IT Equipment	$2,000.00	$150.00	$150.00	$150.00	$150.00	$0.00	$2,600.00
Patient Payment			$3,600.00	$3,600.00	$3,600.00	$0.00	$10,800.00
Annual Travel Meetings $1,000 per person	$3,000.00	$3,000.00	$3,000.00	$3,000.00	$3,000.00	$2,000.00	$17,000.00
**GRAND TOTAL SUPPLIES/TRAVEL**	**$31,545.00**	**$109,840.00**	**$118,700.00**	**$124,222.00**	**$130,021.00**	**$33,965.00**	**$548,293.00**

CSP#588 REGROUP
SITE (RESEARCH COORDINATOR) BUDGET
36 Months Recruitment
ONE YEAR FOLLOW-UP
August 16, 2012
BASED ON 2 PATIENTS ENROLLED PER MONTH PER SITE
16 SITES

Justification for Patient Payments:

No payment for enrollment in this study will be provided. However, a small payment ($50.00) will be provided to all participants returning for their six week clinic visit. This payment is provided to assist the participant for time and travel expenses.

VA COOPERATIVE STUDY #588

RANDOMIZED ENDO-VEIN GRAFT PROSPECTIVE – REGROUP TRIAL

Guidance for the Definition of Type 5 Myocardial Infarction (MI related to CABG)

- The CSP#588 Executive Committee has recognized the need to clarify a working definition to be used in the REGROUP Trial for Myocardial Infarction (MI).
- The updated Third Universal Definition of MI published in 2012 was cited as a reference for the Executive Committee discussion [Thygesen 2012].
- The purpose of this clarification for the definition of MI is to capture every and all MI events during the conduct of the study while minimizing the volume of site reports that do not meet the definition of MI.
- As a modification to the currently approved protocol, **no** cardiac biomarkers (including troponin) will be routinely required as a study procedure at any time point (including baseline or postop). The case report forms will have indicators for these tests and corresponding reference ranges to be captured only if performed for clinical purposes and are available in the medical record of a consented subject for CSP#588.
- Electrocardiograms (12 lead ECG) will continue to be required on post-op day#1 and #2 as stated in the currently approved protocol, however these are performed for clinical purposes at every site at these same time points (POD#1 and POD#2) regardless of the study protocol.
- ECG abnormalities that indicate the need for cardiac biomarker workup include new pathologic Q waves or new left bundle branch block.
- In the event an ECG abnormality as described above, or clinical suspicion, causes concern for MI, cardiac biomarkers will be obtained. This is standard clinical practice. Cardiac Troponin I (cTn I) , cardiac Troponin T (cTn T) or CPK-MB will be obtained every 8 hours until a downward trend is seen in the level. The preferred cardiac biomarker is cTn I.
- The troponin will be considered indicative of MI if the peak level > 10 x 99th percentile of the upper reference limit (URL) **in the first 48 hours after CABG**. The existing principles [Thygesen Circulation 2012] for the universal definion of MI should be applied for the definion of MI > 48 hours after surgery.
- The cardiac biomarker level does not stand alone in the diagnosis of MI for post-op CABG patients, it must be accompanied in the same setting with **at least one of the following**:
    - 12 lead ECG with new pathologic Q waves or new left bundle branch block, or
    - angiographic documented new graft or new native coronary artery occlusion, or
    - imaging evidence of new loss of viable myocardium or new regional wall motion abnormalities.

- In the event cardiac biomarker work-up and ECG interpretations do not provide a complete diagnostic profile to the satisfaction of the surgeon investigator, a transthoracic echocardiogram would be the next clinical step to ascertain if a true MI occurred as evidenced by a new regional wall motion abnormality, with the exception of the septal wall.
- For the purposes of this study, septal wall motion abnormalities (which are prevalent in the postoperative phase) are excluded from the Definition of MI.

Introduction:

CSP #588 - REGROUP is a randomized, intent-to-treat, two-arm, parallel design, multicenter study. Cardiac Surgery Programs at Veterans Affairs Medical Centers (VAMC) with expertise in performing both EVH and OVH will be invited to participate in the study. Subjects requiring elective or urgent CABG using cardiopulmonary bypass with use of at least one SVG will be screened for enrollment using established inclusion/exclusion criteria. Enrolled subjects will be randomized to one of the two arms (EVH or OVH) after an experienced vein harvester is identified and assigned. Assessments will be collected at multiple time points including: intraoperatively, postoperatively, at discharge or 30 days after surgery if still hospitalized. Assessment of leg wound complications will be completed at the time of discharge and at six-week post-surgery. Telephone follow-ups will occur at three-month intervals post-surgery until the participating sites are decommissioned at the end of the trial period (which would be approximately 4.5 years after the site initiations). For long-term MACE outcomes, passive follow up for MACE events using VA databases (CPRS, VASQIP) will be performed centrally by the Study Chair's office for another 2 years.

This study will enroll approximately 1150 subjects requiring CABG at 16 VA Medical Centers with expertise in both techniques of vein harvesting. Assuming an enrollment rate of two subjects/medical center/month, total enrollment will take approximately three years to complete. With at least one-year follow-up period for the last subject randomized and two additional years of passive follow-up by the chair's office, the total duration of the study will be approximately six and half years.

Study Management at the CSPCC:

A Cooperative Studies Program Coordinating Center (CSPCC) study team has been assigned to CSP 588, the REGROUP trial, for providing data management, statistical, and administrative supports to the study executive committee for a smooth conduct and timely completion of the study. The study team is comprised of:

Biostatistician and Team Lead	Kousick Biswas, Ph.D.
Project Manager	Annette Wiseman
Statistical Programmer	Rebecca Horney
Database Programmer	Christine Dalzell
Computer Assistant	Daniel Briones
Computer Assistant	Mike Beam

Other core CSPCC staff, for example, Quality Assurance, Travel Clerk, Printer, Secretary, etc., will provide help based on the need of the study.

The Biostatistician is the study team leader and has the overall responsibility for the conduct of the study at the CSPCC. He is the CSPCC's spokesperson to the Study Group; he represents the CSPCC on the study's Executive Committee and along with the Study Chairperson, he is responsible for representing the study at the Data Monitoring Committee meetings. When un-blinded data are presented to the DMC, he is the only study representative allowed at those portions of the DMC meeting. The Biostatistician is also responsible for providing the Study Group with statistical and clinical trial advice, for working with other CSPCC team members in the preparation of routine interim reports, and for conducting the final analyses at the end of the study.

The Project Manager is responsible for the administrative coordination of the study by the CSPCC. She serves as the Biostatistician's Administrative Assistant and works with the CSPCC study team to ensure that all reports, study materials, and meeting arrangement notices are sent to the proper individuals in a timely fashion. She will work closely with the Project Director in the Chairman's office to ensure that the study runs smoothly and will be in contact with both the National Study Coordinator and the Local Research Coordinators at the participating centers at least monthly to discuss any problems that they may be having, including those with the CSPCC. She will also work with the local VA R&D Offices at the participating centers to obtain R&D and IRB approvals at the beginning of the study and annually as well as the preparation of study budgets yearly during the ongoing phases of the study.

The Statistical Programmer is responsible for the preparation of the tables and analyses for all of the routine study reports. These include Study Group, Executive Committee, Data Monitoring Committee, and the mid-study report to CSSEC. S/he also prepares the tables and reports for the final analyses. S/he works closely with the Biostatistician on these analyses.

The Database Management System (DBMS) Programmer is the lead of the data management support group and works closely with the assigned computer assistant(s) to address the data management need for the assigned study. S/he is responsible for establishing, updating and maintaining the study's database. In addition, s/he will write edit program based on an agreed upon edit plan that will thoroughly check the data for errors and missing information. S/he is also responsible for programming and maintaining the randomization system for the study.

The Computer Assistant(s) are responsible for setting up the data definition table for the study, laying out the electronic case report forms in the form design software, and validating all incoming data. They are also responsible for training the study staff at each site on how to properly manage the data collection process and how to appropriately respond to data edits. The computer assistant(s) are also responsible for working with the sites to resolve the data queries generated based on the incomplete and/or inaccurate data submitted to the study database.

Randomization and Data Management:

Randomization and Data management will be performed by the VA CSPCC Perry Point, MD. An Interactive Touchtone Telephone Randomization System (ITTRS) will be used to set up the randomization system. Clinical DataFax System, a data management software will be used for data management. The CSPCC will have overall responsibility for the data at the end of the study.

Randomization

After a patient at any of the participating centers is consented, successfully screened and has provided baseline information, s/he will be assigned to a harvester. Once a harvester is assigned and available for the harvesting of the required vein, the patient will be randomized. The research coordinator will place a call to the ITTRS (a dedicated 1-800 phone number will be provided) to randomize the patient in one of the two harvesting techniques – endoscopic and open vein harvesting techniques. Once the required information is entered in the system, the system will return the assignment for the patient. This study will use a “permuted block” randomization scheme where random block sizes of two and four will be used. The research coordinator will need the following information in order to complete a successful randomization call:

a. Study number and study password (will be provided by CSPCC))
b. 3-digit site number and password (will be provided by CSPCC)
c. The subject’s ID Number & ALPHA Code
d. The subject’s signed Informed Consent Form
e. Form 01, Screening and Randomization
f. Laminated Randomization Cheat Sheet

a. Data Capture during visits and/or telephone contacts

The system for data capturing will be designed by visits where a group of required CRFs will be assigned to each “visit” according to the “Schedule of Assessments” table.

FORM	SCREEN	BASELINE (pre op)	INTRA OP	POST OP	DC-30 DAY	6 WK	3 MO	6 MO	9 MO	12 Mo	…	45 MO	49 MO	AS NEEDED
00 –Screening Log	X													
01 – Screening and Randomization	X													
02 – Baseline Information		X												
03 – Seattle Angina Questionnaire		X				X				X				
04 – VR-12		X				X				X				
05 – Intraoperative Data Collection			X											
06 – Post Operative Assessments				X										
07 – Discharge Assessments					X									
08 – Leg Incision Pain Questionnaire					X									
09 – Leg Incision Pain 6 Week						X								
10 – Leg Incision Assessment					X	X								
11 – Mace Event (6 Week)						X								
12 – Phone Call Follow-up							X	X	X	X	X	X	X	
13 – MACE Event Form													X	
14 - Termination														X
15 - SAE														X
16 – SAE Follow-up														X
17 – Harvester Experience														X
18 – Protocol Noncompliance														X
19 – Confirmation of MI by Local Site														X
20 – Confirmation of MI by Clinical Events Committee														X
21 – Cause of Death by Clinical Events Committee														X
86 - Consent	X													

The draft versions of the CRFs can be found in Appendix E. The paper CRFs will be mailed to the sites. The research personnel will be filling up these CRFs during CABG, the 6-week clinic visit, and the 3-month phone calls. The completed CRFs will then be scanned in pdf and sent to Perry Point CSPCC via secure electronic server or posted on an ftp server. At CSPCC, the data management section staff will validate the CRFs once received by the DataFax system and will generate QC reports with listing of data discrepancies and other irregularities at regular intervals. These QC reports will be sent to the respective sites for clarifications and the site personnel will then submit “Refaxes” with clarification which will be validated and committed to the study master database (A “Refax” is a page of a CRF with corrections

which is sent back to the CSPCC by agreed upon mode of CRF transmission). The final responsibility for the completeness and accuracy of all study data collected at a participating site resides with the SI who will review all data before submission. The study database will be continuously updated with new data and changes to previously submitted data. To notify the participating sites about missing or late forms, reports with pertinent information will be generated at a regular interval and will be posted on site-accessible sub-SharePoint site.

In addition, a summary report of all data submitted and problems identified will be generated for each participating site. This report will provide each site with a summary of their progress. The National Study Coordinator in the Chairman's Office will also be reviewing each site's progress to ensure that there are no unforeseen problems with the forms or with a particular patient.

Another mechanism used to monitor the data and the progress of the study will be the preparation of periodic reports for various groups who are responsible for overseeing the conduct of the study. These groups include the Study Group, the Executive Committee, the Data Monitoring Committee, and the CSPCC Human Rights Committee, if applicable. These groups will receive study progress reports prior to their annual meetings and at least once in between their annual meetings. Thus, on average, these groups will receive a report every six months. The contents of these reports are discussed in the remainder of this appendix.

Monitoring Of Study By Study Group and Executive Committee:

The Study Group (all of the Sis, harvesters and research coordinators) and Executive Committee will meet six to nine months after patient recruitment begins and at annual intervals thereafter until the end of the study. Three weeks prior to these meetings and at six-month intervals between the meetings, these groups will be provided a report that will allow them to assess study progress. Since both groups are composed of study team members, no outcome data with harvesting group assignment (data that would potentially break the study blind) will be provided in these reports. These reports will contain information on:

a. Screening, Enrollment and Retention

The study team at each site will identify patients who might be candidates for the study. After the study has been explained to the patient and the patient signs the informed consent form, the screening process will be initiated. The research coordinator will complete the screening forms using the paper CRFs provided by the CSPCC Perry Point. If the patient meets all eligibility criteria, the baseline forms will be completed. The patient can then be randomized by completing the randomization form and placing a call to the ITTRS system (as described before) which will return the patient's harvesting group assignment – EVH or OVH.

The progress of patient accrual will be presented to the monitoring groups in three formats:

1. The study progress will be presented by site and for each site the following information will be provided:

    - actual number of patients entered into the study
    - expected number of patients to be entered at the time of the report, and
    - the percent of expected that were entered

    This format, as demonstrated in Table 1, will allow the Executive Committee to determine which sites are not recruiting as expected and the SIs to see how their site is doing in comparison with the others.

2. The study progress will be presented by the number of patients entered into the study by month (Table 2). These data will be organized by site. The data will indicate if recruitment is improving or worsening over time at the various sites. Sites whose intake is worsening can be detected and the SIs can be contacted to identify the reason for the recruitment deficit.

3. Recruitment data will be plotted over time as shown in Figure 1. The graph will be overlayed with the number of expected to be enrolled at the same time period.

TABLE 1: Number of Patients Entered and Number Expected

Site	Number Enrolled	Number Expected	Percent of Expected
1<br>2<br>.<br>.<br>.<br>16			
TOTAL			

TABLE 2: Patient recruitment by month

Month	Site 1	Site 2	...	Site 16
01/13<br>02/13<br>.<br>.<br>.				
TOTAL				

Figure 1: Observed Versus Expected Patient Recruitment

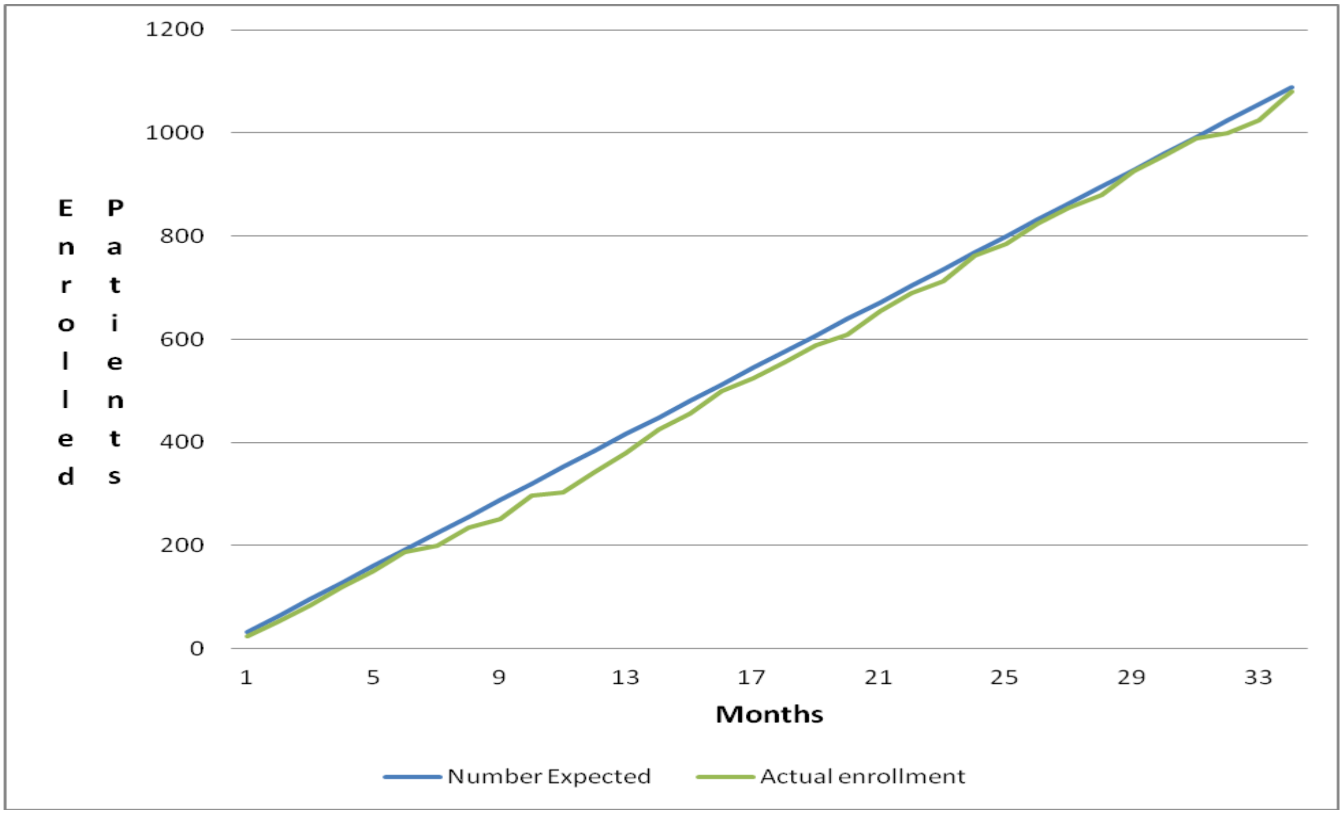

The number of patients screened, rejected and enrolled in the study will be presented in Table 3. The reasons for the exclusion of screened patients will be presented in Table 4.

TABLE 3: Cumulative Screening Summary: All Patients by Site

Site	Screened	Rejected	Enrolled	% Rejected
1<br>2<br>.<br>.<br>16				
TOTAL				

TABLE 4: Summary of Ineligibility: Reasons for Exclusion,
Total and By Site
TOTAL NUMBER SCREENED = ____________

Reason	No. Excluded	% of Screened
1. Combined valve procedure planned		
2. Moderate or severe valve disease		
3. Hemodynamically unstable or in cardiogenic shock.		
4. Enrolled in a different therapeutic study (w/o special exception approved)		
.		
.		
.		
8. Inability to provide informed consent		

b. Background Characteristics at Entry

Background characteristics of the screened/enrolled study patients are collected on the Screening Record Form. Tables summarizing the important background characteristics by site will be prepared and submitted to the Study Group to provide an idea of the population being studied and based on this information, comparisons of the patient characteristics among the sites will be possible. This information will be presented as means and medians for continuous variables (e.g., age) and as frequency tables for discrete variables (e.g., Gender). Table 5 shows how this data will be presented. Analysis of variance and chi-square techniques will be used to identify any statistically significant differences that may exist between the sites.

Table 5: Demographic Variables by Center for CSP#588

**Variables**	**Participating Centers**					
	**1**	**2**	**…**	**15**	**16**	**Total**
**Age** (Mean(SD))						
**Gender** (N (%))						
Male						
Female						
**Race** (N (%))						
American Indian/Alaskan						
Asian/Pacific						
Black						
White						
Other						
**Marital Status** (N (%))						
Married/Remarried						
Divorced						
Separated						
Widowed						

Never Married						
**Education** (N (%))						
Graduate/Professional						
Std. College/Univ. degree						
Partial College training						
High School						
<High School						

c. Data Quality and Protocol Adherence

The final type of information that will be provided to the Study Group is data that will allow the group to assess the quality of the data being submitted and how well the sites, in general, are adhering to the protocol. These data will also be presented by site, so sites performing substantially below average can be identified and remedial action can be taken to improve performance.

One piece of information that will be routinely provided is the number of forms that are missing according to the patient's assessment schedule. Table 6 indicates how this information will be displayed.

TABLE 6: Number of Missing Forms

**# of Patients**			**Site**				
			**1**	**2**	**…**	**16**	**Total**
Form 01	N	**%**					
Form 02	N	%					
.							
.							
.							
Form 21	N	%					

Study Monitoring By Data Monitoring Committee:

An independent oversight committee called the Data Monitoring Committee (DMC) will monitor study progress. This committee meets on the same basic schedule as the Study Group and Executive Committee, i.e., at 6 to 9 months after the start of patient recruitment and yearly thereafter. Initially, the DMC will meet once prior to the study start-up to acquaint themselves with the study and to establish monitoring guidelines. This committee does not usually meet during the last six months of a study.

The main responsibility for the DMC members is to make a recommendation to the Director of the Cooperative Studies Program on whether the study should continue or not based on the reviews of the progress reports submitted to them. The study could be recommended for termination due to poor recruitment, difference so large that it would be possible to reach a final decision about the main question of the study, difference so small that continuation would be irresponsible, and due to safety concerns of the procedures that are being investigated. The DMC also reviews the participating sites' performance in terms of recruitment, adherence to the protocol etc., and makes recommendations on them. Their final responsibility is to review all proposed protocol changes and suggested sub-protocols and to make recommendations in regards to their acceptability.

In order for the DMC to carry out its responsibilities, the CSPCC Study Team will provide the committee with a report approximately three weeks prior to their meetings. The report will consist of the tables described previously for the Study Group and Executive Committee reports as well as those presenting outcome analyses. It is the responsibility of the CSPCC Study Team to provide the DMC with whatever information the Board feels that it needs to successfully monitor the study. Thus, additional tables will be added as required by the DMC. In addition to the reports for the yearly meetings, the DMC will also be provided with reports between meetings at 6-month intervals.

In order for the DMC to make its recommendation for continuation of the study, it will be necessary for them to see the analyses for the primary outcome measure every time that the report is run and it is possible to calculate the primary outcome measure. Periodic monitoring of interim results can significantly affect the probability of making an incorrect decision. A number of formal techniques have been developed for interpreting interim results. At the organizational meeting, the DMC will select the technique that it wants to use to monitor the study. Suggested techniques are the Haybittle-Peto and Lan-DeMets group sequential boundaries. For the Haybittle-Peto method, a constant z-statistic is used as the monitoring boundary. The Lan-DeMets procedure produces decision boundaries that are quite conservative over the first several looks and then gradually converges to the nominal alpha levels as the final look is approached. Figure 2 gives an **example** of the Lan-DeMets boundaries for six looks at an alpha level of 0.05.

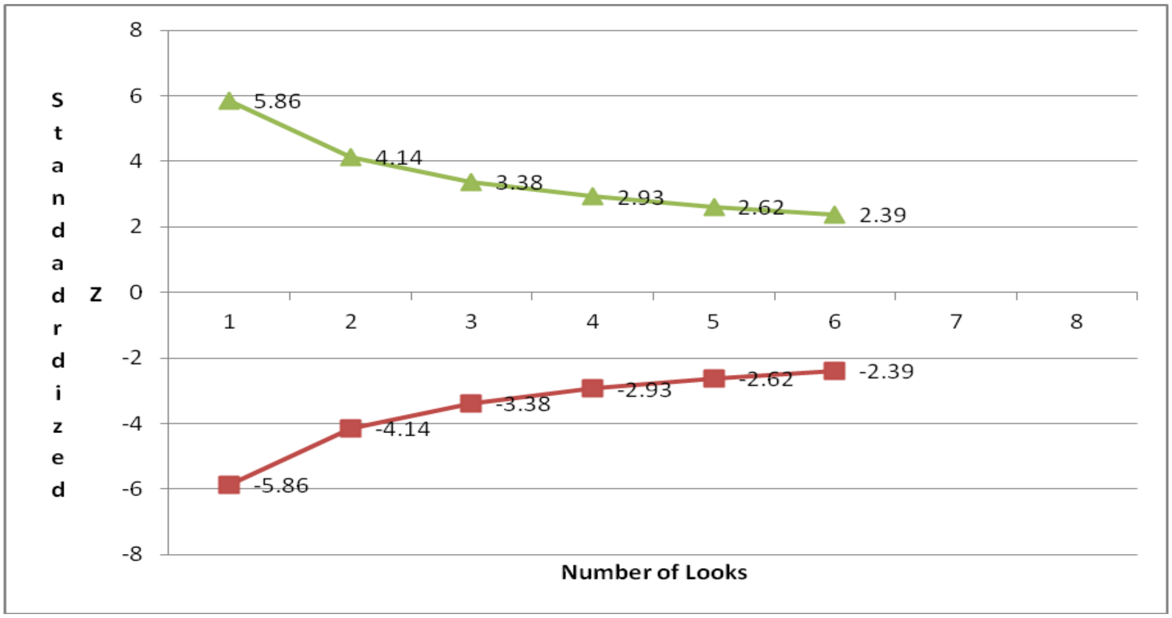

Figure 2: Lan-Demets Decision Boundary

The patient characteristics would be presented by site and by harvesting technique group for the DMC. Significant imbalances of these patient characteristics between the harvesting technique groups may indicate a need to use these characteristics as covariates during the analysis of the outcome measures. Formal testing of the differences between groups will be done at the study's conclusion using appropriate statistical tests - analysis of variance technique will be used to test characteristics that are continuous in nature, while chi-square technique will be used for the test characteristics that are discrete in nature.

As with any clinical trial, the safety of the patient will be of utmost concern. Safety will be monitored closely during the course of the study. The DMC Report will include data on incidence of adverse events by treatment group. It will also include data on early terminations and treatment dropouts. The adverse event data will also be reported in the primary study manuscript. Data will be collected on adverse events throughout the study starting immediately after the patient signs the informed consent form.

Outcome Measures:

The DMC reports and the final statistical report will include the statistical analyses of the primary and the secondary outcome measures for the study. The following paragraphs describe the

outcome measures, the statistical techniques for the analyses and the table shells in which the results of the analyses will be presented in the reports.

Primary Outcome Measure:

Time-to-MACE event: MACE, the composite endpoint that includes Death (all cause), Myocardial Infarction (MI) and Revascularization for myocardial ischemia, will be the primary outcome measure after randomization and the index CABG. Each randomized subject (either in Endoscopic or in Open harvesting group) will be followed after the index CABG to capture the time-to-MACE event where an 'EVENT' will be defined as either death (all cause) or an MI or a revascularization procedure during the follow-up period. Total follow-up period will be 6.5 years of which the first 4.5 years will be active follow-up (using in-clinic visit or by telephone) period and will be carried out by the site personnel. The remaining 2 years will be passive follow-up which will be carried out centrally by the chair's office staff using the VA patient database. A minimum of 1 year of active follow-up will be used for subjects who will be enrolled almost at the tail end of the 3-year projected enrollment period of the study. The subjects who will either be lost to follow-up or will not experience an 'EVENT' before the end of the follow-up period will be considered as right censored.

The primary analysis of such time-to-Mace event data will include the events only from the active follow-up period (which is 4.5 years). The secondary analysis will include events from the entire 6.5 years of follow-up period.

Survival analysis techniques will be used to analyze the time-to-MACE event data for both primary and secondary analyses.

Kaplan-Meier analysis, a nonparametric method, will be used to estimate the survival (not experiencing MACE) over time in the two harvesting groups and a log-rank statistic will be used to test the equality of the survival function estimates in the two groups (the null hypothesis).

Kaplan-Meier curves for all other predictors, e.g., age, gender etc., will also be plotted to provide insight into the shape of survival function for each group. These curves will also provide information about the proportionality among the groups (i.e., whether the curves are parallel or not).

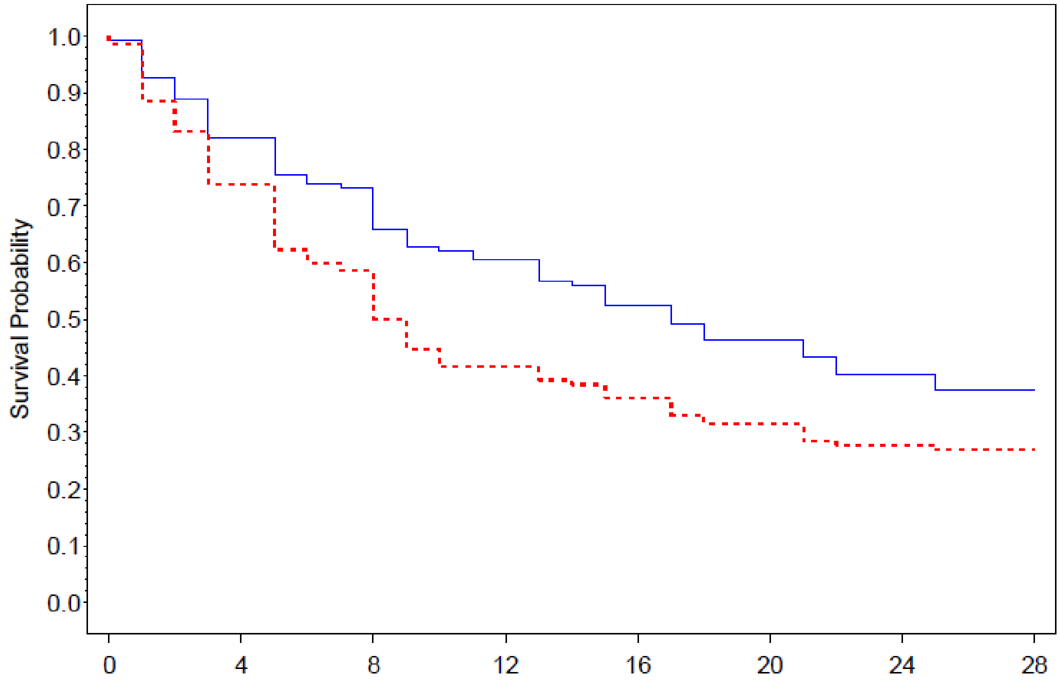

Figure 3: Typical Kaplan-Meier curve comparing two treatment groups

A typical Kaplan-Meier curve showing survival probabilities in two treatment groups is shown in Figure 3.

A test of equality across strata will also be performed to decide which predictor variables to include in the final model. For categorical variables log-rank tests, a non-parametric test, will be used and for continuous variables univariate Cox proportional hazard regression, a semi-parametric model, will be used. The results from the log-rank tests and univariate Cox proportional hazard regressions will be presented in the formats as shown in Tables 7 and 8.

Table 7: Test of Equality over Strata

Variable	Test (Log-Rank/MLE)	Chi-Square	Df	Pr > Chi-Square

Cox's Proportional Hazards models will be used to investigate the effect of harvesting technique on the time until MACE adjusted for other potential influential variables, such as, age, gender, harvester's experience etc. The predictors that are identified in the previous step will be included in the model. The results from the modeling will be presented in the format as shown in Table 8.

Table 8: Cox Proportional Hazards Model to evaluate effect of EVH/OVH on time-to-MACE adjusted for predictor variables

Variable	Df	Parameter Estimate	Standard Error	Chi-Square	Pr > Chi-Square

Secondary Outcome Measures:

1. **One-year MACE**:
   Patients who will suffer any one of these three outcomes within the first year after index CABG will be counted towards calculating the proportions of patients with MACE (yes/no) in each harvesting technique group – patients with endoscopic vein grafting or patients with open vein grafting. Only the first event will be considered as a MACE event. These two proportions will be compared using a chi-square test. MACE data at one-year post surgery will be presented as shown in Table 9. Tables 10, 11 and 12 provide the format for the components of MACE – for example, death (all cause), MI, and repeat revascularization.

Table 9: MACE distributions in harvesting technique groups at one-year post surgery

Harvesting / MACE	OVH	EVH	Total
No			
Yes			
Total			
ChiSq =	p-value =		

Table 10: Death (all cause) distributions in harvesting technique groups

Harvesting / Death (all Cause)	OVH	EVH	Total
No			
Yes			
Total			
ChiSq =	p-value =		

Table 11: MI distributions in harvesting technique groups

Harvesting / MI	OVH	EVH	Total
No			
Yes			
Total			
ChiSq =	p-value =		

Table 12: Revascularization distributions in harvesting technique groups

Harvesting / Revascularization	OVH	EVH	Total
No			
Yes			
Total			
ChiSq =		p-value =	

2. **Three-year MACE**:
   Patients who will suffer any one of these three outcomes within the first three years after index CABG will be counted towards calculating the proportions of patients with MACE (yes/no) in each harvesting technique group – patients with endoscopic vein grafting or patients with open vein grafting. Only the first event will be considered as a MACE event. These two proportions will be compared using a chi-square test. MACE data at three-year post surgery will be presented as shown in Table 13. Tables 14, 15 and 16 provide the format for the components of MACE – for example, death (all cause), MI, and repeat revascularization.

Table 13: MACE distributions in harvesting technique groups at two-year post surgery

Harvesting / MACE	OVH	EVH	Total
No			
Yes			
Total			
ChiSq =		p-value =	

Table 14: Death (all cause) distributions in harvesting technique groups

Harvesting / Death (all Cause)	OVH	EVH	Total
No			
Yes			
Total			
ChiSq =	p-value =		

Table 15: MI distributions in harvesting technique groups

Harvesting / MI	OVH	EVH	Total
No			
Yes			
Total			
ChiSq =	p-value =		

Table 16: Revascularization distributions in harvesting technique groups

Harvesting / Revascularization	OVH	EVH	Total
No			
Yes			
Total			
ChiSq =	p-value =		

3. **Post-operative Leg Wound Healing Complications:** Each patient in either harvesting technique group – EVH or OVH – will be examined for healing complications for their leg wounds from harvesting at discharge and at 6 weeks post-surgery. Post-operative leg wound healing complication status (yes/no) will be recorded at the discharge and also at 6 weeks post-surgery. Proportion of patients with leg wound healing complications will be computed for each treatment group and these proportions at each time point will be compared using chi-square tests. The impact of confounding variables, such as, BMI, diabetes status, smoking status, and harvester experience on the post-operative leg wound healing complications will be analyzed using a logistic regression. The results from the chi-square analysis will be presented with counts of 'yes' and 'no' in regards to leg wound healing complications in the two harvesting techniques as shown in Table 17.

Table 17: Leg Wound Healing Complications distributions in harvesting technique groups

Harvesting / Leg Wound Healing Complications	OVH	EVH	Total
No			
Yes			
Total			
ChiSq =		p-value =	

Table 18 provides the format in which the logistic regression analysis results for the leg wound healings complications will be presented. Each of the covariates in the model will be presented with individual odds ratio and 95% Confidence Interval.

Table 18: Logistic Regression Analysis of Leg Wound Healing Complications Data at 6-week Post Surgery

	Odds Ratio	95% Confidence Interval	
		LL	UL
Harvesting Technique			
BMI			
Harvester Experience			
Age of patient			
Diabetes Status			
Smoking Status			

4. **Severity of Incisional Leg Pain:** Severe leg pain, due to incisions made during vein grafting, data will be collected at discharge and at six-week post CABG. Proportion of patients with severe pain (pain score 3 and above = yes) at each time point will be compared using chi-square statistics.

   The results from the chi-square analysis will be presented with counts of 'yes' and 'no' in regards to severity of incisional leg pain in the two harvesting techniques as shown in Table 19.

Table 19: Severity of Incisional Leg Pain distributions in harvesting technique groups

Harvesting / Severity of Leg Pain	OVH	EVH	Total
No			
Yes			
Total			
ChiSq =		p-value =	

5. **Quality of Life:** QoL scores, from VR-12 and Seattle Angina Questionnaire, will be computed at baseline, six-weeks, and 12 months post-surgery for the patients in both harvesting technique groups (EVH and OVH). Patients in the two groups will be categorized as “improved”, “no change” and “worse” based on their baseline scores. The proportions of patients in these three categories will be compared between the two groups using chi-square statistics. The actual scores from these measures will also be used to compare patients in the two harvesting technique groups using analyses of covariance techniques, where the baseline (pre-surgery) scores will be used as a covariate. The results from the chi-square analysis will be presented with counts of ‘yes’ and ‘no’ in regards to QoL scores categorized as “improved”, “no change” and “worse” in the two harvesting techniques as shown in Table 20. Table 21 provides the format in which the results from ANCOVA analysis will be presented.

Table 20: QoL/SAQ score distributions in harvesting technique groups

Harvesting / QoL score	OVH	EVH	Total
Improved			
No Change			
Worse			
ChiSq =		p-value =	

Table 21: QoL/SAQ Scores by Harvesting Technique

	QoL Score		
**Treatment Group**	**N**	**Mean**	**Standard Error**
OVH			
EVH			

**Covariate**	**Estimate**	**Std. Error**	**t-statistics**	**p-values**
Harvesting Technique				
Baseline QoL Score				

**Source**	**Df**	**F Statistics**	**p-value**
Harvesting Technique			
Baseline QoL score			

SITE NO.	SUBJECT ID	ALPHA CODE	DATE FORM COMPLETED
☐☐☐	☐☐☐☐	☐☐☐☐	☐☐ Month ☐☐ Day ☐☐☐☐ Year

FORM 01 - SCREENING RECORD

***Complete this form for all subjects undergoing a CABG procedure at your site and submit to the Perry Point Cooperative Studies Program within 48 hours of CABG procedure.**

INCLUSION CRITERIA

	No	Yes
1. Age 18 years or older?	☐	☐
2. Elective or Urgent CABG-only (refer to protocol definition)?	☐	☐
3. Median sternotomy approach?	☐	☐
4. At least one coronary bypass planned using saphenous vein graft for conduit?	☐	☐
5. Experienced EVH/OVH harvester and participating surgeon available for procedure?	☐	☐

Answers to questions 1-5 must all be 'YES' for the subject to be eligible for enrollment

EXCLUSION CRITERIA

	No	Yes
6. Combined valve procedure planned?	☐	☐
7. Moderate or severe valve disease (see definition of moderate/severe valve disease)?	☐	☐
8. Hemodynamically unstable or in cardiogenic shock?	☐	☐
9. Enrolled in another therapeutic or interventional study? (without special approval obtained for enrollment)	☐	☐
10. Off-pump CABG procedure planned?	☐	☐
11. Limited life expectancy less than 1 year?	☐	☐
12. History of lower extremities venous stripping or ligation?	☐	☐
13. Inability to provide informed consent?	☐	☐

Answers to questions 6-13 must all be 'NO' for the subject to be eligible for enrollment

SITE NO.	*SUBJECT ID*	*ALPHA CODE*
☐☐☐	☐☐☐☐	☐☐☐☐

ENROLLMENT *No* *Yes*

14. Is the subject eligible to be enrolled into the REGROUP study? .... ☐ ☐

If the answer to question 14 is NO, then STOP, SIGN form - this form is now complete.

15. If the subject is **eligible but not enrolled**, mark (x) in the one box that best describes the reason:

- ☐ *Subject refused to sign Informed Consent*
- ☐ *Eligibility status changed (e.g., died, treatment plan changed, etc.)*
- ☐ *Site surgeon concerned about resources needed for on-site follow-up (cost, time, etc)*
- ☐ *No reliable method of follow-up contact with the subject (e.g., no phone, etc.)*
- ☐ *Primary reason subject preferred non-enrollment*
  - ☐ *Subject prefers open vein harvest*
  - ☐ *Subject prefers endoscopic vein harvest*
  - ☐ *Reason other than vein harvest preference*

    *specify:*____
- ☐ *Primary reason surgeon preferred non-enrollment*
  - ☐ *Surgeon prefers open vein harvest for subject*
  - ☐ *Surgeon prefers endoscopic vein harvest for subject*
  - ☐ *Reason other than vein harvest preference.*

    *specify:*____

If the subject was not enrolled in the study, STOP, SIGN form - this form is now complete.

RANDOMIZATION - If the subject is eligible and willing to be randomized, call CSPCC at 410-642-1736

No *Yes*

16. Was the subject randomized into the study? .... ☐ ☐

If Q 16 is Yes then go to Q 18. If Q 16 is No then go to Q 17 and the form is complete when signed.

SITE NO. ☐☐☐ ***SUBJECT ID*** ☐☐☐☐ ***ALPHA CODE*** ☐☐☐☐

17. If the subject has signed the consent but was not randomized, mark (x) in the one box that best describes the reason:

☐ *Eligibility status changed*

*explain:*______________________________

☐ *Subject changed mind after consent signed*

*explain:*______________________________

☐ *Surgeon changed mind after subject signed consent*

*explain:*______________________________

☐ *Other reason (specify):*______________________________

18. If the subject **is randomized**, complete the following information:

a. Date subject was randomized .... ☐☐ ☐☐ ☐☐☐☐
Month Day Year

b. Time subject was randomized (Military Time) .... ☐☐ ☐☐
hour : min

c. Assigned harvester ID Number .... ☐☐

d. Assigned surgeon ID Number .... ☐☐

e. Envelope Number .... ☐☐ - ☐☐☐

f. To what treatment was subject randomized? .... **EVH** ☐ **OVH** ☐

S.I. or Participating Surgeon Signature ______________________________

Print Name: ______________________________

Print name of the person completing this form if other than the site coordinator: ____________________

SITE NO.	*SUBJECT ID*	*ALPHA CODE*	*DATE OF ASSESSMENT*
☐☐☐	☐☐☐☐	☐☐☐☐	☐☐ *Month* ☐☐ *Day* ☐☐☐☐ *Year*

FORM 02 – BASELINE INFORMATION

COLLECT DATA FROM THE EXISTING MEDICAL RECORD FOR RANDOMIZED SUBJECTS.

IDENTIFICATION DATA

1. DATE OF BIRTH: ☐☐ *month* ☐☐ *day* ☐☐☐☐ *year*

DEMOGRAPHIC DATA

2. GENDER:

☐ Male ☐ Female

3. RACE:

☐ American Indian or Alaskan Native

☐ Asian or Pacific Islander

☐ Black, not of Hispanic origin

☐ Hispanic

☐ White, not of Hispanic origin

☐ Other, specify: ___________________________

4. MARITAL STATUS:

☐ Married/remarried

☐ Divorced

☐ Separated

☐ Widowed

☐ Never married

5. EDUCATION:

☐ Completed Graduate/professional training

☐ Standard college/university graduate

☐ Partial college training

☐ High school graduate/GED

☐ < High school

SITE NO.	SUBJECT ID.	ALPHA CODE
☐☐☐	☐☐☐☐	☐☐☐☐

MILITARY SERVICE HISTORY:

6. Served in the military? ☐ No ☐ Yes
   a. If Yes, when did the patient serve: (mark 'x' all that apply)
      ☐ World War I ☐ Balkans conflict
      ☐ World War II ☐ Afghanistan conflict
      ☐ Korean conflict ☐ Iraq conflict
      ☐ Vietnam conflict ☐ Other war/conflict, specify: ____________________
      ☐ Gulf War ☐ Peace time
   b. If Yes, served outside the U.S.: ☐ No ☐ Yes
   c. If Yes, specify branch:
      ☐ Army ☐ Coast Guard
      ☐ Air Force ☐ National Guard (active duty)
      ☐ Navy ☐ Merchant Marine
      ☐ Marines

PRE-OPERATIVE CLINICAL DATA, CO-MORBIDITIES

7. Diabetes: ☐ No ☐ Yes
   a. If Yes, how controlled? ☐ None ☐ Diet ☐ Oral Hypoglycemic ☐ Insulin
8. Hypertension: ☐ No ☐ Yes
9. Hyperlipidemia: ☐ No ☐ Yes
10. Depression : ☐ No ☐ Yes
11. Chronic Renal Disease: ☐ No ☐ Yes
    a. If Yes, requires dialysis? ☐ No ☐ Yes
12. Chronic Liver Disease: ☐ No ☐ Yes
13. Peripheral Vascular Disease: ☐ No ☐ Yes
14. Cerebral Vascular Disease: ☐ No ☐ Yes
    a. If Yes, CVA? ☐ No ☐ Yes
    b. If Yes, specify interval: ☐ ≤ 2weeks ☐ > 2weeks

SITE NO.	*SUBJECT ID.*	*ALPHA CODE*
☐☐☐	☐☐☐☐	☐☐☐☐

15. Chronic Lung Disease: ☐ No ☐ Yes
    a. If Yes, FEV_1: ☐☐☐ % predicted

16. Immunosuppressive Therapy: ☐ No ☐ Yes

17. Tobacco use > 100 cigarettes/lifetime: ☐No ☐ Yes
    a. If Yes, last use prior to surgery:
    ☐ < 7 days ☐ ≥ 3 months but < 1 year
    ☐ ≥ 7 days but < 3 months ☐ ≥ 1 year

18. Alcohol use > 1 ounce/day: ☐ No ☐ Yes
    a. If Yes, last use prior to surgery:
    ☐ < 7 days ☐ ≥ 3 months but < 1 year
    ☐ ≥ 7 days but < 3 months ☐ ≥ 1 year

19. Other major comorbidity: ☐ No ☐ Yes
    a. If Yes, specify: ____

20. Residential Status (resides with):
    ☐ Spouse ☐ Assisted Facility
    ☐ Family/other ☐ Alone

21. Functional Status: (ability to complete activities of daily living)
    ☐ Independent ☐ Needs Assistance ☐ Dependent

CARDIAC STATUS

22. Previous CABG Procedure: ☐No ☐ Yes If Yes, Date: ☐☐ ☐☐ ☐☐☐☐
    month *day* *year*

23. Previous Valve Procedure: ☐No ☐ Yes If Yes, Date: ☐☐ ☐☐ ☐☐☐☐
    month *day* *year*

SITE NO. ☐☐☐ *SUBJECT ID.* ☐☐☐☐ *ALPHA CODE* ☐☐☐☐

24. Previous PCI Procedure: ☐ No ☐ Yes If Yes, Date: ☐☐ *month* ☐☐ *day* ☐☐☐☐ *year*

a. If Yes, specify interval: ☐ ≤6 hours ☐ >6 hours

25. Myocardial Infarction: ☐ No ☐ Yes If Yes, Date: ☐☐ *month* ☐☐ *day* ☐☐☐☐ *year*

a. If Yes, specify interval:

☐ ≤ 6 hours ☐ >7 days but ≤ 21 days

☐ >6 hours but ≤ 24 hours ☐ >21 days

☐ >1 day but ≤7 days

26. Peak Cardiac Biomarkers (if available); ☐ No ☐ Yes If Yes, Date: ☐☐ *month* ☐☐ *day* ☐☐☐☐ *year*

If yes, you must complete A, B, OR C below

A. Peak Cardiac Troponin I

1. Peak Cardiac Troponin I (ng/mL): ☐☐.☐☐
2. Reference Range (ng/mL): ☐.☐☐ to ☐.☐☐

B. Peak Cardiac Troponin T

1. Peak Cardiac Troponin T (ng/mL): ☐☐.☐☐
2. Reference Range (ng/mL): ☐.☐☐ to ☐.☐☐

C. Peak CPK/MB Panel

1. Peak Total CPK (U/L): ☐☐☐☐
2. Total CPK Reference Range (U/L): ☐☐ to ☐☐☐
3. Peak CK-MB (ng/mL): ☐☐.☐
4. CK-MB Reference Range (ng/mL): ☐ to ☐.☐
5. Peak CK-MB Index (%): ☐☐.☐
6. CK-MB Index Reference Range (%): ☐.☐ to ☐.☐

SITE NO. ☐☐☐ *SUBJECT ID.* ☐☐☐☐ *ALPHA CODE* ☐☐☐☐

27. Heart Rate by ECG: ☐ <100 ☐ $\geq$100 Date: ☐☐ *month* ☐☐ *day* ☐☐☐☐ *year*

28. Arrhythmia by ECG: ☐ No ☐ Yes If Yes, Date: ☐☐ *month* ☐☐ *day* ☐☐☐☐ *year*
    a. If Yes, specify:
    ☐ Atrial fibrillation ☐ Pacemaker
    ☐ Atrial Flutter ☐ Other, specify: ________________

29. Cardiomegaly by CXR: ☐ No ☐ Yes If Yes, Date: ☐☐ *month* ☐☐ *day* ☐☐☐☐ *year*

30. Ejection fraction by ECHO: ☐☐☐ % Date: ☐☐ *month* ☐☐ *day* ☐☐☐☐ *year*

31. LV aneurysm by ECHO: ☐ No ☐ Yes If Yes, Date: ☐☐ *month* ☐☐ *day* ☐☐☐☐ *year*

32. Presentation on Admission: (mark only one)
    ☐ No symptoms or angina ☐ Unstable angina
    ☐ Symptoms, non-ischemic ☐ Non-ST elevation MI
    ☐ Stable angina ☐ ST elevation MI
    a. If experiencing angina classify by CCSS: ☐ Class I ☐ Class II ☐ Class III ☐ Class IV

33. Congestive Heart Failure: ☐ No ☐ Yes
    a. If Yes, classify by NYHA: ☐ Class I ☐ Class II ☐ Class III ☐ Class IV

34. Presence of Pulmonary Rales: ☐ No ☐ Yes If Yes, Date: ☐☐ *month* ☐☐ *day* ☐☐☐☐ *year*

35. Infectious Endocarditis: ☐ No ☐ Yes If Yes, Date: ☐☐ *month* ☐☐ *day* ☐☐☐☐ *year*
    a. If Yes, specify type: ☐ Treated ☐ Active

36. Cardiogenic Shock: ☐ No ☐ Yes If Yes, Date: ☐☐ *month* ☐☐ *day* ☐☐☐☐ *year*

SITE NO. ☐☐☐ *SUBJECT ID.* ☐☐☐☐ *ALPHA CODE* ☐☐☐☐

37. Resuscitation: ☐ No ☐ Yes If Yes, Date: ☐☐ *month* ☐☐ *day* ☐☐☐☐ *year*

38. Inotropes: ☐ No ☐ Yes

39. Status of Index CABG Procedure:
☐ Elective ☐ Emergent *
☐ Urgent ☐ Emergent salvage *
*(*NOTE: 'Emergent' and 'Emergent salvage' are exclusion criteria.)*

<u>VALVE DISEASE BY ECHO TYPE</u> (*Note: 'Moderate' and 'Severe' are exclusion criteria.)*

40. Aortic regurgitation (Insufficiency)
☐ None ☐ Trivial ☐ Mild (1+) ☐ Moderate (2-3+) ☐ Severe (4+)

41. Aortic stenosis
☐ None ☐ Trivial ☐ Mild (1+) ☐ Moderate (2-3+) ☐ Severe (4+)

42. Mitral regurgitation (Insufficiency)
☐ None ☐ Trivial ☐ Mild (1+) ☐ Moderate (2-3+) ☐ Severe (4+)

43. Mitral stenosis
☐ None ☐ Trivial ☐ Mild (1+) ☐ Moderate (2-3+) ☐ Severe (4+)

44. Tricuspid regurgitation (Insufficiency)
☐ None ☐ Trivial ☐ Mild (1+) ☐ Moderate (2-3+) ☐ Severe (4+)

<u>CORONARY STATUS</u>

45. Coronary angiography performed: ☐☐ *month* ☐☐ *day* ☐☐☐☐ *year*

46. Dominant coronary system: ☐ Right ☐ Left ☐ Co-dominant

47. Left main $\geq$ 50% stenosis: ☐ No ☐ Yes

48. Diseased territories:
☐ Single vessel disease ☐ Double vessel disease ☐ Triple vessel disease

49. Syntax Score: ☐☐ *(www.syntaxscore.com)*

SITE NO. ☐☐☐ *SUBJECT ID.* ☐☐☐☐ *ALPHA CODE* ☐☐☐☐

**CASS SITE CODES**					
1 = Prox RCA	6 = 1st RPL	11 =LMCA	16 = 2nd Diag	21 = 2nd OM	26 = 3rd LPL
2 = Mid RCA	7 = 2nd RPL	12 = Prox LAD	17 = 1st Septal	22 = 3rd OM	27 = LPDA
3 = Dist RCA	8 = 3rd RPL	13 = Mid LAD	18 = Prox Cx	23 = Dist Cx	28 = RAMUS
4 = RPDA	9 = Inferior Septal	14 = Dist LAD	19 = Mid Cx	24 = 1st LPL	29 = 3rd Diag
5 = RPAV	10 = Acute Marginal	15 = 1st Diag	20 = 1st OM	25 = 2nd LPL	30 = Unknown

CORONARY MAP:

	**a.**	**b.**	**c.**	**d.**	**e.**	**f.**
50. CASS Site	☐☐	☐☐	☐☐	☐☐	☐☐	☐☐
51. Stenosis %	☐☐☐	☐☐☐	☐☐☐	☐☐☐	☐☐☐	☐☐☐
52. Vessel Diameter (mm)	☐.☐	☐.☐	☐.☐	☐.☐	☐.☐	☐.☐
53. PCI	☐ No<br>☐ Yes	☐ No<br>☐ Yes	☐ No<br>☐ Yes	☐ No<br>☐ Yes	☐ No<br>☐ Yes	☐ No<br>☐ Yes
54. Intent to bypass	☐ No<br>☐ Yes	☐ No<br>☐ Yes	☐ No<br>☐ Yes	☐ No<br>☐ Yes	☐ No<br>☐ Yes	☐ No<br>☐ Yes

a. If yes, Type of conduit to be used

**a.**	**b.**	**c.**	**d.**	**e.**	**f.**
☐ LIMA<br>☐ RIMA<br>☐ Radial Artery<br>☐ Venous<br>☐ Other<br>__________	☐ LIMA<br>☐ RIMA<br>☐ Radial Artery<br>☐ Venous<br>☐ Other<br>__________	☐ LIMA<br>☐ RIMA<br>☐ Radial Artery<br>☐ Venous<br>☐ Other<br>__________	☐ LIMA<br>☐ RIMA<br>☐ Radial Artery<br>☐ Venous<br>☐ Other<br>__________	☐ LIMA<br>☐ RIMA<br>☐ Radial Artery<br>☐ Venous<br>☐ Other<br>__________	☐ LIMA<br>☐ RIMA<br>☐ Radial Artery<br>☐ Venous<br>☐ Other<br>__________

SITE NO.	*SUBJECT ID.*	*ALPHA CODE*
☐☐☐	☐☐☐☐	☐☐☐☐

PHYSICAL ASSESSMENT

55. Height: ☐☐☐ cm

56. Weight: ☐☐☐ . ☐ kg

57. Body Surface Area (Mosteller Formula): ☐ . ☐☐ m^2

58. Body Mass Index (NHLBI Calculator): ☐☐ . ☐ kg/m^2

59. Ankle-Brachial Index (lower ABI) – **RIGHT**: (research procedure) ☐ . ☐☐

60. Ankle-Brachial Index (lower ABI) – **LEFT**: (research procedure) ☐ . ☐☐

CLINICAL LABORATORY DATA (If no results available, leave blank)

			Date:		
61. HbA1c	☐☐ . ☐	%	☐☐ *month*	☐☐ *day*	☐☐☐☐ *year*
62. Potassium ☐ Serum ☐ plasma	☐ . ☐	mEq/L	☐☐ *month*	☐☐ *day*	☐☐☐☐ *year*
63. Serum Creatinine	☐ . ☐	mg/dL	☐☐ *month*	☐☐ *day*	☐☐☐☐ *year*
64. Plasma Creatinine	☐ . ☐	mg/dL	☐☐ *month*	☐☐ *day*	☐☐☐☐ *year*
65. eGFR (IDMS) < 60?	☐ No ☐ Yes		☐☐ *month*	☐☐ *day*	☐☐☐☐ *year*
66. C-Reactive Protein (hs-CRP)	☐☐ . ☐	mg/dL	☐☐ *month*	☐☐ *day*	☐☐☐☐ *year*
67. White Blood Cell Count (WBC)	☐☐ . ☐☐	$10^3/mm^3$	☐☐ *month*	☐☐ *day*	☐☐☐☐ *year*
68. Hematocrit (HCT)	☐☐ . ☐	%	☐☐ *month*	☐☐ *day*	☐☐☐☐ *year*

SITE NO.	*SUBJECT ID.*	*ALPHA CODE*
☐☐☐	☐☐☐☐	☐☐☐☐

69. Total Cholesterol (fasting TC) ☐☐☐ mg/dL Date: ☐☐ *month* ☐☐ *day* ☐☐☐☐ *year*

70. High Density Lipoproteins (fasting HDL) ☐☐☐ mg/dL Date: ☐☐ *month* ☐☐ *day* ☐☐☐☐ *year*

71. Low Density Lipoproteins (fasting LDL) ☐☐☐ mg/dL Date: ☐☐ *month* ☐☐ *day* ☐☐☐☐ *year*

72. Triglycerides (fasting TRG) ☐☐☐ mg/dL Date: ☐☐ *month* ☐☐ *day* ☐☐☐☐ *year*

30 DAY MORTALITY SURGICAL RISK ASSESSMENT

73. VASQIP Patient Risk Calculation (Online Calculator): ☐ . ☐ %

74. STS Risk of Mortality (Online STS Risk Calculator): ☐ . ☐☐ %

VENOUS CLINICAL SEVERITY SCORE

75. Pain or other discomfort **(i.e., aching, heaviness, fatigue, soreness, burning)**

☐ 0= None

☐ 1= Mild – Occasional pain or discomfort that does not restrict regular daily activities

☐ 2= Moderate – Daily pain or discomfort, interferes with, but does not prevent, regular daily activities

☐ 3= Severe – Daily pain or discomfort that limits most regular daily activities

76. Varicose Veins **(>3mm in diameter, use standing position for assessment)**

☐ 0= None

☐ 1= Mild – Few, scattered, varicosities that are confined to branch veins or clusters

☐ 2= Moderate – Multiple varicosities that are confined to the calf or the thigh

☐ 3= Severe – Multiple varicosities that involve both the calf and the thigh

SITE NO.	SUBJECT ID.	ALPHA CODE

77. Venous Edema **(exam may be supplemented by asking patient about edema)**

☐ 0= None

☐ 1= Mild – Edema is limited to the foot and ankle

☐ 2= Moderate – Edema extends above the ankle but below the knee

☐ 3= Severe – Edema extends to the knee or above

78. Skin Pigmentation **(color changes of venous origin, not from other chronic diseases)**

☐ 0= None

☐ 1= Mild – Pigmentation that is limited to the peri malleolar area

☐ 2= Moderate – Diffuse pigmentation that involves the lower third of the calf

☐ 3= Severe – Diffuse pigmentation that involves more than the lower third of the calf

79. Inflammation **(erythema, cellulitis, venous eczema, or dermatitis)**

☐ 0= None

☐ 1= Mild – Inflammation that is limited to the peri malleolar area

☐ 2= Moderate – Inflammation that involves the lower third of the calf

☐ 3= Severe – Inflammation that involves more than the lower third of the calf

80. Induration **(refers to skin and subcutaneous changes)**

☐ 0= None

☐ 1= Mild – Iduration that is limited to the peri malleolar area

☐ 2= Moderate – Iduration that involves the lower third of the calf

☐ 3= Severe – Induration that involves more than the lower third of the calf

81. Active Ulcer Number **(count the number of active leg ulcers)**

☐ 0= None (Skip questions 82-84, calculate total score and sign form)

☐ 1= Mild – 1 ulcer

☐ 2= Moderate – 2 ulcers

☐ 3= Severe – $\geq$3 ulcers

SITE NO.	SUBJECT ID.	ALPHA CODE
☐☐☐	☐☐☐☐	☐☐☐☐

82. Active Ulcer Duration **(if there is at least one active ulcer)**

☐ 1= Mild – Ulceration present <3 months

☐ 2= Moderate – Ulceration present 3-12 months

☐ 3= Severe – Ulceration present > 12 months

83. Active Ulcer Size **(if there is at least one active ulcer)**

☐ 1= Mild – Ulcer <2 cm in diameter

☐ 2= Moderate – Ulcer 2-6 cm in diameter

☐ 3= Severe – Ulcer >6 cm in diameter

84. Compression Therapy **(if there is at least one active ulcer)**

☐ 0= None

☐ 1= Mild – Intermittent use

☐ 2= Moderate – Wears stockings most days

☐ 3= Severe – Full compliance: stockings

85. **TOTAL VENOUS CLINICAL SEVERITY SCORE** (add totals for Questions 75-84) **=** ☐☐

S.I. Signature ______________________________________ Date ____________________

VISIT NO.	*SITE NO.*	*SUBJECT ID*	*ALPHA CODE*	*DATE OF ASSESSMENT*
☐☐	☐☐☐	☐☐☐☐	☐☐☐☐	☐☐ *Month* ☐☐ *Day* ☐☐☐☐ *Year*

FORM 03 - THE SEATTLE ANGINA QUESTIONNAIRE

1. The following is a list of activities that people often do during the week. Although for some people with several medical problems it is difficult to determine what it is that limits them, please go over the activities listed below and indicate how much limitation you have had **due to chest pain, chest tightness, or angina** over the past 4 weeks. Mark an X in one box on each line.

ACTIVITIES	Extremely Limited	Quite a Bit Limited	Moderately Limited	Slightly Limited	Not at all Limited	Limited for other reasons or did not do the activity
a. Dressing yourself	☐	☐	☐	☐	☐	☐
b. Walking indoors on level ground	☐	☐	☐	☐	☐	☐
c. Showering	☐	☐	☐	☐	☐	☐
d. Climbing a hill or a flight of stairs without stopping	☐	☐	☐	☐	☐	☐
e. Gardening, vacuuming, or carrying groceries	☐	☐	☐	☐	☐	☐
f. Walking more than a block at a brisk pace	☐	☐	☐	☐	☐	☐
g. Running or jogging	☐	☐	☐	☐	☐	☐
h. Lifting or moving heavy objects (e.g. furniture, children)	☐	☐	☐	☐	☐	☐
i. Participating in strenuous sports (e.g. swimming, tennis)	☐	☐	☐	☐	☐	☐

VISIT NO. ☐☐ ***SITE NO.*** ☐☐☐ ***SUBJECT ID*** ☐☐☐☐ ***ALPHA CODE*** ☐☐☐☐

2. Compared with 4 weeks ago, how often do you have chest pain, chest tightness, or angina when doing your most strenuous activities? I have had chest pain, chest tightness, or angina

- Much more often .... ☐
- Slightly more often .... ☐
- About the same .... ☐
- Slightly less often .... ☐
- Much less often .... ☐
- I have had no chest pain over the last 4 weeks .... ☐

3. Over the past 4 weeks, on average, how many times have you had chest pain, chest tightness, or angina? I have had chest pain, chest tightness, or angina

- 4 or more times per day .... ☐
- 1-3 times per day .... ☐
- 3 or more times per week but not every day........ ☐
- 1-2 times per week........ ☐
- Less than once a week .... ☐
- None over the past 4 weeks .... ☐

4. Over the past 4 weeks, on average, how many times have you had to take nitroglycerin (nitroglycerin tablets or spray) for your chest pain, chest tightness, or angina? I have taken nitroglycerin

- 4 or more times per day .... ☐
- 1-3 times per day .... ☐
- 3 or more times per week but not every day........ ☐
- 1-2 times per week........ ☐
- Less than once a week .... ☐
- None over the past 4 weeks .... ☐

VISIT NO.	***SITE NO.***	***SUBJECT ID***	***ALPHA CODE***
☐☐	☐☐☐	☐☐☐☐	☐☐☐☐

5. How bothersome is it for you to take your pills for chest pain, chest tightness or angina as prescribed?

   - Extremely bothersome ☐
   - Quite a bit bothersome ☐
   - Moderately bothersome ☐
   - Slightly bothersome ☐
   - Not bothersome at all ☐
   - My doctor has not prescribed pills ☐

6. How satisfied are you that everything possible is being done to treat your chest pain, chest tightness, or angina?

   - Not satisfied at all ☐
   - Mostly dissatisfied ☐
   - Somewhat satisfied ☐
   - Mostly satisfied ☐
   - Completely satisfied ☐

7. How satisfied are you with the explanations your doctor has given you about your chest pain, chest tightness, or angina?

   - Not satisfied at all ☐
   - Mostly dissatisfied ☐
   - Somewhat satisfied ☐
   - Mostly satisfied ☐
   - Completely satisfied ☐

VISIT NO.	*SITE NO.*	*SUBJECT ID*	*ALPHA CODE*
☐☐	☐☐☐	☐☐☐☐	☐☐☐☐

8. Overall, how satisfied are you with the current treatment of your chest pain, chest tightness, or angina?

   Not satisfied at all .... ☐
   Mostly dissatisfied .... ☐
   Somewhat satisfied .... ☐
   Mostly satisfied .... ☐
   Completely satisfied .... ☐

9. Over the <u>past 4 weeks</u>, how much has your chest pain, chest tightness, or angina limited your enjoyment of life?

   It has extremely limited my enjoyment of life .... ☐
   It has limited my enjoyment of life quite a bit .... ☐
   It has moderately limited my enjoyment of life .... ☐
   It has slightly limited my enjoyment of life .... ☐
   It has not limited my enjoyment of life at all .... ☐

10. If you had to spend the rest of your life with your chest pain, chest tightness, or angina the way it is right now, how would you feel about this?

   Not satisfied at all .... ☐
   Mostly dissatisfied .... ☐
   Somewhat satisfied .... ☐
   Mostly satisfied .... ☐
   Completely satisfied .... ☐

VISIT NO.	*SITE NO.*	*SUBJECT ID*	*ALPHA CODE*

11. How often do you think or worry that you may have a heart attack or die suddenly?

- I can't stop thinking or worrying about it .... ☐
- I often think or worry about it...................................... ☐
- I occasionally think or worry about it .... ☐
- I rarely think or worry about it...................................... ☐
- I never think or worry about it...................................... ☐

VISIT NO.	***SITE NO.***	***SUBJECT ID***	***ALPHA CODE***	***DATE OF ASSESSMENT***
☐☐	☐☐☐	☐☐☐☐	☐☐☐☐	☐☐ ☐☐ ☐☐☐☐
				Month *Day* *Year*

FORM 04 – THE VETERANS RAND 12 ITEM HEALTH SURVEY (VR 12)

INSTRUCTIONS: This questionnaire asks for your views about your health. This information will help keep track of how you feel and how well you are able to do your usual activities. For each of the following questions, please mark an 'X' in the one box that best describes your answer.

1. In general, would you say your health is:

Excellent	Very good	Good	Fair	Poor
☐	☐	☐	☐	☐

2. The following questions are about activities you might do during a typical day. Does your health now limit you in these activities? If so, how much?

	Yes, Limited A Lot	Yes, Limited A Little	No, Not Limited At All
a. **Moderate activities**, such as moving a table, pushing a vacuum cleaner, bowling, or playing golf?	☐	☐	☐
b. Climbing **several** flights of stairs?	☐	☐	☐

3. During the past 4 weeks, have you had any of the following problems with your work or other regular daily activities as a result of your physical health?

	No, None of the Time	Yes, a Little of the Time	Yes, Some of the Time	Yes, Most of the Time	Yes, All of the Time
a. **Accomplished less** than you would like.	☐	☐	☐	☐	☐
b. Were limited in the **kind** of work or other activities.	☐	☐	☐	☐	☐

VISIT NO.	*SITE NO.*	*SUBJECT ID*	*ALPHA CODE*
☐☐	☐☐☐	☐☐☐☐	☐☐☐☐

4. During the <u>past 4 weeks</u>, have you had any of the following problems with your work or other regular daily activities <u>as a result of any emotional problems</u> (such as feeling depressed or anxious)?

	No, None of the Time	Yes, a Little of the Time	Yes, Some of the Time	Yes, Most of the Time	Yes, All of the Time
a. **Accomplished less** than you would like.	☐	☐	☐	☐	☐
b. Didn't do work or other activities as **carefully** as usual.	☐	☐	☐	☐	☐

5. During the <u>past 4 weeks</u>, how much did <u>pain</u> interfere with your normal work (including both work outside the home and housework)?

Not at all	A little bit	Moderately	Quite a bit	Extremely
☐	☐	☐	☐	☐

These questions are about how you feel and how things have been with you <u>during the past 4 weeks</u>. For each question, please give the one answer that comes closest to the way you have been feeling.

6. How much of the time during the <u>past 4 weeks</u>:

	All of the Time	Most of the Time	A Good Bit of the Time	Some of the Time	A Little of the Time	None of the Time
a. Have you felt **calm and peaceful**?	☐	☐	☐	☐	☐	☐
b. Did you have a **lot of energy**?	☐	☐	☐	☐	☐	☐
c. Have you felt **downhearted and blue**?	☐	☐	☐	☐	☐	☐

VISIT NO.	*SITE NO.*	*SUBJECT ID*	*ALPHA CODE*
☐☐	☐☐☐	☐☐☐☐	☐☐☐☐

7. During the past 4 weeks, how much of the time has your physical health or emotional problems interfered with your social activities (like visiting with friends, relatives, etc.)?

All of the time	Most of the time	Some of the time	A little of the time	None of the time
☐	☐	☐	☐	☐

Now, we'd like to ask you some questions about how your health may have changed.

8. Compared to one year ago, how would you rate your **physical health** in general now?

Much better	Slightly better	About the same	Slightly worse	Much worse
☐	☐	☐	☐	☐

9. Compared to one year ago, how would you rate your **emotional problems** (such as feeling anxious, depressed or irritable) now?

Much better	Slightly better	About the same	Slightly worse	Much worse
☐	☐	☐	☐	☐

SITE NO. [ ][ ][ ] SUBJECT ID. [ ][ ][ ][ ] ALPHA CODE [ ][ ][ ][ ]

FORM 05 – INTRAOPERATIVE DATA COLLECTION

Complete Form BEFORE Leaving the Operating Room

KEY INTRAOPERATIVE TIMEPOINTS (EVH and OVH CASES) *(Note: use Military time)*

1. Date of Surgery: [ ][ ] *month* [ ][ ] *day* [ ][ ][ ][ ] *year*

2. Patient arrives in operating room: .... [ ][ ] : [ ][ ]

3. Chest skin incision start time: .... [ ][ ] : [ ][ ]

4. Leg skin incision start time: .... [ ][ ] : [ ][ ]

5. Vein COMPLETELY REMOVED from leg tunnel/wound, placed in solution: .... [ ][ ] : [ ][ ]

6. Vein FULLY PREPPED and ready for use as graft: .... [ ][ ] : [ ][ ]

7. Vein handed up to surgeon for suturing to graft site: .... [ ][ ] : [ ][ ]

8. Cardiopulmonary bypass initiated (on-pump): .... [ ][ ] : [ ][ ]

9. Aortic cross-clamp applied: .... [ ][ ] : [ ][ ]

10. Aortic cross-clamp removed: .... [ ][ ] : [ ][ ]

11. Cardiopulmonary bypass discontinued (off-pump): .... [ ][ ] : [ ][ ]

12. Surgery end-time: .... [ ][ ] : [ ][ ]

13. Patient departs from operating room: .... [ ][ ] : [ ][ ]

SITE NO.	SUBJECT ID.	ALPHA CODE
☐☐☐	☐☐☐☐	☐☐☐☐

PRE-PROCEDURE DATA (EVH and OVH)

14. Vein mapping performed: ☐ No ☐ Yes

   a. If yes, performed by: ☐ Technician ☐ Mid-Level Provider ☐ Surgeon

   b. if yes, when: ☐ PREOPERATIVELY ☐ INTRAOPERATIVELY

   c. if Preoperatively, when: ☐ Prior to Day of Surgery (DOS)

   ☐ DOS – Prior to Induction of Anesthesia

   ☐ DOS – Following Induction of Anesthesia

15. Pre-op IV heparin: ☐ No ☐ Yes (If No, proceed to question 16)

   a. If discontinued, date: ☐☐ ☐☐ ☐☐☐☐
   month *day* *year*

   b. If discontinued, time: ☐☐ : ☐☐ (military time)

16. Baseline activated clotting time (ACT): ☐☐☐ seconds

17. Central venous pressure (CVP): ☐☐ mmHg

18. $PaCO_2$: ☐☐ mmHg

19. $ETCO_2$: ☐☐ mmHg

20. FiO_2: ☐ . ☐☐ %

21. PO_2: ☐☐☐ mmHg

22. EVH initiated: ☐ No ☐ Yes ***(If, NO skip to Q32)***

SITE NO.	SUBJECT ID.	ALPHA CODE
☐☐☐	☐☐☐☐	☐☐☐☐

ENDOSCOPIC HARVEST DATA

23. Prophylactic IV heparin bolus **IMMEDIATELY PRIOR TO EVH**: ☐ No ☐ Yes

   a. If YES, dosage amount: ☐☐☐☐

   b. If NO, provide reason:

   ☐ ACT already therapeutic ☐ Pre-op Plavix use

   ☐ Thrombocytopenia ☐ Other, specify________________________

24. EVH system:

   ☐ VasoView **with** HemoPro I (Maquet) ☐ VirtuoSaph (Terumo)

   ☐ VasoView **with** HemoPro II (Maquet) ☐ VirtuoSaph Plus (Terumo)

   ☐ VasoView **with** Bipolar (Maquet) ☐ Other, Specify____________________

   a. If HemoPro, give setting: ☐ thermal units

   b. If Bipolar, give setting: ☐☐ watts

25. CO_2 flow rate: ☐☐ liters/min

26. CO_2 max pressure: ☐☐ mmHg

27. $PaCO_2$ (mid-procedure): ☐☐ mmHg

28. $ETCO_2$ (mid-procedure): ☐☐ mmHg

29. FiO_2 (mid-procedure): ☐ . ☐☐ %

30. PO_2 (mid-procedure): ☐☐☐ mmHg

31. Conversion to open harvest procedure: ☐ No ☐ Yes ***(If, NO than Q54=0)***

OPEN HARVEST DATA (Randomization and Conversion)

32. OVH performed: ☐ No ☐ Yes, for randomization ☐ Yes, for conversion

SITE NO. ☐☐☐ *SUBJECT ID.* ☐☐☐☐ *ALPHA CODE* ☐☐☐☐

33. Type of incision:

☐ Single/long

☐ Multiple/bridging

☐ Other, specify ____________________________

34. Number of incision(s): ☐☐

35. Length of cumulative incision(s): ☐☐☐ cm *(Note: add total length of incisions – not the bridging)*

36. Flap created: ☐ No ☐ Yes

37. Device used: ☐ No ☐ Yes

a. If YES, type:

☐ Storz

☐ Direct View Retractor

☐ Other, specify ____________________________

38. $PaCO_2$ (mid-procedure): ☐☐ mmHg

39. $ETCO_2$ (mid-procedure): ☐☐ mmHg

40. FiO_2 (mid-procedure): ☐ . ☐☐ %

41. PO_2 (mid-procedure): ☐☐☐ mmHg

POST-HARVEST DATA (EVH and OVH CASES)

42. Vein storage solution composition:

☐ GALA

☐ NSS

☐ Heparinized blood with papaverine

☐ Heparinized blood

☐ Other, specify________________________

43. Pressure-limiting syringe used to distend vein: ☐ No ☐ Yes

SITE NO.	*SUBJECT ID.*	*ALPHA CODE*
☐☐☐	☐☐☐☐	☐☐☐☐

44. Total # repairs to EVH vein segment: ☐☐

45. Total # repairs to OVH vein segment: ☐☐

46. $PaCO_2$: ☐☐ mmHg

47. $ETCO_2$: ☐☐ mmHg

48. FiO_2: ☐ . ☐☐ %

49. PO_2: ☐☐☐ mmHg

50. Intra-Aortic Balloon Pump Inserted?

☐ No ☐ Yes, INTRA-operatively

☐ Yes, PRE-operatively ☐ Yes, POST-operatively

SITE NO.	*SUBJECT ID.*	*ALPHA CODE*
☐☐☐	☐☐☐☐	☐☐☐☐

PRIMARY VEIN HARVESTER TO COMPLETE PROCEDURE MAP BELOW FOR ALL CASES (EVH and OVH)

*Primary vein harvester is the person who spends the greatest amount of time performing the vein harvest procedure; if two people perform equal harvest time then identify the harvester who initiated the procedure.

GSV=Greater Saphenous Vein

COMPLETE ENTIRE COLUMN FOR EACH VEIN GRAFT HARVESTED ON THE RIGHT:

VEIN HARVEST PROCEDURE MAP	RIGHT		
	**a. GSV Thigh**	**b. GSV Calf**	**c. Other Vein**
51. Vein harvest site:	☐ No<br>☐ Yes	☐ No<br>☐ Yes	☐ No<br>☐ Yes
52. Vein harvester:	☐<br>If 5, _______________	☐<br>If 5, _______________	☐<br>If 5, _______________
53. Vein harvest method	☐ EVH<br>☐ OVH	☐ EVH<br>☐ OVH	☐ EVH<br>☐ OVH
54. Conversion from EVH to OVH:	☐<br>If 9, _______________	☐<br>If 9, _______________	☐<br>If 9, _______________
55. Harvest site closure by:	☐<br>If 6, _______________	☐<br>If 6, _______________	☐<br>If 6, _______________
56. Skin closure technique:	☐<br>If 6, _______________	☐<br>If 6, _______________	☐<br>If 6, _______________
57. Drain use:	☐ No<br>☐ Yes	☐ No<br>☐ Yes	☐ No<br>☐ Yes
58. Hematoma:	☐ No<br>☐ Yes	☐ No<br>☐ Yes	☐ No<br>☐ Yes

SITE NO.	*SUBJECT ID.*	*ALPHA CODE*
☐☐☐	☐☐☐☐	☐☐☐☐

COMPLETE ENTIRE COLUMN FOR EACH VEIN GRAFT HARVESTED ON THE LEFT:

**VEIN HARVEST PROCEDURE MAP**	**LEFT**		
	**d. GSV Thigh**	**e. GSV Calf**	**f. Other Vein**
51. Vein harvest site:	☐ No<br>☐ Yes	☐ No<br>☐ Yes	☐ No<br>☐ Yes
52. Vein harvester:	☐<br>If 5, _______________	☐<br>If 5, _______________	☐<br>If 5, _______________
53. Vein harvest method	☐ EVH<br>☐ OVH	☐ EVH<br>☐ OVH	☐ EVH<br>☐ OVH
54. Conversion from EVH to OVH:	☐<br>If 9, _______________	☐<br>If 9, _______________	☐<br>If 9, _______________
55. Harvest site closure by:	☐<br>If 6, _______________	☐<br>If 6, _______________	☐<br>If 6, _______________
56. Skin closure technique:	☐<br>If 6, _______________	☐<br>If 6, _______________	☐<br>If 6, _______________
57. Drain use:	☐ No<br>☐ Yes	☐ No<br>☐ Yes	☐ No<br>☐ Yes
58. Hematoma:	☐ No<br>☐ Yes	☐ No<br>☐ Yes	☐ No<br>☐ Yes

SITE NO.	SUBJECT ID.	ALPHA CODE
☐☐☐	☐☐☐☐	☐☐☐☐

STUDY SITE SURGEON TO COMPLETE CABG PROCEDURE MAP BELOW FOR ALL CASES (EVH and OVH) *See Codes*

COMPLETE AN ENTIRE COLUMN FOR EACH BYPASS (a.= 1st bypass constructed, b.=2nd bypass, c.=3rd bypass).

GRAFT	a.	b.	c.
59. Bypass graft completed as indicated on Form 2, Q54	☐ No ☐ Yes<br>If No, provide reason<br>☐<br>If 4, ________________	☐ No ☐ Yes<br>If No, provide reason<br>☐<br>If 4, ________________	☐ No ☐ Yes<br>If No, provide reason<br>☐<br>If 4, ________________
60. Conduit source:	☐<br>If 4 or 9,<br>_______________	☐<br>If 4 or 9,<br>_______________	☐<br>If 4 or 9,<br>_______________
61. Harvest method:	☐	☐	☐
62. Conduit quality:	☐<br>If 3 or 8,<br>_______________	☐<br>If 3 or 8,<br>_______________	☐<br>If 3 or 8,<br>_______________
63. Conduit size: (mm)	☐☐	☐☐	☐☐
64. Proximal site:	☐	☐	☐
65. CASS site:	☐☐	☐☐	☐☐
66. Distal target size:	☐	☐	☐
67. Distal disease:	☐	☐	☐
68. Distal anastomosis:	☐ Attending Surgeon<br>☐ Resident/Fellow	☐ Attending Surgeon<br>☐ Resident/Fellow	☐ Attending Surgeon<br>☐ Resident/Fellow

SITE NO.	SUBJECT ID.	ALPHA CODE
☐☐☐	☐☐☐☐	☐☐☐☐

COMPLETE AN ENTIRE COLUMN FOR EACH BYPASS (d.= 4th bypass constructed; e.= 5th bypass; f.= 6th bypass)

GRAFT	d.	e.	f.
59. Bypass graft completed as indicated on Form 2, Q54	☐ No ☐ Yes<br>If No, provide reason<br>☐<br>If 4, ________________	☐ No ☐ Yes<br>If No, provide reason<br>☐<br>If 4, ________________	☐ No ☐ Yes<br>If No, provide reason<br>☐<br>If 4, ________________
60. Conduit source:	☐<br>If 4 or 9,<br>________________	☐<br>If 4 or 9,<br>________________	☐<br>If 4 or 9,<br>________________
61. Harvest method:	☐	☐	☐
62. Conduit quality:	☐<br>If 3 or 8,<br>________________	☐<br>If 3 or 8,<br>________________	☐<br>If 3 or ,<br>________________
63. Conduit size: (mm)	☐☐	☐☐	☐☐
64. Proximal site:	☐	☐	☐
65. CASS site:	☐☐	☐☐	☐☐
66. Distal target size:	☐	☐	☐
67. Distal disease:	☐	☐	☐
68. Distal anastomosis:	☐ Attending Surgeon<br>☐ Resident/Fellow	☐ Attending Surgeon<br>☐ Resident/Fellow	☐ Attending Surgeon<br>☐ Resident/Fellow

S.I. Signature ____ Date ____________________

VEIN HARVEST CODES

**52. Primary Vein Harvester***	**53. Method of Vein Harvest:**	**54. Conversion from EVH to OVH r/t:**
1= PA-C<br>2= CRNP/CRNFA<br>3= Attending Surgeon<br>4= Resident/Fellow<br>5= other	EVH= Endoscopic<br>OVH= Open	0= NOT APPLICABLE (if 0 then #31=NO)<br>1= Bleeding (if 1-9 then #31 =YES)<br>2= Injury to SVG<br>3= Unacceptable EVH procedure time<br>4= Insufficient amount of usable vein from EVH procedure<br>5= Unanticipated graft needed requiring additional vein taken open<br>6= Harvester unable to locate vein<br>7= Equipment issue (i.e. contamination, failure, availability)<br>8= Patient becomes unstable<br>9= other
**55. Harvest Site Closure By:**	**56. Skin Closure Technique:**	
1= Primary Vein Harvester (#52)<br>2= other PA-C<br>3= other CRNP/CRNFA<br>4= other Attending surgeon<br>5= other Resident/Fellow<br>6= other	1= Subcuticular suture<br>2= Staples<br>3= Steristrips<br>4= Dermabond<br>5= Combination of above<br>6= other	

*Primary vein harvester is the person who spends the greatest amount of time performing the vein harvest procedure; if two people perform equal harvest time then identify the harvester who initiated the procedure.

CABG CODES

**59. BYPASS NOT COMPLETED AS INDICATED AT BASELINE (Form 2)**	**60. CONDUIT SOURCE**		**61. HARVEST METHOD**	**62. QUALITY OF VEIN CONDUIT USED FOR GRAFTING (if Arterial, leave blank)**
1= Target too Small<br>2= Diffuse Disease<br>3= Inadequate Conduit<br>4= other	**ARTERIAL:**<br>1= LIMA<br>2= RIMA<br>3= Radial<br>4= other	**VENOUS:**<br>5= Right Thigh<br>6= Right Calf<br>7= Left Thigh<br>8= Left Calf<br>9= other	1= Arterial<br>2= EVH<br>3= OVH	**DOES NOT INCLUDE DISCARDED VEIN**<br>1= Good (ideal vein with uniform caliber, few branches)<br>2= Intermediate (usable vein but sclerotic, non-distensible)<br>3= Intermediate d/t OTHER specify ____________________________<br>4= Poor d/t THIN VEIN<br>5= Poor d/t VARICOSE VEIN<br>6= Poor d/t DE-SEROSALIZED WALL from MECHANICAL INJURY<br>7= Poor d/t THERMAL INJURY<br>8= Poor d/t OTHER specify____________________________________
				**63. VEIN CONDUIT SIZE (if Arterial, leave blank)**<br>Estimate to closest mm **after vein is distended**.

**64. PROXIMAL SITE**	**66. DISTAL TARGET SIZE**	**67. DISTAL TARGET DISEASE**	**68. DISTAL TARGET ANASTOMOSIS**
1= Ascending Aorta<br>2= Pedical IMA (In-situ)<br>3= LIMA<br>4= RIMA<br>5= SVG	1= <1.5mm<br>2= 1.5-2.0mm<br>3= >2.0mm	0= None<br>1= Mild<br>2= Moderate<br>3= Severe<br>4= Endarterectomy	Who performed distal anastomosis?<br>Attending Surgeon<br>Resident/Fellow

65. CASS SITE (DISTAL TARGET)

1= Prox RCA	6= 1st RPL	11= LMCA	16= 2ndDiag	21= 2nd OM	26= 3rd LPL
2= Mid RCA	7= 2nd RPL	12= Prox LAD	17= 1stSeptal	22= 3rd OM	27= LPDA
3= Dist RCA	8= 3rd RPL	13= Mid LAD	18= Prox Cx	23= Dist Cx	28= RAMUS
4= RPDA	9= Inferior Septal	14= Dist LAD	19= Mid Cx	24= 1st LPL	29= 3rdDiag
5= RPAV	10= Acute Marginal	15=1stDiag	20= 1st OM	25= 2nd LPL	30= Unknown

SITE NO.	SUBJECT ID	ALPHA CODE	DATE FORM COMPLETED
			Month / Day / Year

FORM 06 – POSTOPERATIVE ASSESSMENTS

1. POST-OP DAY #1 .... month / day / year

2. ECG evidence of new pathologic Q waves or new LBBB: .... No ☐ Yes ☐

   If yes, obtain Cardiac Biomarkers of choice every 8 hours until downward trend is seen in level and **complete A, B, OR C below**.

   A. Peak Cardiac Troponin I

   1. Peak Cardiac Troponin I (ng/mL): .... ☐☐.☐☐
   2. Reference Range (ng/mL) .... ☐.☐☐ to ☐.☐☐

   B. Peak Cardiac Troponin T

   1. Peak Cardiac Troponin T (ng/mL): .... ☐☐.☐☐
   2. Reference Range (ng/mL) .... ☐.☐☐ to ☐.☐☐

   C. Peak CPK/MB Panel

   1. Peak Total CPK (U/L): .... ☐☐☐☐
   2. Total CPK Reference Range (U/L) .... ☐☐ to ☐☐☐

   AND

   3. Peak CK-MB (ng/mL): .... ☐☐.☐
   4. CK-MB Reference Range (ng/mL) .... ☐ to ☐.☐

   AND

   5. Peak CK-MB Index (%): .... ☐☐.☐
   6. CK-MB Index Reference Range (%) .... ☐.☐ to ☐.☐

3. ECG evidence of arrhythmia: .... No ☐ Yes ☐
   1. If yes, specify:
      - ☐ atrial fibrillation
      - ☐ atrial flutter
      - ☐ pacemaker
      - ☐ other, specify____________________

4. Chest tube drainage (cc): .... ☐☐☐☐

SITE NO.	***SUBJECT ID***	***ALPHA CODE***
☐☐☐	☐☐☐☐	☐☐☐☐

5. Leg incision drainage (cc) *(if applicable)*: .... ☐☐☐☐
6. Leg incision dressings *(number of changes)*: .... ☐☐
7. Blood/Products Transfusion: .... No ☐ Yes ☐
   1. If yes, check all that apply: ☐ PRBCs ☐ FFP ☐ Platelets ☐ Cryoprecipitate
8. Intra-aortic balloon pump: .... No ☐ Yes ☐
9. ECMO: .... No ☐ Yes ☐
10. Ventricular Assist Device: .... No ☐ Yes ☐

SITE NO. ☐☐☐ ***SUBJECT ID*** ☐☐☐☐ ***ALPHA CODE*** ☐☐☐☐

11. POST-OP DAY #2 .... ☐☐ *month* ☐☐ *day* ☐☐☐☐ *year*

12. ECG evidence of new pathologic Q waves or new LBBB: .... No ☐ Yes ☐

If yes, obtain Cardiac Biomarkers of choice every 8 hours until downward trend is seen in level and **complete A, B, OR C below**.

A. Peak Cardiac Troponin I

1. Peak Cardiac Troponin I (ng/mL): .... ☐☐.☐☐
2. Reference Range (ng/mL) .... ☐.☐☐ to ☐.☐☐

B. Peak Cardiac Troponin T

3. Peak Cardiac Troponin T (ng/mL): .... ☐☐.☐☐
4. Reference Range (ng/mL) .... ☐.☐☐ to ☐.☐☐

C. Peak CPK/MB Panel

7. Peak Total CPK (U/L): .... ☐☐☐☐
8. Total CPK Reference Range (U/L) .... ☐☐ to ☐☐☐

AND

9. Peak CK-MB (ng/mL): .... ☐☐.☐
10. CK-MB Reference Range (ng/mL) .... ☐ to ☐.☐

AND

11. Peak CK-MB Index (%): .... ☐☐.☐
12. CK-MB Index Reference Range (%) .... ☐.☐ to ☐.☐

13. ECG evidence of arrhythmia: .... No ☐ Yes ☐

1. If yes, specify: ☐ atrial fibrillation
   ☐ atrial flutter
   ☐ pacemaker
   ☐ other, specify___________________

14. Chest tube drainage (cc): .... ☐☐☐☐

SITE NO.	***SUBJECT ID***	***ALPHA CODE***
☐☐☐	☐☐☐☐	☐☐☐☐

15. Leg incision drainage (cc) *(if applicable)*: .... ☐☐☐☐
16. Leg incision dressings *(number of changes)*: .... ☐☐
17. Blood/Products Transfusion: .... No ☐ Yes ☐
    1. If yes, check all that apply:
       - ☐ PRBCs
       - ☐ FFP
       - ☐ Platelets
       - ☐ Cryoprecipitate
18. Intra-aortic balloon pump: .... No ☐ Yes ☐
19. ECMO: .... No ☐ Yes ☐
20. Ventricular Assist Device: .... No ☐ Yes ☐

S.I. Signature ____ Date ____________________

SITE NO.	SUBJECT ID	ALPHA CODE	DATE FORM COMPLETED
☐☐☐	☐☐☐☐	☐☐☐☐	☐☐ Month ☐☐ Day ☐☐☐☐ Year

FORM 07 – DISCHARGE ASSESSMENT

1. Date of Discharge .... ☐☐ month ☐☐ day ☐☐☐☐ year

2. Endotracheal tube extubation (final) > POD #2 No ☐ Yes ☐ → ☐☐ month ☐☐ day ☐☐☐☐ year

3. Inotropic Agents > POD #2:: .... No ☐ Yes ☐

   If yes, check all that apply:
   - ☐ Dopamine
   - ☐ Dobutamine
   - ☐ Epinephrine
   - ☐ Norepinephrine
   - ☐ Amrinone
   - ☐ Milrinone
   - ☐ other, specify____________________

4. Transfer order to step down (final) > POD #3 No ☐ Yes ☐ → ☐☐ month ☐☐ day ☐☐☐☐ year

5. Discharge order > 7 days of initial CABG No ☐ Yes ☐ → ☐☐ month ☐☐ day ☐☐☐☐ year

6. Creatinine (mg/dL) .... ☐ . ☐ ☐☐ month ☐☐ day ☐☐☐☐ year

7. Potassium (mEq/L) .... ☐ . ☐ ☐☐ month ☐☐ day ☐☐☐☐ year

8. White Blood Cell Count ($10^3/mm^3$) .... ☐☐ . ☐☐ ☐☐ month ☐☐ day ☐☐☐☐ year

9. Hematocrit (%) .... ☐☐ . ☐ ☐☐ month ☐☐ day ☐☐☐☐ year

S.I. Signature ____ Date ____________________

SITE NO.	SUBJECT ID	ALPHA CODE	DATE OF ASSESSMENT
☐☐☐	☐☐☐☐	☐☐☐☐	☐☐ Month ☐☐ Day ☐☐☐☐ Year

FORM 08 – LEG INCISION PAIN IMPACT QUESTIONNAIRE - DISCHARGE ASSESSMENT

1. How much leg incision pain have you had during the past week?

   None ☐ Very mild ☐ Mild ☐ Moderate ☐ Severe ☐ Very severe ☐

2. During the past week, how much did leg incision pain interfere with your normal activity?

   Not at all ☐ A little bit ☐ Moderately ☐ Quite a lot ☐ Extremely ☐

3. In the past week, how much of the time did leg incision pain interfere with your enjoyment of life?

   Never ☐ Rarely ☐ Sometimes ☐ Very often ☐ Always ☐

4. In the past week, how often did leg incision pain make simple tasks hard to complete?

   Never ☐ Rarely ☐ Sometimes ☐ Very often ☐ Always ☐

5. In the past week, how often were your leisure activities affected by your leg incision pain (including exercise and hobbies)?

   Never ☐ Rarely ☐ Sometimes ☐ Very often ☐ Always ☐

6. In the past week, how often did leg incision pain make you feel fed up and frustrated?

   Never ☐ Rarely ☐ Sometimes ☐ Very often ☐ Always ☐

SITE NO.	SUBJECT ID	ALPHA CODE	DATE OF ASSESSMENT		
☐☐☐	☐☐☐☐	☐☐☐☐	☐☐	☐☐	☐☐☐☐
			Month	Day	Year

FORM 09 – LEG INCISION PAIN IMPACT QUESTIONNAIRE

6-WEEK POSTOPERATIVE ASSESSMENT

1. How much leg incision pain have you had during the past 4 weeks?

   None ☐ Very mild ☐ Mild ☐ Moderate ☐ Severe ☐ Very severe ☐

2. During the past 4 weeks, how much did leg incision pain interfere with your normal activity?

   Not at all ☐ A little bit ☐ Moderately ☐ Quite a lot ☐ Extremely ☐

3. In the past 4 weeks, how much of the time did leg incision pain interfere with your enjoyment of life?

   Never ☐ Rarely ☐ Sometimes ☐ Very often ☐ Always ☐

4. In the past 4 weeks, how often did leg incision pain make simple tasks hard to complete?

   Never ☐ Rarely ☐ Sometimes ☐ Very often ☐ Always ☐

5. In the past 4 weeks, how often were your leisure activities affected by your leg incision pain (including exercise and hobbies)?

   Never ☐ Rarely ☐ Sometimes ☐ Very often ☐ Always ☐

6. In the past 4 weeks, how often did leg incision pain make you feel fed up and frustrated?

   Never ☐ Rarely ☐ Sometimes ☐ Very often ☐ Always ☐

SITE NO. ☐☐☐ ***SUBJECT ID*** ☐☐☐☐ ***ALPHA CODE*** ☐☐☐☐

FORM 10 – LEG INCISION ASSESSMENT

ASEPSIS SCORE CRITERIA

RATE BY EXTENT FOR 1 WEEK AT TIME OF DISCHARGE – Date of Assessment: ☐☐ (Month) ☐☐ (Day) ☐☐☐☐ (Year)

1. Serous exudates: ☐ 0 None ☐ 1 ☐ 2 ☐ 3 ☐ 4 ☐ 5 Severe

2. Erythema: ☐ 0 None ☐ 1 ☐ 2 ☐ 3 ☐ 4 ☐ 5 Severe

3. Purulent exudates: ☐ 0 None ☐ 1 ☐ 2 ☐ 3 ☐ 4 ☐ 5 ☐ 6 ☐ 7 ☐ 8 ☐ 9 ☐ 10 Severe

4. Separation of tissues: ☐ 0 None ☐ 1 ☐ 2 ☐ 3 ☐ 4 ☐ 5 ☐ 6 ☐ 7 ☐ 8 ☐ 9 ☐ 10 Severe

RATE BY EXTENT FOR 4-6 WEEKS AT TIME OF FOLLOW-UP VISIT – Since Discharge
Date of Assessment: ☐☐ (Month) ☐☐ (Day) ☐☐☐☐ (Year)

5. Antibiotics: .... ☐ No = 0 ☐ Yes=10

6. Drainage under local anesthetic: .... ☐ No = 0 ☐ Yes=5

7. Debridement under general anesthetic: .... ☐ No = 0 ☐ Yes=10

8. Bacterial isolation: .... ☐ No = 0 ☐ Yes=10

SITE NO.	*SUBJECT ID*	*ALPHA CODE*
☐☐☐	☐☐☐☐	☐☐☐☐

9. Hospital stay prolonged >14 days: *(if yes, complete Form 15 – SAE)* .... ☐ No = 0 ☐ Yes=5

10. Development of pus as an outpatient: .... ☐ No = 0 ☐ Yes=5

11. Visiting nurse visit to dress wound: .... ☐ No = 0 ☐ Yes=5

12. **Asepsis Score Total > 10 INDICATES WOUND INFECTION** **Score** ☐☐
(Total of Questions 1-11)

S.I. Signature ____ Date ___________________

SITE NO. ☐☐☐ *SUBJECT ID* ☐☐☐☐ *ALPHA CODE* ☐☐☐☐ *DATE OF VISIT* ☐☐ ☐☐ ☐☐☐☐
Month *Day* *Year*

FORM 11 – 6 WEEK MACE EVENT FORM

1. Has the subject died after the last contact? .... No ☐ Yes ☐
   (*If yes, complete Forms 14 and 15)*

   a. Date of death .... ☐☐ ☐☐ ☐☐☐☐
   Month *Day* *Year*

2. Did the subject have an acute myocardial infarction since the last contact? .... No ☐ Yes ☐
   (*If yes, complete form 15 and 19)*

   a. AMI Date .... ☐☐ ☐☐ ☐☐☐☐
   Month *Day* *Year*

3. Did the subject receive PCI since the last contact? .... No ☐ Yes ☐
   If yes,

   a. Procedure Date .... ☐☐ ☐☐ ☐☐☐☐
   Month *Day* *Year*

4. Did the subject receive CABG since the last contact? .... No ☐ Yes ☐
   If yes,

   a. Procedure Date .... ☐☐ ☐☐ ☐☐☐☐
   Month *Day* *Year*

For Q 1-4 answered yes, obtain appropriate medical records for required source documentation.

SITE NO.	***SUBJECT ID***	***ALPHA CODE***
☐☐☐	☐☐☐☐	☐☐☐☐

MEDICATIONS

5. Has the subject taken any of the following medications in since last contact?

a.	Beta blocker	No ☐	Yes ☐
b.	ACE/ARB Inhibitor	No ☐	Yes ☐
c.	Statin	No ☐	Yes ☐
d.	Non-Statin LLA	No ☐	Yes ☐
e.	Nitrate	No ☐	Yes ☐
f.	Calcium Channel Blocker	No ☐	Yes ☐
g.	Aspirin	No ☐	Yes ☐
h.	Plavix	No ☐	Yes ☐
i.	Warfarin	No ☐	Yes ☐

Form Completed By ____ Date __________________________

VISIT (Months) ***SITE NO.*** ***SUBJECT ID*** ***ALPHA CODE***

FORM 12 – PHONE CALL FOLLOW-UP FORM

1. Date of phone call *(or medical chart review)* .... Month / Day / Year

2. Subject contacted successfully? .... No ☐ Yes ☐

3. Did the subject die since the last contact? .... No ☐ Yes ☐
   If yes, (complete Forms 14 and 15)

   a. Date of death .... Month / Day / Year

4. Did the subject have an acute myocardial infarction since the last contact? .... No ☐ Yes ☐
   If yes, (complete form 15 and 19)

   a. AMI Date .... Month / Day / Year

5. Did the subject receive PCI since the last contact? .... No ☐ Yes ☐
   If yes,

   a. Procedure Date .... Month / Day / Year

6. Did the subject receive CABG since the last contact? .... No ☐ Yes ☐
   If yes,

   a. Procedure Date .... Month / Day / Year

For Q 3-6 answered yes, obtain appropriate medical records for required source documentation.

VISIT (Months)	*SITE NO.*	*SUBJECT ID*	*ALPHA CODE*
☐☐	☐☐☐	☐☐☐☐	☐☐☐☐

MEDICATIONS

7. Has the subject taken any of the following medications since last contact or review?

   a. Beta blocker .... No ☐ Yes ☐
   b. ACE/ARB Inhibitor .... No ☐ Yes ☐
   c. Statin .... No ☐ Yes ☐
   d. Non-Statin LLA .... No ☐ Yes ☐
   e. Nitrate .... No ☐ Yes ☐
   f. Calcium Channel Blocker .... No ☐ Yes ☐
   g. Aspirin .... No ☐ Yes ☐
   h. Plavix .... No ☐ Yes ☐
   i. Warfarin .... No ☐ Yes ☐

Form Completed By ____ Date ____________________________

VISIT NO.	*SITE NO.*	*SUBJECT ID*	*ALPHA CODE*	*DATE OF ASSESSMENT*		
☐☐	☐☐☐	☐☐☐☐	☐☐☐☐	☐☐	☐☐	☐☐☐☐
				Month	*Day*	*Year*

FORM 14 – STUDY COMPLETION/TERMINATION

1. Using the list below, mark an (x) in the box that best describes the subject's status at the end of the study (mark only one):

   ☐ Subject completed study

   ☐ Subject voluntarily withdrew

   ☐ Subject lost to follow-up (location unknown)

   ☐ Other, specify ____

   ☐ Subject died – Complete **Serious Adverse Event Form 15 and record SAE#:** ☐☐

   Date of Death: .... ☐☐ Month ☐☐ Day ☐☐ Year

   Cause of Death:

   a. Specific cause of death (based on review of available medical chart data)

   ____

   ____

   b. Primary cause of death was most likely

   ☐ Cardiac related

   ☐ Not cardiac related

   ☐ Unknown

   c. Detailed information about primary cause of death by category

☐ Accident	☐ Cerebrovascular
☐ Suicide	☐ Pulmonary
☐ Infection	☐ Cancer
☐ Cardiovascular	☐ Other

S.I. Signature ______________________________ Date ___________________________

SAE#	*SITE NO.*	*SUBJECT ID*	*ALPHA CODE*	*DATE FORM COMPLETED*
☐☐	☐☐☐	☐☐☐☐	☐☐☐☐	☐☐ *Month* ☐☐ *Day* ☐☐☐☐ *Year*

FORM 15 – SERIOUS ADVERSE EVENT

1. Start Date (date the SAE began) .... ☐☐ *Month* ☐☐ *Day* ☐☐☐☐ *Year*

2. Serious event type: Check all that apply

   ☐ Death

   ☐ Life-threatening

   ☐ Inpatient hospitalization or prolongation of existing hospitalization

   ☐ Non fatal Myocardial Infarction

   ☐ Persistent or significant disability/Incapacity

   ☐ Any other condition that may jeopardize the subject and require medical or surgical treatment to prevent one of the above outcomes

3. Date site investigator became aware of the event .... ☐☐ *Month* ☐☐ *Day* ☐☐☐☐ *Year*

4. Is the SAE attributed to the study?

   ☐ Not Attributed ☐ Possibly Attributed ☐ Definitely Attributed

5. Brief description of the serious adverse event: ____

____

6. Detailed description of the serious adverse event; including treatment of the event (describe subject's condition just prior to, during and after the event. If known, give the duration and outcome of this event. Do not include past medical history):

____

____

____

____

____

SAE#	***SITE NO.***	***SUBJECT ID***	***ALPHA CODE***
☐☐	☐☐☐	☐☐☐☐	☐☐☐☐

7. Pertinent medical history (include pre-existing medical conditions and relevant adverse events previously reported):

____

____

____

____

____

8. Pertinent Test Results/Laboratory Data (include abnormal and normal laboratory results/data and the date(s) of these tests and/or procedures).

____

____

____

____

____

9. Outcome (mark only one)

- ☐ Fatal, Date of Death: .... ☐☐ *Month* ☐☐ *Day* ☐☐☐☐ *Year*
- ☐ Ongoing - Recovering/Resolving
- ☐ Ongoing - Not Recovered/Resolved
- ☐ Recovered/Resolved, Date: .... ☐☐ *Month* ☐☐ *Day* ☐☐☐☐ *Year*
- ☐ Recovered/Resolved with Sequelae (no change expected), requires Stop Date: .... ☐☐ *Month* ☐☐ *Day* ☐☐☐☐ *Year*
- ☐ Unknown

10. Is a Follow-up Serious Adverse Event Form (Form 16) required? .... ☐ No ☐ Yes

S.I. Signature ____ Date ____________________

SAE#	*FOLLOW-UP #*	*SITE NO.*	*SUBJECT ID*	*ALPHA CODE*	*DATE FORM COMPLETED*		
☐☐	☐☐	☐☐☐	☐☐☐☐	☐☐☐☐	☐☐ *Month*	☐☐ *Day*	☐☐☐☐ *Year*

FORM 16 – SERIOUS ADVERSE EVENT FOLLOW-UP

1. Start date of the original Serious Adverse Event (SAE) .... ☐☐ *Month* ☐☐ *Day* ☐☐☐☐ *Year*

2. Has the diagnosis being reported changed from the initial SAE reported on Form 15? .... ☐ No ☐ Yes

   If yes, new diagnosis ____

3. Is there additional new information to report? .... ☐ No ☐ Yes

   If yes, specify ____
   ____
   ____

4. Has the SAE attributability to study surgery changed since the previous report? ☐ No ☐ Yes

   If yes, is the event: ☐ Not Attributed ☐ Possibly Attributed ☐ Definitely Attributed

5. Outcome (mark only one)

   ☐ Fatal, include Date of Death: .... ☐☐ *Month* ☐☐ *Day* ☐☐☐☐ *Year*

   ☐ Ongoing - Recovering/Resolving

   ☐ Ongoing - Not Recovered/Not Resolved

   ☐ Recovered/Resolved, Date: .... ☐☐ *Month* ☐☐ *Day* ☐☐☐☐ *Year*

   ☐ Recovered/Resolved with Sequelae (no change expected), requires Stop Date: .... ☐☐ *Month* ☐☐ *Day* ☐☐☐☐ *Year*

   ☐ Unknown

6. Is another follow-up form expected for this SAE? ☐ No ☐ Yes **If yes, Follow-up #** ☐☐

S.I. Signature ____ Date ____________________

SITE NO.	*HARVESTER ID*	*DATE OF COMPLETION*
☐☐☐	☐☐	☐☐ ☐☐ ☐☐☐☐
		Month *Day* *Year*

FORM 17 – SITE QUALIFICATION BY AVAILABILITY OF VEIN HARVESTER

1. Name of Harvester ____

2. Degree of Training

   ☐ PA-C
   ☐ CRNP
   ☐ MD
   ☐ Other, specify ______________________

3. Years of general experience after completion of formal training in chosen profession

   ☐ <5 years
   ☐ >5 years but <10 years
   ☐ >10 years

4. Name of Supervising Physician……____

5. I have done the following number of **Endoscopic** Vein Harvests (EVH) as of today's date

   ☐ >100 but <500
   ☐ >500 but <1,000
   ☐ >1,000 but <2,000
   ☐ >2,000

If <100 EVH: STOP!
You are not eligible to participate in CSP #588 at this time.
Please return this Form to Jennifer Gabany at the CSP 588 National Office

6. I have expertise with the following EVH systems

   ☐ VasoView® (MAQUET)
   ☐ VirtuoSaph® (TERUMO)
   ☐ both

SITE NO. ☐☐☐ *HARVESTER ID* ☐☐

7. Number of Open (without endoscope; including bridging technique) Vein Harvests (OVH) as of today's date
   - ☐ <50
   - ☐ >50 but <100
   - ☐ >100 but <500
   - ☐ >500 but <1000
   - ☐ >1000 but < 2000
   - ☐ >2000
8. My conversion Rate from EVH to OVH in Last 100 EVH Cases
   - ☐ 5%
   - ☐ >3% but <5%
   - ☐ >1% but <3%
   - ☐ <1%
9. The above Harvester is experienced in: ☐ **both EVH and OVH** ☐ **EVH only**

Signature of Site Surgeon Investigator

____

Printed Name of Site Surgeon Investigator

____

____

Date

EVENT NO.	SITE NO.	SUBJECT ID	ALPHA CODE	DATE OF NONCOMPLIANCE
				Month Day Year

FORM 18 – PROTOCOL NONCOMPLIANCE

1. Noncompliance Code ....

2. Details (cause of, result, resolution, etc.)
____
____
____
____
____
____

3. Perry Point CSPCC notified on .... Month Day Year

4. IRB notified on .... Month Day Year
   (code "A" if not notified, note reason in chart)

5. Chairman's Office notified on .... Month Day Year

9. Did the noncompliance result in a serious adverse event .... No ☐ Yes ☐
   (If yes, complete Form 15 – Serious Adverse Event Form)

S.I. Signature ____ Date ____________________

Protocol Noncompliance Codes

01	SAE not reported
02	SAE reported late
03	Subject not monitored for SAE
04	Did not follow instructions from IRB or other review bodies/committees
05	Confidentiality or privacy breach
06	Loss of source documents/samples/source media
07	Ineligible subject enrolled
08	Subject in more than one simultaneous interventional trial
09	Informed Consent/HIPAA documentation completed incorrectly
10	Informed Consent/HIPAA documentation is incomplete
11	Informed Consent/HIPAA not obtained prior to study procedures
12	Used incorrect informed consent/HIPAA version
13	Required study procedure not performed per protocol
14	Study activities performed by inappropriate personnel
15	Study intervention not administered per protocol
16	Subject noncompliance
17	Other (specify under Details of noncompliance

MI #	SITE NO.	SUBJECT ID	ALPHA CODE	DATE FORM COMPLETED
☐	☐☐☐	☐☐☐☐	☐☐☐☐	☐☐ Month ☐☐ Day ☐☐☐☐ Year

FORM 19 – CONFIRMATION OF MYOCARDIAL INFARCTION BY LOCAL SITE

1. Date of study surgery (index CABG) .... ☐☐ Month ☐☐ Day ☐☐☐☐ Year
2. Date of myocardial infarction (MI) .... ☐☐ Month ☐☐ Day ☐☐☐☐ Year
3. Supporting documentation (universal definition of MI):

ECG Changes *All that apply*

- ☐ Development of new ST-T changes
- ☐ Development of new LBBB
- ☐ Development of new pathologic Q-Waves

If any ECG changes marked above, **you must complete A, B, OR C below**

A. Peak Cardiac Troponin I

1. Peak Cardiac Troponin I (ng/mL): ☐☐.☐☐
2. Reference Range (ng/mL): ☐.☐☐ to ☐.☐☐

B. Peak Cardiac Troponin T

1. Peak Cardiac Troponin T (ng/mL): ☐☐.☐☐
2. Reference Range (ng/mL) ☐.☐☐ to ☐.☐☐

C. Peak CPK/MB Panel

1. Peak Total CPK (U/L): ☐☐☐☐
2. Total CPK Reference Range (U/L): ☐☐ to ☐☐☐
3. Peak CK-MB (ng/mL): ☐☐.☐
4. CK-MB Reference Range (ng/mL): ☐ to ☐.☐
5. Peak CK-MB Index (%): ☐☐.☐
6. CK-MB Index Reference Range (%): ☐.☐ to ☐.☐

MI #	***SITE NO.***	***SUBJECT ID***	***ALPHA CODE***
☐	☐☐☐	☐☐☐☐	☐☐☐☐

4. Other Supporting Documentation (check all that apply)

   ☐ Imaging evidence of loss of viable myocardium or new regional wall motion abnormality

   ☐ Symptoms of ischemia

   ☐ Angiographic documenting new graft or new coronary occlusion

S.I. Signature ____ Date ___________________

MI #	*SITE NO.*	*SUBJECT ID*	*ALPHA CODE*	*DATE FORM COMPLETED*		
☐	☐☐☐	☐☐☐☐	☐☐☐☐	☐☐	☐☐	☐☐☐☐
				Month	*Day*	*Year*

FORM 20 – CONFIRMATION OF MYOCARDIAL INFARCTION BY CLINICAL EVENTS COMMITTEE

1. Clinical Events Committee Confirms Diagnosis of Myocardial Infarction: No ☐ Yes ☐

   If yes, proceed to question 2.

   If no, STOP, sign form and submit to the Perry Point Coordinating Center.

2. Classification of myocardial infarction: (select one)

   ☐ Type 1 Spontaneous myocardial infarction

   ☐ Type 2 Myocardial infarction secondary to an ischemic imbalance

   ☐ Type 3 Myocardial infarction resulting in death when biomarker values are unavailable

   ☐ Type 4a Myocardial infarction related to percutaneous coronary intervention (PCI)

   ☐ Type 4b Myocardial infarction related to stent thrombosis

   ☐ Type 5 Myocardial infarction related to coronary artery bypass grafting (CABG)

3. Committee comments (if applicable): ____

   ____

   ____

   ____

   ____

Form Completed By: ____ Date ______________

____________________________________/_________________________ ___________________

Signature of Chair, Clinical Events Committee / PRINTED NAME Date

SITE NO.	*SUBJECT ID*	*ALPHA CODE*	*DATE FORM COMPLETED*
☐☐☐	☐☐☐☐	☐☐☐☐	☐☐ ☐☐ ☐☐☐☐
			Month Day Year

FORM 21 – CAUSE OF DEATH BY CLINICAL EVENTS COMMITTEE

1. Date of Death .... ☐☐ ☐☐ ☐☐☐☐
Month Day Year

Cause of Death

a. Specific cause of death (based on review of available medical chart data)

____

____

b. Primary cause of death was most likely

☐ Cardiac related

☐ Not cardiac related

☐ Unknown

c. Detailed information about primary cause of death by category

☐ Accident	☐ Cerebrovascular
☐ Suicide	☐ Pulmonary
☐ Infection	☐ Cancer
☐ Cardiovascular	☐ Other

Completed by ____ Date ____________

VA COOPERATIVE STUDY #588

RANDOMIZED ENDO-VEIN GRAFT PROSPECTIVE – REGROUP TRIAL

Site Selection Process: Vein Harvester Qualification Policy

1. VA sites with active Cardiac Surgery Programs are contacted with introductory letter including executive summary and relevant manuscripts via email.

2. Interested sites provide high-level overview of the sites Cardiac Surgery Service eligibility criteria:
   - established EVH program >2years,
   - sufficient isolated CABG volume to enroll 2-3 subjects/month x3 years,
   - willingness to randomize to EVH vs. OVH,
   - at least one harvester experience includes > 100 EVH with conversion rate $\leq$5%,
   - ability to competently provide care for OVH procedure.

3. All potential sites complete CSP #588 Form 15 for each individual harvester who is expected to perform the vein harvesting procedure as part of normal daily job duties during the course of study participation at the site. The forms are signed by the site Principle Investigator (site surgeon investigator) as verification.

4. Each completed form is submitted to and reviewed by the Study Chair's office.

5. A Subcommittee of the Executive Committee will review each site case by case to determine eligibility status and designate each site as one of the following:

   A. Site with at least one harvester experienced in BOTH EVH and OVH confirmed by the site surgeon investigator **=>** site confirmed as qualified for study participation and harvester assigned a code by CSPCC.

   B. Site with at least one harvester experienced in EVH but NOT OVH confirmed by the site surgeon investigator **=>** site confirmed as qualified for study participation only IF a designated harvester for OVH is identified, assigned a code by CSPCC, and MUST be immediately available, in addition to the EVH harvester, at time of randomization and harvesting (i.e. the site surgeon investigator).

   I. Site with NO harvester experienced in EVH and/or incapable of providing OVH procedure**=>** site is INELIGIBLE for study participation at this time regardless of interest level or CABG volume.

Subcommittee Members: Marco Zenati MD, MSc, FETCS; Jerene Bitondo, PA-C; Jennifer Gabany, CRNP

VA Maryland Healthcare System

Cooperative Study CSP #588

Randomized Endo-Vein Graft Prospective (REGROUP) Trial

Statistical Analysis Plan

Version Number 3
August 30, 2018

Privileged & Confidential

This document is a confidential communication of the Veteran's Administration. Acceptance of this document constitutes agreement by the recipient that no unpublished information contained herein will be published or disclosed without the Veteran's Administration prior written approval, except that this document may be disclosed to appropriate Institutional Review Boards under the condition that they are requested to keep it confidential.

STATISTICAL ANALYSIS APPROVAL

Version 3.0

Responsibility	Name	Signature	Date
**PPCSPCC Study Biostatistician**	Eileen M. Stock, PhD		
**Study Chairman**	Marco A. Zenati, MD, MSc, FETCS		
**PPCSPCC Director**	Kousick Biswas, PhD		

Table of Contents

1 INTRODUCTION 1
1.1 Preface 1
1.2 Goal of the analyses 1
2 STUDY OBJECTIVES AND ENDPOINTS 1
2.1 Study Objectives and Hypotheses 1
2.2 Endpoints 2
2.3 Derived variables 4
2.3.1 Seattle Angina Questionnaire 4
2.3.2 VR-12 4
2.3.3 Syntax Score 4
2.3.4 VASQIP Score 4
2.3.5 STS Score 4
2.3.6 Leg Incision Pain Impact Questionnaire 5
2.3.7 Leg Incision Assessment 5
2.3.8 Treatment Groups 5
3 STUDY METHODS 5
3.1 General Study Design and Plan 5
3.2 Screening and Baseline Assessments 6
3.3 Inclusion-Exclusion Criteria 6
3.3.1 Participant Population 6
3.3.2 Inclusion Criteria 6
3.3.3 Exclusion Criteria 7
3.4 Randomization and Blinding 7
3.5 Forms for Screening, Baseline, and Follow-up Visits 7
TABLE 1: Schedule of Assessments: Screening, Baseline, and Follow-up Data Forms......8
3.6 Sample Size 9
4 STUDY AND DATA MANAGEMENT 9
4.1 Study Management at the CSPCC 9
4.2 Randomization and Data Management 10
5 GENERAL CONSIDERATIONS 12
5.1 Timing of Analyses 12
5.2 Analysis Populations 12
5.3 Missing Data and Imputations 13
5.4 General Considerations 13
5.5 Interim Monitoring 14
5.5.1 Data Monitoring Committee 14
5.5.1.1 Stopping Rules 15

5.5.2 Executive Committee 16
6 SUMMARY OF STUDY DATA 16
6.1 Subject Disposition 16
6.2 Demographics 17
6.3 Baseline Variables 17
6.4 Treatment Exposure 18
6.5 Study Endpoint 18
6.6 MACE by Harvester Experience 18
6.7 Serious Adverse Events 19
6.8 Protocol Non-compliance 19
7 STATISTICAL ANALYSES 19
7.1 Primary Outcome 19
7.2 Secondary Outcomes 20
7.3 Tertiary Outcomes 21
8 SERIOUS ADVERSE EVENTS 22
9 STATISTICAL PROGRAM VALIDATION PLAN 22
APPENDIX 1: TABLES AND FIGURES 24
TABLE 2: Number of Participants Screened, Eligible, Consented, Randomized and Expected Randomized, Total and by Medical Center 24
TABLE 3: Participants Screened and Randomized by Month, Total and by Medical Center 25
FIGURE 1: Randomization Activities Based on Number of Active Centers per Month.....28
TABLE 4: Reasons for Ineligibility, Total and by Medical Center 29
TABLE 5: Reasons for Non-Enrollment, Total and by Medical Center 30
TABLE 6: Reasons for Non-Randomization, Total and by Medical Center 31
TABLE 7: Status of Randomized Participants, Total and by Medical Center 32
TABLE 8: Summary of Demographics, Total and by Medical Center 33
TABLE 9: Seattle Angina Questionnaire: [*Component scale here*] at Baseline, by Medical Center and Treatment 36
TABLE 10: Veterans Rand 12 Item Health Survey: [*Component scale here*] at Baseline, by Medical Center and Treatment 37
TABLE 11: Conversion to Open Harvest Procedure, by Medical Center and Harvester, in Patients Randomized to Endoscopic Harvest Procedure. 38
TABLE 12: Reasons for Conversion to Open Harvest Procedure, by Medical Center 40
TABLE 13: Conversion to Open Harvest Procedure, by Medical Center and Harvester, Excluding Conversions Completed Because Unanticipated Graft Required Additional Vein, in Patients Randomized to Endoscopic Harvest Procedure..........41
TABLE 14: Mean [SYNTAX Score, VASQIP Patient Risk Calculation, STS Risk of Mortality], Total and by Medical Center and Treatment 43

TABLE 15: Mean [SYNTAX Score, VASQIP Patient Risk Calculation, STS Risk of Mortality], by MACE and Treatment....44
TABLE 16: Mean [SYNTAX Score, VASQIP Patient Risk Calculation, STS Risk of Mortality], by Age Group and Treatment....45
TABLE 17: Mean [SYNTAX Score, VASQIP Patient Risk Calculation, STS Risk of Mortality], by Diabetes Status and Treatment....45
TABLE 18: Bilateral Mammary Artery Grafting, Total and by Medical Center and Treatment....46
TABLE 21: Major Adverse Cardiac Events, Overall and by Treatment....50
TABLE 22: Mean Length of Follow-up in Days, by Medical Center and Treatment....51
TABLE 23: Cumulative Serious Adverse Event Incidence using MedDRA coding after randomization, by Body System and Preferred Term....52
TABLE 24: Cumulative Serious Adverse Event Incidence using MedDRA coding after randomization, by Body System and Preferred Term....52
FIGURE 2: Kaplan-Meier Survival Curves....53
TABLE 25: MACE, Active Follow-up: Results of Multivariable Cox Proportional Hazards Regression Model....54
TABLE 26: Major Adverse Cardiac Events at 1-year and 3-year, by Treatment....54
TABLE 27: Leg Incision Healing by Treatment....55
TABLE 28: Leg Incision Healing by Treatment: Results of Multivariable Logistic Regression Model....55
TABLE 29: Seattle Angina Questionnaire: Change from Baseline by Treatment....56
TABLE 30: Seattle Angina Questionnaire: Results of ANCOVA Model....56
TABLE 31: Impact and Severity of Incisional Leg Pain at by Treatment....57
TABLE 32: Veterans Rand 12 Item Health Survey: [*Component scale here*] by Medical Center and Treatment....58

1 INTRODUCTION

1.1 Preface

Coronary artery bypass grafting (CABG) is the most common major surgical procedure in the United States with over 300,000 cases performed each year. To restore blood flow to the heart, vascular conduits from another part of the body are procured to create a bypass around critically blocked coronary arteries. The left internal thoracic artery is the conduit of choice for CABG due to its superior long-term patency. However, almost all patients referred for CABG require additional grafts to provide complete revascularization. This necessitates the harvest of other vessels, most commonly the saphenous vein which is used almost ubiquitously in contemporary CABG with an average of two vein grafts per CABG procedure. In the last 10 years, endoscopic vein harvesting (EVH) has been recommended as the preferred method over the traditional open harvesting technique (OVH) for saphenous vein graft (SVG) harvesting because it provides a minimally invasive approach. However, more recent investigations indicate potential for reduced long-term bypass graft patency and worse clinical outcomes with EVH. The long-term impact of EVH on clinical outcomes has never been investigated on a large scale using a definitive, adequately powered, prospective Randomized Clinical Trial (RCT) with long-term follow-up. Results of the proposed study will fill a significant gap in existing knowledge regarding long-term outcomes for EVH in CABG, improving the quality of the care we provide to Veterans and more broadly to all patients undergoing coronary revascularization. Findings from this study have the potential to significantly impact the VA and national cardiac surgery coronary revascularization guidelines.

1.2 Goal of the analyses

The goal is to investigate the impact of SVG harvesting technique (OVH vs. EVH) on MACE (major adverse cardiac events), a composite end point of all-cause mortality, nonfatal myocardial infarction and repeat revascularization, leg wound complication, patient satisfaction, and quality of life in addition to determining the role of vein harvester's experience on clinical outcomes.

2 STUDY OBJECTIVES AND ENDPOINTS

2.1 Study Objectives and Hypotheses

Primary Objectives:

Objective 1: To investigate the impact of SVG harvesting techniques – OVH vs. EVH on MACE, a composite end point of all-cause mortality, nonfatal myocardial infarction and repeat revascularization, over the active follow-up period of the study postoperatively.

Hypothesis 1: A significantly smaller proportion of CABG subjects with SVGs harvested by open technique will experience MACE post-surgery compared to CABG subjects with SVGs harvested by endoscopic technique during the active follow-up period.

Secondary Objectives:

Objective 2: To investigate the impact of SVG harvesting techniques – OVH vs. EVH on MACE, a composite endpoint of all-cause mortality, nonfatal myocardial infarction and repeat revascularization, at one and three-year postoperatively.

Hypothesis 2: One-year composite MACE rate will be 6 percentage points lower in the open harvesting group and three-year composite MACE rates will be at least 8-10 percentage points lower in the open vein harvesting group compared to the endoscopic vein harvesting group.

Objective 3: To investigate the impact of SVG harvesting techniques – OVH vs. EVH on MACE, a composite endpoint of all-cause mortality, nonfatal myocardial infarction and repeat revascularization, over the entire follow-up period (active and passive) of the study postoperatively.

Hypothesis 3: A significant smaller proportion of CABG subjects with SVGs harvested by open technique will experience MACE post-surgery compared to CABG subjects with SVGs harvested by endoscopic technique during the entire follow-up period.

Tertiary Objectives:

Objective 4: Investigate the impact of the two harvesting techniques - open vs. endoscopic - on clinical indicators of leg wound complications and subject satisfaction at six weeks post-surgery.

Hypothesis 4: Leg wound complications will be lower and satisfaction will be higher in the EVH group compared to OVH group.

Objective 5: Compare subject quality of life scores according to the SVG harvest technique at six weeks post-surgery.

Hypothesis 5: Subjects' quality of life scores will be higher in the EVH group compared to the OVH group.

Objective 6: Determine the role of vein harvester experience on clinical outcomes.

Exploratory Aim with no hypothesis specified.

2.2 Endpoints

2.2.1 Primary Outcome Measures

The primary outcome measure is MACE following randomization and the index CABG.

For hypothesis 1: The outcome measure, MACE (major adverse cardiac events) is a composite endpoint that includes death (all-cause), myocardial infarction (MI), and revascularization for myocardial ischemia. Each randomized participant (either in EVH or OVH) will be followed after the index CABG to capture the time-to-MACE event where an 'EVENT' will be defined as either death (all-cause) or an MI or a revascularization procedure during the follow-up period. Total follow-up period will be 6.5 years of which the first 4.5 years will be active follow-up (using in-clinic visit or by telephone) period and will be carried out by the site personnel. The remaining 2 years will be passive follow-up which will be carried out centrally by the chair's office staff using VA clinical and administrative databases (CPRS, VASQIP, etc.). A minimum of 1 year of active follow-up will be used for participants who will be randomized at the tail end of the 3-year projected enrollment period of the study. The participants who will either be lost to follow-up or will not experience an 'EVENT' before the end of the follow-up period will be considered as right-censored. The primary analysis of such time-to-Mace event data will include the events only from the active follow-up period (which is approximately 4.5 years).

2.2.2 Secondary and Tertiary Outcome Measures

Secondary outcomes include MACE at one and three-year and over the entire follow-up period (active and passive) of the study postoperatively. Tertiary outcomes include leg wound complication and quality of life.

For hypothesis 2: MACE at one and three-year postoperatively.

For hypothesis 3: MACE over the entire follow-up period (active and passive) of the study postoperatively.

For hypothesis 4: Assessment of leg wound complications will be assessed at the time of discharge and at approximately six-week post-surgery during a clinic visit (this visit will take place between approximately 4 to 8 weeks post-surgery). Post-operative leg wound complication status (yes/no) will be determined based on these assessments.

For hypothesis 5: The Quality of Life (QoL) outcome measure will be the summary measures from the VR-12 instrument of health related quality of life as measured by the Physical Component Summary (PCS) and Mental Component Summary (MCS) and the Seattle Angina Questionnaire. QoL self-assessments will be completed at baseline, six weeks, and one year (by mail or telephone). Additionally, participants will be categorized as "improved", "no change", or "worsened" based on their baseline scores.

For hypothesis 6: Clinical measures include MACE.

2.3 Derived variables

2.3.1 Seattle Angina Questionnaire

The Seattle Angina Questionnaire (SAQ) is the leading health-related QoL measure for patients with coronary artery disease. The self-assessment questionnaire consists of a list of activities people often do during the week (e.g., dressing yourself, walking indoors on level ground, showering) and asks participants the amount of limitation (ranging from not at all limited to extremely limited) they have doing these activities due to chest pain, chest tightness, or angina over the previous 4 weeks. Additional questions ask about frequency and treatment of chest pain, chest tightness, or angina.

The answers provided on the SAQ are used to calculate scores in five scales: 1) Angina Stability, a measure of whether a patient's symptoms are changing over time; 2) Angina Frequency, a measure of how often a patient is having symptoms now; 3) Physical Limitation, a measure of how much a patient's condition is hampering his ability to do what he wants to do; 4) Treatment Satisfaction, a measure of well a patient understands her care and what she thinks of it; and 5) Quality of Life, a measure of the overall impact of a patient's condition on a patient's interpersonal relationships and state of mind. Participants will complete the SAQ at baseline, 6 weeks, and 1 year post-surgery. Higher scores are better. For follow-up assessments, participants will be categorized as "improved", "no change", or "worsened" based on their baseline scores.

2.3.2 VR-12

The Veterans RAND 12 Item Health Survey (VR-12) is a self-administered questionnaire with both physical and psychological domains using no more than 10 questions to create physical (PCS) and mental (MCS) component summary scores. The scoring of the PCS and MCS for the VR-12 is based on weights derived from the VR-36 instrument administered to 1.4 million Veteran enrollees with 877,775 respondents in the 1999 Large Health Survey of Veteran Enrollees (Veterans Health Study) (Iqbal et al. 2009). Higher PCS and MCS scores reflect greater quality of life. Imputation methods will be used to calculate scores for Veterans who do not complete all 12 questions included in this measure. Participants will complete the VR-12 at baseline, 6 weeks, and 1 year post-surgery. For follow-up assessments, change from baseline will be examined. Higher PCS and MCS scores reflect greater quality of life.

2.3.3 Syntax Score

The SYNTAX Score is a unique tool to score the complexity of coronary artery disease (available at www.syntaxscore.com). Higher scores are associated with greater risk.

2.3.4 VASQIP Score

The VA Surgical Quality Improvement Program (VASQIP) score will be used to assess 30-day surgical risk of mortality. Higher scores are associated with greater risk.

2.3.5 STS Score

The Society of Thoracic Surgeons (STS) score will be used to assess risk of operative mortality and morbidity of adult cardiac surgery on the basis of patient demographic and clinical characteristics. Higher scores are associated with greater risk.

2.3.6 Leg Incision Pain Impact Questionnaire

Severe leg pain, due to incisions made during vein grafting, data will be collected at discharge and at 4-6 weeks post-CABG. The Pain Impact Questionnaire (PIQ) is a 6-item measure of pain severity and its impact on health-related QoL (Becker et al. 2005). Impact scores range from 40 to 78 with higher scores reflecting greater pain impact. Scores can be groups into: 1) Little or No Impact (impact score ≤ 50); 2) Some Impact (50 < impact score ≤ 57); 3) Substantial Impact (57 < impact score ≤ 63); and 4) Severe Impact (impact score > 63). A pain severity rating is also calculated from the first PIQ item and ranges from 1 to 6, with severe pain defined as a score of 3 (mild) or above.

2.3.7 Leg Incision Assessment

The leg incision assessment calculates asepsis score based on information gathered at discharge and 4-6 weeks follow-up. At discharge ratings are given to the severity of serious exudates, erythema, purulent exudates, and separation of tissues. At follow-up, antibiotic use, drainage under local anesthetic, debridement under general anesthetic, bacterial isolation, hospital stay prolonged >14 days, development of pus as an outpatient, and visiting nurse visit to dress wound are assessed (Wilson et al. 1986). Then, responses are summed to create a total asepsis score.

2.3.8 Treatment Groups

Veteran patients will be randomized to receive one of two possible vein harvesting procedures:

- Open vein harvesting (OVH) – the traditional method of saphenectomy for CABG performed under direct vision using a single long incision or, more commonly, multiple smaller incisions (referred to as "bridging" technique) along the course of the vein. This approach minimizes manipulation and direct trauma to the conduit but is associated with potential for discomfort and leg wound healing complications.

- Endoscopic vein harvesting (EVH) – a minimally invasive procedure that was developed to eliminate the need for long incisions associated with OVH. EVH reduces the risk of wound infections and other leg wound complications but may be more traumatic to the conduit than OVH.

3 STUDY METHODS

3.1 General Study Design and Plan

This study is a randomized, intent-to-treat, two-arm, parallel design, multicenter study to compare clinical outcomes of major adverse cardiac events (MACE) of CABG patients treated with SVG harvested with EVH and OVH during the trial period. Cardiac Surgery Programs at Veterans Affairs Medical Centers (VAMCs) with expertise in performing both EVH and OVH are eligible to participate in the study (EVH Program established for more than two years and at least 100 successful EVH cases performed by each mid-level provider or other designated individual involved with the study) (Desai et al. 2011). Participants requiring elective or urgent CABG using cardiopulmonary bypass with use of at least one SVG will be screened using established inclusion/exclusion criteria. Participants will be randomized to one of the two arms (EVH or OVH) after an experienced vein harvester is identified and assigned to the case. Assessments will be collected at multiple time points including: baseline, intraoperatively, postoperatively, and at discharge or 30 days after surgery if still hospitalized. Assessment of leg wound complications will be completed at the time of discharge and at approximately six weeks post-surgery. Quality of life self-assessments will be completed at baseline, six weeks, and one year (by mail or telephone). Telephone follow-ups will occur at three-month intervals post-surgery until the participating centers are decommissioned at the end of the trial period (which would be approximately 4.5 years after the center initiations). For long-term MACE outcomes, passive follow-up for MACE using VA clinical and administrative databases (CPRS, VASQIP, etc.) will be performed centrally by the Study Chair's office for another 2 years. This study will enter approximately 1,150 participants in 16 participating VA medical centers.

3.2 Screening and Baseline Assessments

The research study coordinator will be primarily responsible for identifying each non-emergent patient scheduled for a CABG-only procedure with planned SVG harvesting. A diagnostic catheterization must be performed within six months prior to the scheduled operation to be used as part of the baseline assessments. The participating surgeon(s) must agree that the patient is eligible for either study arm before randomization based on the inclusion/exclusion criteria defined by the protocol. This includes a review of the medical history for any lower extremity issues that would prevent the harvest of an effective SVG such as varicose veins, etc. Following participant informed consent, the baseline risk assessment, clinical data and participant self-reported symptom status and health-related quality of life data will be obtained by the study team.

3.3 Inclusion-Exclusion Criteria

3.3.1 Participant Population

All participants who are candidates for CABG and who will undergo surgery at a VAMC with demonstrated expertise for both OVH and EVH and qualify to participate in CSP-sponsored research will be invited to participate in the REGROUP study.

3.3.2 Inclusion Criteria

1) Age 18 years or older

2) Elective or Urgent CABG-only
3) Median sternotomy approach
4) At least one coronary bypass planned using saphenous vein graft for conduit
5) Experienced EVH/OVH harvester and participating surgeon available for procedure

3.3.3 Exclusion Criteria

1) Combined valve procedure planned
2) Moderate or severe valve disease (see definition of moderate/severe valve)
3) Hemodynamically unstable or in cardiogenic shock
4) Enrolled in another therapeutic or interventional study
5) Off-pump CABG procedure planned
6) Limited life expectancy < 1 year
7) History of lower extremities venous stripping or ligation
8) Inability to provide informed consent

3.4 Randomization and Blinding

A block randomization scheme will be used to randomize participants in two treatment groups. The block randomization technique will ensure equal distribution of participants, within each harvester, within each medical center, in both arms of the trial. A random sequence of block sizes will be used to reduce the chances of guessing future allocations. The randomization schema will be generated using SAS (SAS, Cary, NC). All participating vein harvesters must meet the minimum EVH/OVH volume criteria [at least 100 EVH cases with low conversion rates (<5%) as part of an EVH program established for more than two years] to be eligible to enroll patients in this study. Unless an urgent medical condition exists, the participant's surgery will be scheduled to occur at the earliest possible date based on expert harvester availability and other center circumstances. The randomization procedure will occur after the participating expert harvester is assigned to the participant. Participants will be randomized within the assigned participating harvesters to one of the two study harvesting technique arms (EVH or OVH). Recognizing that the participant's assignment to a participating vein harvester will have already occurred, the study randomization to either EVH or OVH will be done by a telephone call to the Perry Point Cooperative Studies Program Coordinating Center (CSPCC).

3.5 Forms for Screening, Baseline, and Follow-up Visits

Baseline assessments will be collected prior to surgery and randomization. Intraoperative assessments will be collected during the CABG procedure, 24 and 48 hours postoperatively. Assessments will also be collected at the time of hospital discharge or 30 days post-surgery, whichever occurs first. Participants will return for a six-week clinic visit to assess the condition of their leg incision and healing status. Participants will receive a phone call every three months for follow-up for events until participant termination. Participants will complete QoL surveys again at one year either by telephone or mail. The following forms in Table 1 will be used during the screening, baseline, and follow-up visits of the study.

TABLE 1: Schedule of Assessments: Screening, Baseline, and Follow-up Data Forms

FORM	SCREEN	BASELINE (pre op) Visit 00	INTRA OP Visit 00	POST OP Visit 00	DC- 30 DAY Visit 01	6 WK Visit 02	3 MO Visit 03	6 MO Visit 06	9 MO Visit 09	12 Mo Visit 12	… Every 3 Mo	45 MO Visit 45	49 MO Visit 49	AS NEEDED
00 – Screening Log	X													
01 – Screening and Randomization	X													
02 – Baseline Information		X												
03 – Seattle Angina Questionnaire		X				X				X	Mo 36 only Δ			
04 – VR-12		X				X				X	Mo 36 only Δ			
05 – Intraoperative Data Collection			X											
06 – Post Operative Assessments				X										
07 – Discharge Assessments					X									
08 –Leg Incision Pain Questionnaire					X									
09 – Leg Incision Pain 6 week						X								
10 – Leg Incision Assessment					X*	X*								
11 – Mace Event (6 week)						X								
12 – Phone Call Follow-up							X	X	X	X	X	X	X	
13 – MACE Event Form													X	
14 - Termination														X
15 - SAE														X
16 – SAE Follow-up														X
17 – Harvester Experience														X
18 – Protocol Noncompliance														X
19 – Confirmation of MI by Local [illegible]														X
20 – Confirmation of MI by Clinical Events Committee														X
21 – Cause of Death by Clinical Events Committee														X
22 – Tobacco Use and CPB											Mo 36 only			
86 - Consent	X													

* Form 10 is collected at two time points (discharge & 6 weeks); do not fax form until the 6-week assessment has been completed.

Δ Per request from HERC, the VR-12 and SAQ at Month 36 have been added.

3.6 Sample Size

In the ROOBY study (CSP # 517) (Shroyer et al. 2009), 1-year MACE rates were 9.9% in the OVH group and 15.3% in the EVH group (p=0.0025). The executive committee for the REGROUP study assumed that during the REGROUP study, 15.5% of the participants in the EVH group will experience MACE in the first year post surgery. The committee also expects a 6 percentage point improvement in the 1-year MACE rate in the OVH group.

To detect the expected 6 percentage point difference in 1-year MACE rates between EVH (15.5%) and OVH (9.5%), a sample size of 545 in each group will be required at 85% power, 5% type-I error rate and with a two-sided test. Since, it would be possible to capture the majority of the MACE from the VA databases even if participants drop out before the one-year clinic visit, a relatively small inflation factor of 5% is used to inflate the sample size to account for the drop-outs. Thus, a total of 1,150 participants need to be randomized in the study to achieve the said power.

4 STUDY AND DATA MANAGEMENT

4.1 Study Management at the CSPCC

A Cooperative Studies Program Coordinating Center (CSPCC) study team has been assigned to this study for providing data management, statistical, and administrative supports to the study executive committee for a smooth conduct and timely completion of the study. The study team is comprised of:

Biostatistician and Team Lead	Eileen Stock, Ph.D.
Project Manager	Annette Wiseman
Statistical Programmer	Ellen DeMatt, M.A.
Database Programmers	Christine Dalzell
Computer Assistant	Daniel Briones
Computer Assistant	Mike Beam

Other core CSPCC staff, for example, Quality Assurance, Travel Clerk, Printer, Secretary, etc., will provide help based on the need of the study.

The Biostatistician is the study team leader and has the overall responsibility for the conduct of the study at the CSPCC. S/he is the CSPCC's spokesperson to the Study Group; s/he represents the CSPCC on the study's Executive Committee and along with the Study Chairpersons, she is responsible for representing the study at the Data Monitoring Committee meetings. The Biostatistician is also responsible for providing the Study Group with statistical and clinical trial advice, for working with other CSPCC team members in the preparation of routine interim reports, and for conducting the final analyses at the end of the study.

The Project Manager is responsible for the administrative coordination of the study by the CSPCC. S/he serves as the Biostatistician's Administrative Assistant and works with the CSPCC study team to ensure that all reports, study materials, and meeting arrangement notices are sent to the proper individuals in a timely fashion. S/he will work closely with the National Study Coordinator in the Chairman's office to ensure that the study runs smoothly and will be in contact with both the National Study Coordinator and the Local Research Coordinators at the participating centers at least monthly to discuss any problems that they may be having, including those with the CSPCC. S/he will also work with the local VA R&D Offices at the participating centers to obtain R&D and IRB approvals at the beginning of the study and annually as well as the preparation of study budgets yearly during the ongoing phases of the study.

The Statistical Programmer is responsible for the preparation of the tables and analyses for all of the routine study reports. These include Study Group, Executive Committee, Data Monitoring Committee, and the mid-study report to CSSEC. S/he also prepares the tables and reports for the final analyses. S/he works closely with the Biostatistician on these analyses.

The Database Management System (DBMS) Programmer is the lead of the data management support group and works closely with the assigned computer assistant(s) to address the data management need for the assigned study. S/he is responsible for establishing, updating and maintaining the study's database. In addition, s/he will write edit program based on an agreed upon edit plan that will thoroughly check the data for errors and missing information. S/he is also responsible for programming and maintaining the randomization system for the study.

The Computer Assistant(s) are responsible for setting up the data definition table for the study, for building the Study Definition Editor and also for laying out the electronic case report forms in the form design software. They are also responsible for training the study staff at each site on how to properly manage the data collection process and how to appropriately respond to data edits. The computer assistant(s) are also responsible for working with the sites to resolve the data queries generated based on the incomplete and/or inaccurate data submitted to the study database.

4.2 Randomization and Data Management

Randomization and data management will be performed by the Perry Point CSPCC. An Interactive Touchtone Telephone Randomization System (ITTRS) will be used to set up the randomization system. Clinical DataFax System, a data management software will be used for data management. The CSPCC will have overall responsibility for the data at the end of the study.

After a patient at any of the participating centers is consented, successfully screened and has provided baseline information, s/he will be assigned to a harvester. Once a harvester is assigned and available for the harvesting of the required vein, the patient will be randomized. The research coordinator will place a

call to the ITTRS (a dedicated 1-800 phone number will be provided) to randomize the patient in one of the two harvesting techniques – endoscopic and open vein harvesting techniques. Once the required information is entered in the system, the system will return the assignment for the patient. This study will use a “permuted block” randomization scheme where random block sizes of two and four will be used. The research coordinator will need the following information in order to complete a successful randomization call:

a. Study number and study password (will be provided by CSPCC)
b. 3-digit site number and password (will be provided by CSPCC)
c. The participant’s ID Number & ALPHA Code
d. The participant’s signed Informed Consent Form
e. Form 01, Screening and Randomization
f. Laminated Randomization Cheat Sheet

The system for data capturing will be designed by visits where a group of required case report forms (CRFs) will be assigned to each “visit” according to the “Schedule of Assessments” that was provided in Table 1.

When a participating site has a potential study participant that meets all of the eligibility requirements, the site investigator (SI) or site coordinator (SC) (or other local study team member designee) will assign a unique participant ID and Alpha code to the participant. This unique Participant ID number and alpha code will be entered on all study related-forms for the duration of the study.

Paper CRFs will be mailed to the sites. The research personnel will be completing the CRFs during CABG, the 6-week clinic visit, and the 3-month phone calls. The completed CRFs will then be scanned in pdf and sent to Perry Point CSPCC via secure electronic server or posted on an ftp server. The DataFax system will have built-in edit checks on data fields to minimize data errors, such as missing, inconsistent, or extremely unusual data. At CSPCC, the data management section staff will validate the CRFs once received by the DataFax system and will generate QC reports listing data discrepancies and other irregularities at regular intervals. These QC reports will be sent to the respective sites for clarifications and the site personnel will then submit “Refaxes” with clarification which will be validated and committed to the study master database (A “Refax” is a page of a CRF with corrections which is sent back to the CSPCC by agreed upon mode of CRF transmission). The final responsibility for the completeness and accuracy of all study data collected at a participating site resides with the SI who will review all data before submission. The study database will be continuously updated with new data and changes to previously submitted data. To notify the participating sites about missing or late forms, reports with pertinent information will be generated at a regular interval and will be posted on a site-accessible sub-SharePoint site.

In addition, a summary report of all data submitted and problems identified will be generated for each participating site. This report will provide each site with a summary of their progress. The National Study Coordinator in the Chairman's Office will also be reviewing each site's progress to ensure that there are no unforeseen problems with the forms or with a particular participant.

Another mechanism used to monitor the data and the progress of the study will be the preparation of periodic reports for various groups who are responsible for overseeing the conduct of the study. These groups include the Study Group, the Executive Committee, the Data Monitoring Committee, and the CSPCC Human Rights Committee, if applicable.

5 GENERAL CONSIDERATIONS

5.1 Timing of Analyses

The Study Group, which consists of all site investigators, participating harvesters, and research coordinators, will meet annually to discuss the progress of the study and any problems encountered during the conduct of the trial. These reports will contain information on:

- Screening, Enrollment, and Retention
- Participant background characteristics at entry
- Data quality and protocol adherence

The groups charged with monitoring the various aspects of the study will be the Executive Committee, the Data Monitoring Committee (DMC), and the local site IRBs. These committees will meet at regular intervals according to the current Cooperative Studies Program guidelines: prior to the beginning of patient enrollment and at least every twelve months thereafter.

The final analysis will be performed on data which will have been documented as meeting the cleaning and approval requirements of CSP SOPs and after the finalization and approval of this SAP document.

5.2 Analysis Populations

Any participant requiring a non-emergent CABG will be considered for entry into the study. Participants who are hemodynamically unstable, have moderate to severe valvular disease or are unwilling or unable to provide informed consent will be excluded. The exact process for assigning the statuses will be defined and documented prior to final analyses along with any predefined reasons for eliminating a participant from a particular population.

Intent-to-Treat (ITT)

This population includes all participants who are randomized to the study. Participants will be assigned for analysis according to the group to which they were randomized, OVH or EVH. Participants will be categorized (in terms of their harvesting technique group assignment) based on their initial randomized group irrespective of conversion before surgery and will be included in the analyses irrespective of their status – completer or drop out of the study before completion. Analysis of all outcome measures – primary, secondary, and tertiary – will use the ITT population.

Safety

This population includes all participants who have signed informed consent. Adverse events are recorded for the duration of the participant's study participation and may include serious adverse events for up to one month after participation ends.

5.3 Missing Data and Imputations

Every effort will be made to minimize the occurrence of missing data, particularly for the primary and main secondary outcome measures. For the primary outcome (MACE), every effort will be made to contact the participants over the phone every three months until participant termination. In the event of a potential drop out, every effort will be made to capture the MACE data from the VA databases. The primary analyses will analyze the data as observed. When missing data are encountered in the analyses, a detailed sensitivity analysis may be conducted of the effects of various assumptions about the missing data and imputation methods utilized.

5.4 General Considerations

This section details general policies to be used for the statistical analyses. Departures from these general policies may be given in the specific detailed sections of this statistical analysis plan. When this situation occurs, the rules set forth in the specific section take precedence over the general policies. The following policies will be applied to all data presentations and analyses.

- All p-values will be rounded to 3 decimal places. All p-values that round to 0.000 will be presented as '<0.001' and p-values that round to 1.000 will be presented as '>0.999'. Any p-value $\leq \alpha$ will be considered statistically significant and will be marked with one asterisk (e.g., 0.026*).
- Summary statistics will consist of the number and percentage of responses in each category for discrete variables, and the mean, median, standard deviation (SD), minimum, and maximum for continuous variables.
- All mean and median values will be formatted to one more decimal place than the measured value. Standard deviation values will be formatted to two more decimal places than the measured value.

- All percentages will be rounded to one decimal place. The number and percentage of responses will be presented in the form XX (XX.X), where the percentage is in the parentheses. The decimal of the percentage may be dropped due to space constraints when creating a table.
- All listings will be sorted for presentation in order of treatment group, site number, participant number, and date of procedure or event.
- When necessary for analysis purposes, partial dates will be completed (i.e., turned into complete dates) using the most conservative approach.
- All analysis and summary tables will have the population sample size for each group or treatment group in the column heading.
- Version 9.3 of SAS® or higher will be the statistical software package used to produce all summaries, listings, statistical analyses, and graphs.
- Updated version of MedDRA will be used for adverse event and pre-treatment coding.
- The current version of the World Health Organization (WHO) drug dictionary will be used for the coding of medications.

5.5 Interim Monitoring

An independent oversight committee, a Data Monitoring Committee (DMC), will be monitoring study progress at predetermined time points over the entire duration of the study. The committee will receive analyses of the primary outcome measures and the important secondary outcome measures on a routine basis. In general, this committee meets at six to nine months after the start of subject recruitment and yearly thereafter. So, in total, this committee will meet maximum four times during the four years of the study duration. The committee will receive reports about three weeks prior to their annual meetings and at six monthly intervals in between the annual meetings. Since the primary outcome measure (MACE) is time-to-MACE event, sufficient data for DMC's first review will not be available until the study has been ongoing for at least 2 years. So there will be approximately 8 interim analyses of the primary outcome measure based on which the DMC will decide on study's continuation.

5.5.1 Data Monitoring Committee

An independent oversight committee called the Data Monitoring Committee (DMC) will monitor study progress. This committee meets on the same basic schedule as the Study Group and Executive Committee, i.e., at 6 to 9 months after the start of participant recruitment and yearly thereafter. Initially, the DMC will meet to become acquainted with the study and to establish monitoring guidelines.

The main responsibility of the DMC members is to make a recommendation to the Director of the Cooperative Studies Program on whether the study should continue or not based on the reviews of the progress reports submitted to them. The study could be recommended for termination due to poor recruitment, treatment differences so large that it would be possible to reach a final decision about the main question of the study, treatment differences so small that continuation would be irresponsible, or

due to safety concerns. The DMC also reviews the participating sites' performance in terms of recruitment, adherence to the protocol etc., and makes recommendations on them. Their final responsibility is to review all proposed protocol changes and suggested sub-protocols and to make recommendations in regards to their acceptability.

In order for the DMC to carry out its responsibilities, the CSPCC Study Team will provide the committee with a report approximately three weeks prior to their meetings. The report will consist of the tables describing number of participants enrolled, study progress, baseline demographics, as well as tables presenting outcome analyses. It is the responsibility of the CSPCC Study Team to provide the DMC with whatever information the committee feels that it needs to successfully monitor the study. Thus, additional tables will be added as required by the DMC. In addition to the reports for the yearly meetings, the DMC will also be provided with reports between meetings at 6-month intervals.

The patient characteristics would be presented by medical center and by harvesting technique group for the DMC. Significant imbalances of these patient characteristics between the harvesting technique groups may indicate a need to use these characteristics as covariates during the analysis of the outcome measures. Formal testing of the differences between groups will be done at the study's conclusion using appropriate statistical tests - analysis of variance technique will be used to test characteristics that are continuous in nature, while chi-square technique will be used for the test characteristics that are discrete in nature.

As with any clinical trial, the safety of the patient will be of utmost concern. Safety will be monitored closely during the course of the study. The DMC Report will include data on incidence of adverse events by treatment group. It will also include data on early terminations and treatment dropouts. The adverse event data will also be reported in the primary study manuscript. Data will be collected on adverse events throughout the study starting immediately after the patient signs the informed consent form.

5.5.1.1 Stopping Rules

In order for the DMC to make its recommendation for continuation of the study, it will be necessary for them to see the analyses for the primary outcome measure every time that the report is run and it is possible to calculate the primary outcome measure. Periodic monitoring of interim results can significantly affect the probability of making an incorrect decision. A number of formal techniques have been developed for interpreting interim results. At the organizational meeting, the DMC will select the technique that it wants to use to monitor the study. Suggested techniques are the Haybittle-Peto and Lan-DeMets group sequential boundaries. For the Haybittle-Peto method, a constant z-statistic is used as the monitoring boundary. The Lan-DeMets procedure produces decision boundaries that are quite conservative over the first several looks and then gradually converges to the nominal alpha levels as the final look is approached. The DMC will also analyze the safety data from the study to determine if the study should terminate. This will continue throughout the study.

5.5.2 Executive Committee

The Executive Committee is the management and decision-making body for the operational aspects of the study and will monitor the performance of participating medical centers and the quality of data collected. The Executive Committee will formulate publication plans and will oversee the publication and presentation of all data from the study. The Committee must grant permission before any study data may be used for presentation or publication. The Executive Committee is comprised of the Chairmen, PPCSP Biostatistician and Project Manager, LSIs and Subject Matter Experts.

6 SUMMARY OF STUDY DATA

All continuous variables will be summarised using the following descriptive statistics: n (non-missing sample size), mean, standard deviation, median, maximum and minimum. The frequency and percentages (based on the non-missing sample size) of observed levels will be reported for all categorical measures. In general, data listed will be sorted by medical center, treatment, and participant or by medical center and participant. Summary tables including treatment will be structured with either a column for each treatment in the order (OVH, EVH) or as rows for each treatment in the same order. Each table will be annotated with the total population size relevant to that table/treatment, including any missing observations.

6.1 Subject Disposition

Participant disposition will be summarized for all participants that signed the consent form and for the randomized population. The following data will be presented:

- The number of participants screened, eligible, consented, randomized, and expected to be randomized by medical center (TABLE 2). This table also provides the number of participants randomized to each group by center.
- The number of participants screened and randomized by month for each medical center (TABLE 3).
- A figure of participants randomized and expected by month (FIGURE 1).
- A listing that will include the reasons for exclusion of participants who discontinued from the study. These will include reasons for ineligibility (TABLE 4), non-enrollment (TABLE 5), and non-randomization (TABLE 6).
- Participant disposition by medical center for all participants (TABLE 7).

6.2 Demographics

Demographic characteristics at baseline will be summarized for all study participants. Demographic characteristics of all study participants will include age, gender, race/ethnicity, marital status, and education. The summary will include:

- The number and percentage of participants with each category of gender, race/ethnicity, marital status, and education (TABLE 8).
- The sample size, mean, median, SD, minimum and Maximum values for the following:
    - Age – calculated using the participant's birth date (birth date on Form 01).

Estimates of the baseline demographic characteristics will be presented for all study participants. Tables summarizing the important background characteristics by medical center and treatment will be prepared and submitted to the Study Group to provide an idea of the population being studied, and based on this information, comparisons of the participant characteristics among the centers will be possible.

6.3 Baseline Variables

6.3.1 Seattle Angina Questionnaire

The sample size, mean, median, SD and minimum and maximum values for the 5 component scores including angina stability, angina frequency, physical limitation, treatment satisfaction, and quality of life will reported for the baseline visit, overall by medical center and treatment (TABLE 9).

6.3.2 VR-12

The sample size, mean, median, SD, minimum, and maximum values for the PCS and MCS scores from the VR-12 will be presented for the baseline visit, overall by medical center and treatment (TABLE 10).

6.3.3 Conversion to Open Harvest Procedure

The conversion rate from EVH to OVH among randomized participants will be presented, overall and by medical center and harvester (TABLE 11), along with a list of reasons by center for the conversion (TABLE 12). The conversion rate, excluding conversions completed because the unanticipated graft required an additional vein will also be reported (TABLE 13).

6.3.4 Complexity of Coronary Artery Disease and Mortality and Morbidity Risks

The sample size, mean, median, SD, minimum, and maximum values for Syntax scores, indicating the complexity of coronary artery disease (CAD), will be presented, overall and by medical center, by MACE type, by age group, and by diabetes status as well as by treatment group (TABLES 14-17). Similar tables will be produced for the mortality and morbidity risk measures, VA Surgical Quality Improvement Program (VASQIP) and STS risk of mortality.

6.3.5 Bilateral Mammary Artery Grafting

The rate of bilateral mammary artery grafting among randomized participants will be presented, overall and by medical center (TABLE 18).

6.3.6 Leg Incision Healing and Severity of Incisional Leg Pain

The rate of leg incision infection and disturbance, along with severity of incisional leg pain (little or no impact, some impact, substantial impact, or severe impact) at discharge and 6-weeks will be assessed, overall and by medical center (TABLES 19-20).

6.4 Treatment Exposure

Treatment exposure is the vein harvesting technique to which study participants are randomized to, OVH vs. EVH. The number and percentages of participants randomized to either OVH or EVH will be reported.

- OVH – the traditional method of saphenectomy for CABG. It is performed under direct vision using a single long incision or, more commonly, multiple smaller incisions (referred to as "bridging" technique) along the course of the vein. This approach minimizes manipulation and direct trauma to the conduit but is associated with potential for discomfort and leg wound healing complications.
- EVH – is a minimally invasive procedure that was developed to eliminate the need for long incisions associated with OVH. EVH reduces the risk of wound infections and other leg wound complications but may be more traumatic to the conduit than OVH.

6.5 Study Endpoint

The rate of MACE and its components (all-cause mortality, nonfatal myocardial infarction, & repeat revascularization) will be reported, overall and by treatment (TABLE 21). Mean length of follow-up (in days) will also be reported by medical center and treatment (TABLE 22).

6.6 MACE by Harvester Experience

6.6.1 MACE by General Experience

The rate of MACE and its components (all-cause mortality, nonfatal myocardial infarction, & repeat revascularization) will be reported by harvesters' general experience: < 5 years, > 5 but < 10 years, and > 10 years. A separate table (similar to TABLE 21) will be provided for each level of experience.

6.6.2 MACE by EVH Experience

The rate of MACE and its components (all-cause mortality, nonfatal myocardial infarction, & repeat revascularization) will be reported by harvesters' EVH Experience: > 100 but < 500, > 500 but < 1,000, > 1,000 but < 2,000, and >2,000 endoscopic vein harvests completed. A separate table (similar to TABLE 21) will be provided for each level of experience.

6.6.3 MACE by OVH Experience

The rate of MACE and its components (all-cause mortality, nonfatal myocardial infarction, & repeat revascularization) will be reported by harvesters' OVH Experience: < 50, > 50 but < 100, > 100 but < 500, > 500 but < 1,000, >1,000 but < 2,000, and >2,000 endoscopic vein harvests completed. A separate table (similar to TABLE 21) will be provided for each level of experience.

6.7 Serious Adverse Events

Serious adverse event (SAE) incidence using MedDRA coding after randomization will be reported by body system and preferred term, overall and by treatment (TABLE 23).

6.8 Protocol Non-compliance

A listing of protocol non-compliance by medical center will be provided (TABLE 24).

7 STATISTICAL ANALYSES

7.1 Primary Outcome

The primary objective of this study is to investigate the impact of SVG harvesting techniques – OVH vs. EVH on MACE, a composite end point of all-cause mortality, nonfatal myocardial infarction and repeat revascularization, over the active follow-up period of the study postoperatively. The active follow-up time would begin with the first randomized patient and end after the last participant randomized has reached at least one-year of follow-up. Assuming the enrollment period is approximately three years, this would equate to approximately 4 to 4.5 years of active follow-up (in-clinic visit or contact by telephone carried out by site personnel), which will also be followed by two years of passive follow-up (carried out centrally by the chair's office staff using VA administrative databases). Thus, total follow-up time (active and passive) would be approximately 6.5 years. The primary outcome in the study is time (from index CABG surgery date) until the event of interest, MACE, or time-to-MACE during active follow-up (~4.5 years). Only the first event among participants will be considered as the MACE event.

To begin the analyses, the rate of MACE and its components (all-cause mortality, nonfatal myocardial infarction, & repeat revascularization) during active follow-up will be reported, both collectively and by SVG harvesting technique, OVH vs. EVH, considering only the first event so that components sum to total MACE (similar to TABLE 21). Survival analysis techniques will be employed to analyze the time-to-MACE data. Participants who are either lost-to-follow-up or did not experience a MACE event during the active follow-up period will be considered right-censored. Kaplan-Meier nonparametric survival estimates for risk of MACE will depict the unadjusted impact of SVG harvesting technique on MACE (FIGURE 2). Survival time represents time not experiencing a MACE. Tests of

equality (null hypothesis) of the survival function estimates across strata (OVH and EVH) will employ either the log-rank or Wilcoxon test. Multivariable survival analyses applying a Cox proportional hazards regression model will be performed to investigate the effect of SVG harvesting technique on time-to-MACE, adjusting for other potentially influential baseline characteristics, such as age, gender, harvester's experience, etc. (TABLE 25). Assumptions of the model (i.e., non-informative censoring, proportional hazards) will be checked. If assumptions appear to be violated, alternative methods will be explored (e.g., piecewise, time-varying covariates). A type I error rate of $\alpha = 0.05$ will be used throughout. Confidence intervals will be two-sided with a 95% confidence level.

The hypothesis for the primary aim of this study is:

> _H1_: *A significantly smaller proportion of CABG subjects with SVGs harvested by open technique will experience MACE post-surgery compared to CABG subjects with SVGs harvested by endoscopic technique during the active follow-up period.*

The hypothesis for the primary aim of this study will be supported when an increased risk of MACE is observed among participants randomized to EVH, confirmed with a hazards ratio (HR) > 1.00 for EVH vs. OVH and a significant corresponding *p-value* < α.

7.2 Secondary Outcomes

Secondary objectives of this study include investigating the impact of SVG harvesting techniques – OVH vs. EVH on MACE at one and three-years postoperatively and over the entire follow-up period (active and passive, ~6.5 years) of the study postoperatively. The proportion of participants experiencing a MACE (yes/no) in the first year of follow-up after index CABG surgery date will be calculated, both collectively and by SVG harvesting technique, OVH vs. EVH. These two proportions will be compared using Pearson's chi-square test. The proportion of participants experiencing a MACE (yes-1/no-0) in the three years of follow-up after index CABG surgery date will be similarly analyzed (TABLE 26).

Assessing MACE over the entire follow-up period (active and passive) of the study postoperatively will be analyzed similarly (TABLE 21, FIGURE 2, TABLE 25) to the primary outcome with now the outcome being time (from index CABG surgery date) until the event of interest, MACE, or time-to-MACE during the entire follow-up (~6.5 years). Events from both the active and passive follow-up periods will be considered, whereas the primary analysis only included events from the active follow-up period. Only the first event among participants will be considered as the MACE event.

The hypotheses for the secondary aims of this study are:

> _H2_: *One-year composite MACE rate will be 6 percentage points lower in the open harvesting group and three-year composite MACE rates will be at least 8-10 percentage points lower in the open vein harvesting group compared to the endoscopic vein harvesting group.*

<u>H3</u>: A significant smaller proportion of CABG subjects with SVGs harvested by open technique will experience MACE post-surgery compared to CABG subjects with SVGs harvested by endoscopic technique during the entire follow-up period.

The hypothesis H2 will be supported when the percent of participants experiencing MACE at one-year is 6% and at least 8% greater at three-years among those randomized to EVH vs. OVH, with a significant corresponding *p-value* < α observed for each test. The hypothesis H3 will be supported when an increased risk of MACE is observed among participants randomized to EVH, confirmed with a hazards ratio (HR) > 1.00 for EVH vs. OVH and a significant corresponding *p-value*. A total (recurrent) event analysis at various time points will also be explored comparing EVH and OVH.

7.3 Tertiary Outcomes

Tertiary objectives of this study include investigating the impact of SVG harvesting techniques – OVH vs. EVH on leg wound complication, patient satisfaction, and quality of life in addition to determining the role of vein harvester's experience on clinical outcomes.

All participants will be examined for post-operative complications of the leg wound from harvesting at discharge and at six weeks post-surgery. Post-operative leg wound complication status (yes-1/no-0) will be determined. The proportion of participants with leg wound complications will be computed for each treatment group and these proportions will be compared using Pearson's chi-square test (TABLE 27). The impact of confounding variables, such as BMI, diabetes status, and smoking status, on post-operative leg wound infection will be analyzed using multivariable logistic regression (TABLE 28). Surgical site infections at the vein harvest site according to the Center for Disease Control criteria will be explored.

Patient satisfaction will be assessed using the "Treatment Satisfaction" scale of the Seattle Angina Questionnaire. Participants will be categorized as "improved", "no change" and "worsened" at 6 weeks and 12 months compared to their baseline scores (TABLE 29). The proportion of participants in these three categories will be compared between the two groups using Pearson's chi-square test. The actual scores from these measures will also be used to compare harvesting techniques using analysis of covariance (ANCOVA) techniques, where the baseline (pre-surgery) scores will be used as a covariate (TABLE 30).

The occurrence of severe leg pain, due to incisions made during vein grafting, will be collected at discharge and 4-6 weeks post-CABG. The proportion of participants with severe pain (yes = pain severity rating score ≥ 3) at each time point will be compared across groups using Pearson's chi-square test (TABLE 31).

Quality of life scores using the PCS and MCS of the VR-12 and the "Quality of Life" scale of the Seattle Angina Questionnaire will be computed at baseline, six weeks, and 12 months post-surgery for participants in each SVG harvesting group. For each component of the VR-12 (PCS and MCS), mean changes from baseline at six weeks and 12 months will be computed and compared using ANCOVA (TABLE 32).

For each scale of the SAQ (angina stability, angina frequency, physical limitation, treatment satisfaction, & quality of life), participants will be categorized as "improved", "no change" and "worsened" at 6-weeks and 12 months compared to their baseline scores (TABLE 29). The proportion of participants in these three categories at each time point will be compared between the two groups using Pearson's chi-square test. The actual scores from these measures and that of the VR-12 MCS and PCS will be used to compare harvesting techniques using ANCOVA techniques, where the baseline (pre-surgery) scores will be used as a covariate (TABLE 30).

The hypotheses for the tertiary aims of this study are:

> *H4: Leg wound complications will be lower and satisfaction will be higher in the EVH group compared to OVH group.*
>
> *H5: Subjects' quality of life scores will be higher in the EVH group compared to the OVH group.*
>
> *H6: Exploratory Aim with no hypothesis specified.*

The leg wound complication component of hypothesis H4 will be supported when the percent of participants experiencing leg wound complications randomized to EVH is lower than that of participants randomized to OVH with a significant *p-value* observed. Higher patient satisfaction in the EVH group vs. OVH group will be supported by a significant effect for treatment when entered into the ANCOVA model and a positive coefficient for EVH vs. OVH. The hypothesis H5 will be supported by a significant effect for treatment when entered into the ANCOVA model and a positive coefficient for EVH vs. OVH for the outcomes of SAQ quality of life scale, VR-12 PCS, and VR-12 MCS.

8 SERIOUS ADVERSE EVENTS

In this study, information on all serious adverse events (SAEs) will be collected and recorded. Incidence of SAEs will be summarized for each treatment group by body system and MedDRA term. The number and percentage of participants with each body system and MedDRA term will be presented for each treatment group.

9 STATISTICAL PROGRAM VALIDATION PLAN

Perry Point Work Instruction (WI) 202 – Validation Plan for SAS Statistical Programs will serve as the validation plan for validation of the statistical programs created for the analyses of data collected during this study.

REFERENCES

Becker J, Saris-Baglama RN, Kosinski M, et al. The Pain Impact Questionnaire (PIQ-6): A User's Guide. QualityMetric Incorporated 2005: Lincoln, RI.

Desai P, Kiani S, Thiruvanthan N, et al. Impact of the learning curve for endoscopic vein harvest on conduit quality and early graft patency. Ann Thorac Surg 2011; 91:1385-1392.

Iqbal SU, Rogers W, Selim A, Qian S, Lee A, Ren XS, Rothendler J, Miller D, Kazis LE (2009). The Veterans Rand 12 item health survey (VR-12): What it is and how it is used. Center for Health Quality, Outcomes, and Economic Research, A Health Services Research and Development Center of Excellence: VA Medical Center, Bedford, MA.

Shroyer AL, Grover FL, Hattler B, et al. On-pump versus off-pump coronary-artery bypass surgery. N Engl J Med 2009; 361(19):1827-37.

Wilson APR, Sturridge MF, Treasure T, Gruneberg RN. A scoring method (asepsis) for postoperative wound infections for use in clinical trials of antibiotic prophylaxis. The Lancet 1986; 327(8476): 311-312.

APPENDIX 1: TABLES AND FIGURES

TABLE 2: Number of Participants Screened, Eligible, Consented, Randomized and Expected Randomized, Total and by Medical Center

	Alb. (501)	Boston (523)	Cle. (541)	Miami (546)	Durham (558)	Gaines. (573)	Houston (580)	Minn. (618)	New York (630)	Asheville (637)	Pitts. (646)	Portland (648)	San Fran. (662)	Tampa (673)	Tucson (678)	Milw. (695)	Total
**Number Screened**																	
Ineligible																	
Percent Ineligible (of Screened)																	
**Number Eligible**																	
Not Consented																	
Percent Not Consented (of Eligible)																	
**Number Consented**																	
Not Randomized																	
Percent Not Randomized (of Consented)																	
**Number Randomized**																	
Expected																	
Percent of Expected																	
**Randomized to OVH**																	
**Randomized to EVH**																	

TABLE 3: Participants Screened and Randomized by Month, Total and by Medical Center

Month	Albuquerque (501)		Boston (523)		Cleveland (541)		Miami (546)		Durham (558)		Gainesville (573)	
	Screened	Randomized	Screened	Randomized	Screened	Randomized	Screened	Randomized	Screened	Randomized	Screened	Randomized
Month 1												
Month 2												
Month 3												
Month 4												
Month 5												
Month 6												
Month 7												
Month 8												
Month 9												
Month 10												
Month 11												
Month 12												
Month 13												
Month 14												
Month 15												
Month 16												
Month 17												
Month 18												
Month 19												
Month 20												
Month 21												
Month 22												
Month 23												
Month 24												
Month 25												
Month 26												
Month 27												
Month 28												
Month 29												
Month 30												
Month 31												
Month 32												
Month 33												
Month 34												
Month 35												
Month 36												
Month 37												
Month 38												

Month	Houston (580)		Minneapolis (618)		New York (630)		Asheville (637)		Pittsburgh (646)		Portland (648)	
	Screened	Randomized	Screened	Randomized	Screened	Randomized	Screened	Randomized	Screened	Randomized	Screened	Randomized
Month 1												
Month 2												
Month 3												
Month 4												
Month 5												
Month 6												
Month 7												
Month 8												
Month 9												
Month 10												
Month 11												
Month 12												
Month 13												
Month 14												
Month 15												
Month 16												
Month 17												
Month 18												
Month 19												
Month 20												
Month 21												
Month 22												
Month 23												
Month 24												
Month 25												
Month 26												
Month 27												
Month 28												
Month 29												
Month 30												
Month 31												
Month 32												
Month 33												
Month 34												
Month 35												
Month 36												
Month 37												
Month 38												

**Month**	**San Francisco (662)**		**Tampa (673)**		**Tucson (678)**		**Milwaukee (695)**		**Total**	
	Screened	Randomized	Screened	Randomized	Screened	Randomized	Screened	Randomized	Screened	Randomized
Month 1										
Month 2										
Month 3										
Month 4										
Month 5										
Month 6										
Month 7										
Month 8										
Month 9										
Month 10										
Month 11										
Month 12										
Month 13										
Month 14										
Month 15										
Month 16										
Month 17										
Month 18										
Month 19										
Month 20										
Month 21										
Month 22										
Month 23										
Month 24										
Month 25										
Month 26										
Month 27										
Month 28										
Month 29										
Month 30										
Month 31										
Month 32										
Month 33										
Month 34										
Month 35										
Month 36										
Month 37										
Month 38										

FIGURE 1: Randomization Activities Based on Number of Active Centers per Month

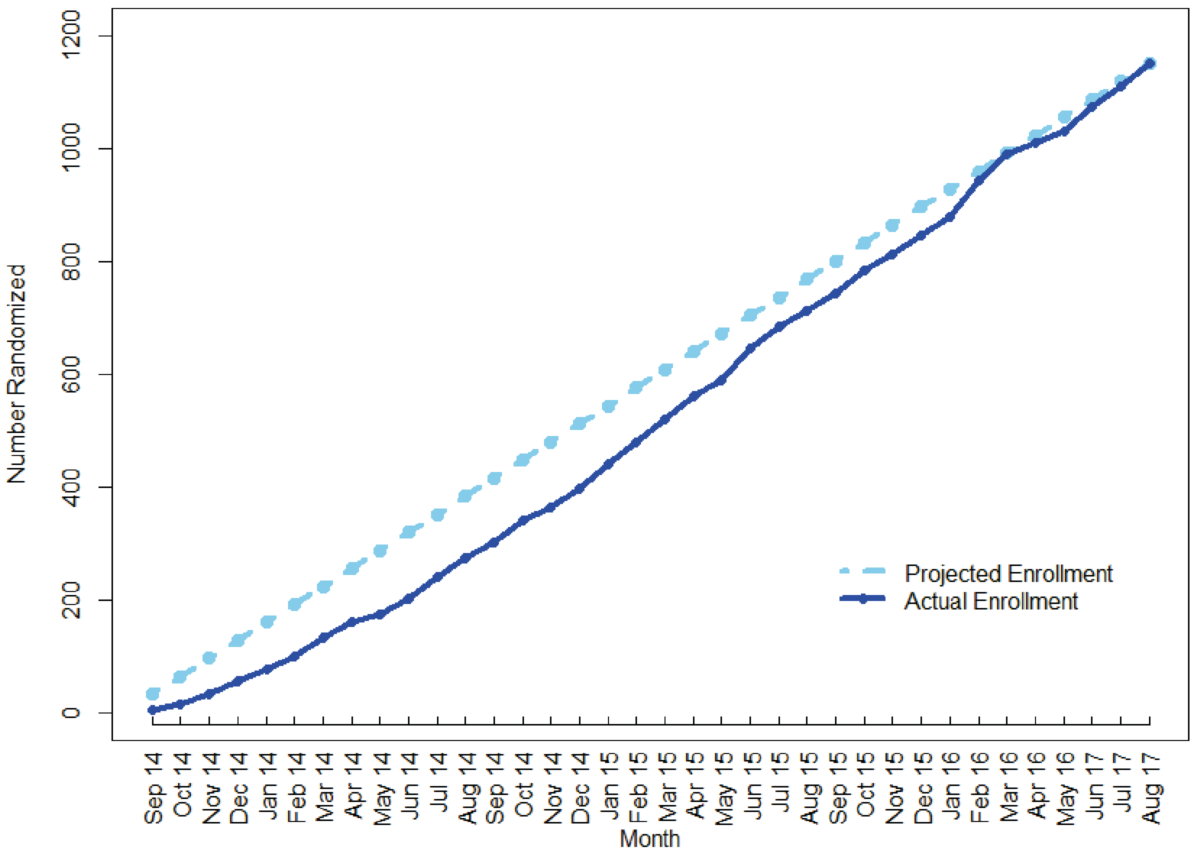

TABLE 4: Reasons for Ineligibility, Total and by Medical Center

	Alb. (501)	Boston (523)	Cle. (541)	Miami (546)	Durham (558)	Gaines. (573)	Houston (580)	Minn. (618)	New York (630)	Asheville (637)	Pitts. (646)	Portland (648)	San Fran. (662)	Tampa (673)	Tucson (678)	Milw. (695)	Total
**Not elective or urgent CABG**																	
**Not median sternotomy approach**																	
**No coronary bypass planned using saphenous vein graft for conduit**																	
**No experienced EVH/OVH harvester and/or participating surgeon available for procedure**																	
**Combined valve procedure planned**																	
**Moderate or severe valve disease**																	
**Hemodynamically unstable or in cardiogenic shock**																	
**Enrolled in another therapeutic or interventional study**																	
**Off-pump CABG procedure planned**																	
**Limited life expectancy less than 1 year**																	
**History of lower extremities venous stripping or ligation**																	
**Inability to provide informed consent**																	
**Total**																	

TABLE 5: Reasons for Non-Enrollment, Total and by Medical Center

	Alb. (501)	Boston (523)	Cle. (541)	Miami (546)	Durham (558)	Gaines. (573)	Houston (580)	Minn. (618)	New York (630)	Asheville (637)	Pitts. (646)	Portland (648)	San Fran. (662)	Tampa (673)	Tucson (678)	Milw. (695)	Total
**Subject refused to sign Informed Consent**																	
**Eligibility status changed**																	
**Site surgeon concerned about resources needed for on-site follow-up**																	
**No reliable method of follow-up contact with the subject**																	
**Subject prefers open vein harvest**																	
**Subject prefers endoscopic vein harvest**																	
**Subject preferred non-enrollment for reasons other than vein harvest preference**																	
**Surgeon prefers open vein harvest for subject**																	
**Surgeon prefers endoscopic vein harvest for subject**																	
**Surgeon preferred non-enrollment for reasons other than vein harvest preference**																	
**Total**																	

TABLE 6: Reasons for Non-Randomization, Total and by Medical Center

	Alb. (501)	Boston (523)	Cle. (541)	Miami (546)	Durham (558)	Gaines. (573)	Houston (580)	Minn. (618)	New York (630)	Asheville (637)	Pitts. (646)	Portland (648)	San Fran. (662)	Tampa (673)	Tucson (678)	Milw. (695)	Total
**Eligibility status changed**																	
**Subject changed mind**																	
**Surgeon changed mind**																	
**Other reason**																	
**Total**																	

TABLE 7: Status of Randomized Participants, Total and by Medical Center

Center	Randomized	Completed Week 6	In Active Follow-up	Early Termination*	Completers
**Albuquerque (501)**					
**Boston (523)**					
**Cleveland (541)**					
**Miami (546)**					
**Durham (558)**					
**Gainesville (573)**					
**Houston (580)**					
**Minneapolis (618)**					
**New York (630)**					
**Asheville (637)**					
**Pittsburgh (646)**					
**Portland (648)**					
**San Francisco (662)**					
**Tampa (673)**					
**Tucson (678)**					
**Milwaukee (695)**					
**Total**					

**Reasons for early termination include: n (%) voluntarily withdrew, n (%) lost to follow-up, n (%) Other, and n (%) died.*

TABLE 8: Summary of Demographics, Total and by Medical Center

		Albuquerque (501)	Boston (523)	Cleveland (541)	Miami (546)	Durham (558)	Gainesville (573)
**Age**	n						
	Mean (SD)						
	Median						
	Min, Max						
**Gender**	n						
Male	n (%)						
Female	n (%)						
**Race**	n						
American Indian or Alaskan Native	n (%)						
Asian or Pacific Islander	n (%)						
Black, not of Hispanic origin	n (%)						
Hispanic	n (%)						
White, not of Hispanic origin	n (%)						
Other	n (%)						
**Marital Status**	n						
Married/remarried	n (%)						
Divorced	n (%)						
Separated	n (%)						
Widowed	n (%)						
Never married	n (%)						
**Education**	n						
Completed graduate/professional training	n (%)						
Standard college/university graduate	n (%)						
Partial college training	n (%)						
High school graduate/GED	n (%)						
< High school	n (%)						

		Houston (580)	Minn. (618)	New York (630)	Asheville (637)	Pitts. (646)	Portland (648)
**Age**	n						
	Mean (SD)						
	Median						
	Min, Max						
**Gender**	n						
Male	n (%)						
Female	n (%)						
**Race**	n						
American Indian or Alaskan Native	n (%)						
Asian or Pacific Islander	n (%)						
Black, not of Hispanic origin	n (%)						
Hispanic	n (%)						
White, not of Hispanic origin	n (%)						
Other	n (%)						
**Marital Status**	n						
Married/remarried	n (%)						
Divorced	n (%)						
Separated	n (%)						
Widowed	n (%)						
Never married	n (%)						
**Education**	n						
Completed graduate/professional training	n (%)						
Standard college/university graduate	n (%)						
Partial college training	n (%)						
High school graduate/GED	n (%)						
< High school	n (%)						

		San Fran. (662)	Tampa (673)	Tucson (678)	Milw. (695)	Total	OVH	EVH
**Age**	n							
	Mean (SD)							
	Median							
	Min, Max							
**Gender**	n							
Male	n (%)							
Female	n (%)							
**Race**	n							
American Indian or Alaskan Native	n (%)							
Asian or Pacific Islander	n (%)							
Black, not of Hispanic origin	n (%)							
Hispanic	n (%)							
White, not of Hispanic origin	n (%)							
Other	n (%)							
**Marital Status**	n							
Married/remarried	n (%)							
Divorced	n (%)							
Separated	n (%)							
Widowed	n (%)							
Never married	n (%)							
**Education**	n							
Completed graduate/professional training	n (%)							
Standard college/university graduate	n (%)							
Partial college training	n (%)							
High school graduate/GED	n (%)							
< High school	n (%)							

TABLE 9: Seattle Angina Questionnaire: [*Component scale here*] at Baseline, by Medical Center and Treatment

Center	OVH						EVH						Total					
	N	Mean	Std Dev	Median	Min	Max	N	Mean	Std Dev	Median	Min	Max	N	Mean	Std Dev	Median	Min	Max
Albuquerque (501)																		
Boston (523)																		
Cleveland (541)																		
Miami (546)																		
Durham (558)																		
Gainesville (573)																		
Houston (580)																		
Minneapolis (618)																		
New York (630)																		
Asheville (637)																		
Pittsburgh (646)																		
Portland (648)																		
San Francisco (662)																		
Tampa (673)																		
Tucson (678)																		
Milwaukee (695)																		
Total																		

TABLE 10: Veterans Rand 12 Item Health Survey: [*Component scale here*] at Baseline, by Medical Center and Treatment

	OVH						EVH						Total					
Center	N	Mean	Std Dev	Median	Min	Max	N	Mean	Std Dev	Median	Min	Max	N	Mean	Std Dev	Median	Min	Max
Albuquerque (501)																		
Boston (523)																		
Cleveland (541)																		
Miami (546)																		
Durham (558)																		
Gainesville (573)																		
Houston (580)																		
Minneapolis (618)																		
New York (630)																		
Asheville (637)																		
Pittsburgh (646)																		
Portland (648)																		
San Francisco (662)																		
Tampa (673)																		
Tucson (678)																		
Milwaukee (695)																		
Total																		

TABLE 11: Conversion to Open Harvest Procedure, by Medical Center and Harvester, in Patients Randomized to Endoscopic Harvest Procedure.

		Conversion		Randomized
Center	Harvester	N	%	N
Albuquerque (501)	01			
	Total			
Boston (523)	01			
	02			
	Total			
Cleveland (541)	01			
	Total			
Miami (546)	01			
	02			
	03			
	Total			
Durham (558)	01			
	02			
	03			
	Total			
Gainesville (573)	01			
	02			
	Total			
Houston (580)	01			
	02			
	03			
	Total			
Minneapolis (618)	01			
	02			
	Total			
New York (630)	01			
	Total			

**Asheville (637)**	01			
	02			
	Total			
**Pittsburgh (646)**	01			
	Total			
**Portland (648)**	01			
	02			
	03			
	Total			
**San Francisco (662)**	01			
	02			
	Total			
**Tampa (673)**	01			
	02			
	Total			
**Tucson (678)**	02			
	Total			
**Milwaukee (695)**	03			
	04			
	05			
	Total			
**Total**	**Total**			

TABLE 12: Reasons for Conversion to Open Harvest Procedure, by Medical Center

Center	Bleeding	Injury to SVG	Unacceptable EVH procedure time	Insufficient amount of usable vein	Unanticipated graft needed	Harvester unable to locate vein	Other	Total
**Albuquerque (501)**								
**Boston (523)**								
**Cleveland (541)**								
**Miami (546)**								
**Durham (558)**								
**Gainesville (573)**								
**Houston (580)**								
**Minneapolis (618)**								
**New York (630)**								
**Asheville (637)**								
**Pittsburgh (646)**								
**Portland (648)**								
**San Francisco (662)**								
**Tampa (673)**								
**Tucson (678)**								
**Milwaukee (695)**								
**Total**								

TABLE 13: Conversion to Open Harvest Procedure, by Medical Center and Harvester, Excluding Conversions Completed Because Unanticipated Graft Required Additional Vein, in Patients Randomized to Endoscopic Harvest Procedure.

		Conversion		Randomized
**Center**	**Harvester**	**N**	**%**	**N**
**Albuquerque (501)**	01			
	Total			
**Boston (523)**	01			
	02			
	Total			
**Cleveland (541)**	01			
	Total			
**Miami (546)**	01			
	02			
	03			
	Total			
**Durham (558)**	01			
	02			
	03			
	Total			
**Gainesville (573)**	01			
	02			
	Total			
**Houston (580)**	01			
	02			
	03			
	Total			
**Minneapolis (618)**	01			
	02			
	Total			
**New York (630)**	01			

	Total			
**Asheville (637)**	01			
	02			
	Total			
**Pittsburgh (646)**	01			
	Total			
**Portland (648)**	01			
	02			
	03			
	Total			
**San Francisco (662)**	01			
	02			
	Total			
**Tampa (673)**	01			
	02			
	Total			
**Tucson (678)**	02			
	Total			
**Milwaukee (695)**	03			
	04			
	05			
	Total			
**Total**	**Total**			

TABLE 14: Mean [SYNTAX Score, VASQIP Patient Risk Calculation, STS Risk of Mortality], Total and by Medical Center and Treatment

	OVH						EVH						Total					
Center	N	Mean	Std Dev	Median	Min	Max	N	Mean	Std Dev	Median	Min	Max	N	Mean	Std Dev	Median	Min	Max
Albuquerque (501)																		
Boston (523)																		
Cleveland (541)																		
Miami (546)																		
Durham (558)																		
Gainesville (573)																		
Houston (580)																		
Minneapolis (618)																		
New York (630)																		
Asheville (637)																		
Pittsburgh (646)																		
Portland (648)																		
San Francisco (662)																		
Tampa (673)																		
Tucson (678)																		
Milwaukee (695)																		
Total																		

TABLE 15: Mean [SYNTAX Score, VASQIP Patient Risk Calculation, STS Risk of Mortality], by MACE and Treatment

	Harvesting Technique	Yes						No					
		N	Mean	Std Dev	Median	Min	Max	N	Mean	Std Dev	Median	Min	Max
**Any MACE**	OVH												
	EVH												
	**Total**												
**Death**	OVH												
	EVH												
	**Total**												
**Myocardial Infarction**	OVH												
	EVH												
	**Total**												
**Repeat Revascularization**	OVH												
	EVH												
	**Total**												

TABLE 16: Mean [SYNTAX Score, VASQIP Patient Risk Calculation, STS Risk of Mortality], by Age Group and Treatment

Age	OVH						EVH						Total					
	N	Mean	Std Dev	Median	Min	Max	N	Mean	Std Dev	Median	Min	Max	N	Mean	Std Dev	Median	Min	Max
< 50 years																		
50 to 59 years																		
60 to 69 years																		
70 to 79 years																		
> 79 years																		
Total																		

TABLE 17: Mean [SYNTAX Score, VASQIP Patient Risk Calculation, STS Risk of Mortality], by Diabetes Status and Treatment

Diabetes	OVH						EVH						Total					
	N	Mean	Std Dev	Median	Min	Max	N	Mean	Std Dev	Median	Min	Max	N	Mean	Std Dev	Median	Min	Max
No																		
Yes																		
Total																		

TABLE 18: Bilateral Mammary Artery Grafting, Total and by Medical Center and Treatment

	OVH				EVH				Total			
	Yes		No		Yes		No		Yes		No	
Center	N	%	N	%	N	%	N	%	N	%	N	%
Albuquerque (501)												
Boston (523)												
Cleveland (541)												
Miami (546)												
Durham (558)												
Gainesville (573)												
Houston (580)												
Minneapolis (618)												
New York (630)												
Asheville (637)												
Pittsburgh (646)												
Portland (648)												
San Francisco (662)												
Tampa (673)												
Tucson (678)												
Milwaukee (695)												
Total												

TABLE 19: Leg Incision Healing, Total and by Medical Center and Treatment

	OVH						EVH						Total					
	No Disturbance		Disturbance		Infection		No Disturbance		Disturbance		Infection		No Disturbance		Disturbance		Infection	
**Center**	**N**	**%**	**N**	**%**	**N**	**%**	**N**	**%**	**N**	**%**	**N**	**%**	**N**	**%**	**N**	**%**	**N**	**%**
**Albuquerque (501)**																		
**Boston (523)**																		
**Cleveland (541)**																		
**Miami (546)**																		
**Durham (558)**																		
**Gainesville (573)**																		
**Houston (580)**																		
**Minneapolis (618)**																		
**New York (630)**																		
**Asheville (637)**																		
**Pittsburgh (646)**																		
**Portland (648)**																		
**San Francisco (662)**																		
**Tampa (673)**																		
**Tucson (678)**																		
**Milwaukee (695)**																		
**Total**																		

TABLE 20: Severity of Incisional Leg Pain at [Discharge, 6-weeks Postoperative], Total and by Medical Center and Treatment

Center	Harvesting Technique	Little or no impact		Some impact		Substantial impact		Severe impact		Total
		N	%	N	%	N	%	N	%	N
**Albuquerque (501)**	OVH									
	EVH									
	**Total**									
**Boston (523)**	OVH									
	EVH									
	**Total**									
**Cleveland (541)**	OVH									
	EVH									
	**Total**									
**Miami (546)**	OVH									
	EVH									
	**Total**									
**Durham (558)**	OVH									
	EVH									
	**Total**									
**Gainesville (573)**	OVH									
	EVH									
	**Total**									
**Houston (580)**	OVH									
	EVH									
	**Total**									
**Minneapolis (618)**	OVH									
	EVH									
	**Total**									
**New York (630)**	OVH									
	EVH									

	**Total**									
**Asheville (637)**	OVH									
	EVH									
	**Total**									
**Pittsburgh (646)**	OVH									
	EVH									
	**Total**									
**Portland (648)**	OVH									
	EVH									
	**Total**									
**San Francisco (662)**	OVH									
	EVH									
	**Total**									
**Tampa (673)**	OVH									
	EVH									
	**Total**									
**Tucson (678)**	OVH									
	EVH									
	**Total**									
**Milwaukee (695)**	OVH									
	EVH									
	**Total**									
**Total**	OVH									
	EVH									
	**Total**									

TABLE 21: Major Adverse Cardiac Events, Overall and by Treatment

Center	Harvesting Technique	Yes		No		Total	
		N	%	N	%	N	%
**Any Major Adverse Cardiac Event**	OVH						
	EVH						
	**Total**						
**Death**	OVH						
	EVH						
	**Total**						
**Myocardial Infarction**	OVH						
	EVH						
	**Total**						
**Repeat Revascularization**	OVH						
	EVH						
	**Total**						

TABLE 22: Mean Length of Follow-up in Days, by Medical Center and Treatment

	OVH						EVH						Total					
**Center**	**N**	**Mean**	**Std Dev**	**Median**	**Min**	**Max**	**N**	**Mean**	**Std Dev**	**Median**	**Min**	**Max**	**N**	**Mean**	**Std Dev**	**Median**	**Min**	**Max**
**Albuquerque (501)**																		
**Boston (523)**																		
**Cleveland (541)**																		
**Miami (546)**																		
**Durham (558)**																		
**Gainesville (573)**																		
**Houston (580)**																		
**Minneapolis (618)**																		
**New York (630)**																		
**Asheville (637)**																		
**Pittsburgh (646)**																		
**Portland (648)**																		
**San Francisco (662)**																		
**Tampa (673)**																		
**Tucson (678)**																		
**Milwaukee (695)**																		
**Total**																		

TABLE 23: Cumulative Serious Adverse Event Incidence using MedDRA coding in Randomized Participants, by Body System and Preferred Term

	OVH				EVH				Total			
	Participants		Events		Participants		Events		Participants		Events	
Body System and Preferred Terms	N	%	N	%	N	%	N	%	N	%	N	%
Cardiac Disorders												
Cardiac failure congestive												
…and so on												

TABLE 24: Protocol Non-compliance

Center	Rate of Reporting	Participant ID	Protocol Non-Compliance Code	Date of Deviation
Albuquerque (501)				
…				
… and so on				

FIGURE 2: Kaplan-Meier Survival Curves

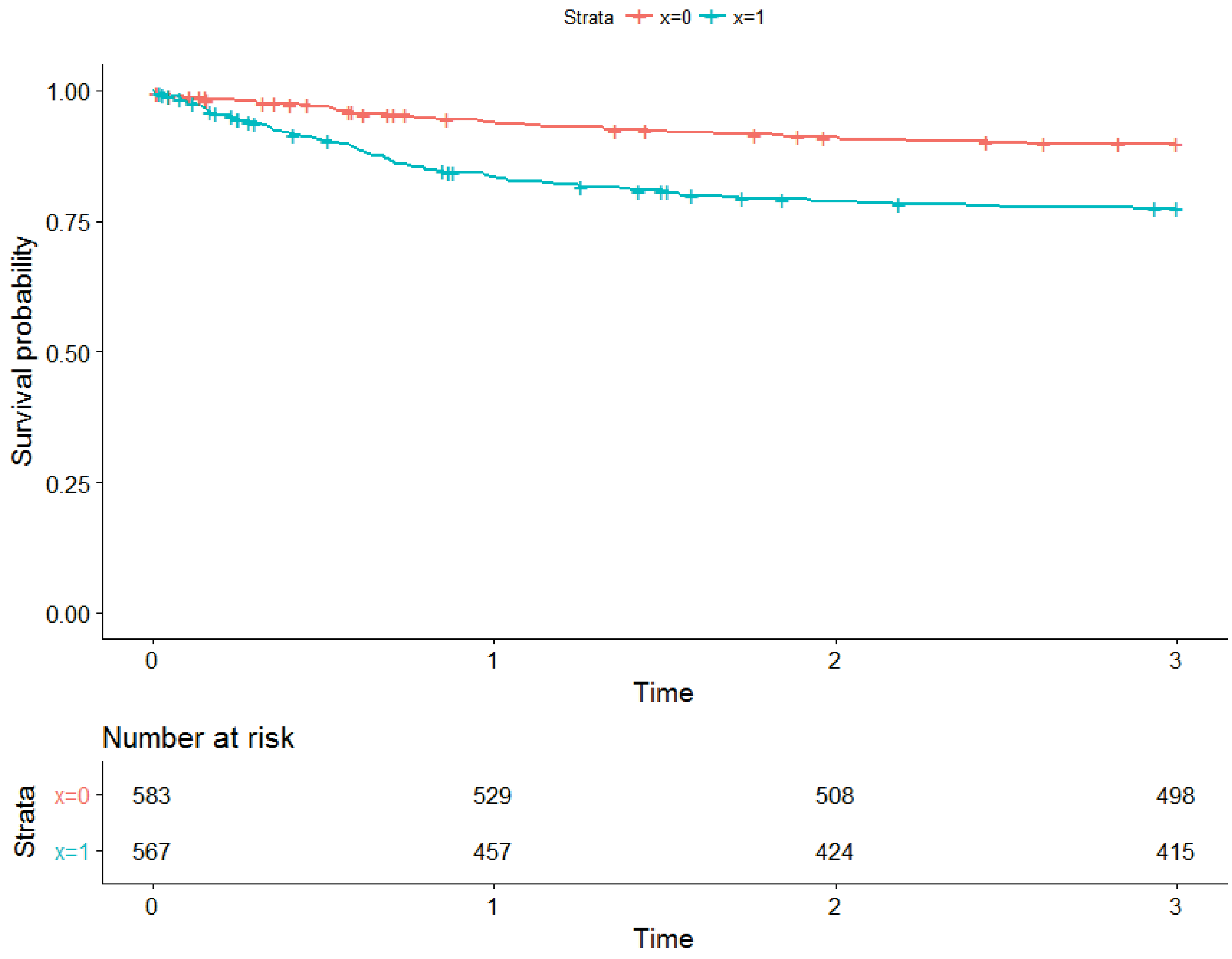

TABLE 25: MACE, Active Follow-up: Results of Multivariable Cox Proportional Hazards Regression Model

Parameter	DF	Parameter Estimate	Standard Error	Chi-Square Test Statistic	p-value	Hazard Ratio	95% Hazard CI
**EVH (vs. OVH)**							
**Covariate 1**							
**…**							
**… and so on**							

TABLE 26: Major Adverse Cardiac Events at 1-year and 3-year, by Treatment

		OVH		EVH		Total		
		N	%	N	%	N	%	p-value
**1-Year**	**MACE**							
	Death							----
	MI							----
	RR							----
**3-Year**	**MACE**							
	Death							----
	MI							----
	RR							----

TABLE 27: Leg Incision Healing by Treatment

	OVH		EVH		Total		
	N	%	N	%	N	%	p-value
**No Disturbance**							----
**Complication (Any Disturbance)**							

TABLE 28: Leg Incision Healing by Treatment: Results of Multivariable Logistic Regression Model

Parameter	DF	Parameter Estimate	Standard Error	Chi-Square Test Statistic	p-value	Odds Ratio	95% CI
**EVH (vs. OVH)**							
**Covariate 1**							
**…**							
**… and so on**							

TABLE 29: Seattle Angina Questionnaire: Change from Baseline by Treatment

			OVH		EVH		Total		
			N	%	N	%	N	%	p-value
[Component scale]	6-week	**Improved**							
		**No Change**							----
		**Worsened**							-----
	12-month	**Improved**							
		**No Change**							----
		**Worsened**							-----

* No change = (mean score change < 5)

TABLE 30: Seattle Angina Questionnaire: Results of ANCOVA Model

		Parameter	DF	Parameter Estimate	Standard Error	t-Test Statistic	p-value
[Component scale]	*[6-week /12-month]*	**Intercept**					
		**Baseline Score**					
		**EVH (vs. OVH)**					
		**Interaction (if needed)**					

TABLE 31: Impact and Severity of Incisional Leg Pain at by Treatment

		OVH		EVH		Total		
		N	%	N	%	N	%	p-value
	**Little or no impact** (impact score ≤ 50)							
	**Some impact** (50 < impact score ≤ 57)							----
**Discharge**	**Substantial impact** (57 < impact score ≤ 63)							-----
	**Severe impact** (impact score > 63)							----
	**Severe (per severity rating)** (Score of 3 or above)							
	**Little or no impact** (impact score ≤ 50)							
	**Some impact** (50 < impact score ≤ 57)							----
**6-Weeks**	**Substantial impact** (57 < impact score ≤ 63)							-----
	**Severe impact** (impact score > 63)							----
	**Severe (per severity rating)** (Score of 3 or above)							

TABLE 32: Veterans Rand 12 Item Health Survey: [*Component scale here*] by Medical Center and Treatment

	OVH						EVH						Total						p-value
	N	Mean	SD	Median	Min	Max	N	Mean	SD	Median	Min	Max	N	Mean	SD	Median	Min	Max	
**Baseline**																			
**6 weeks**																			
**12 months**																			
**6-week change**																			
**12-month change**																			
