# Supplementary material for: Costs of Endoscopic vs Open Vein Harvesting for Coronary Artery Bypass Grafting: A Secondary Analysis of the REGROUP Trial
Source: JAMA Netw Open. 2022 Jun 21;5(6):e2217686. doi: 10.1001/jamanetworkopen.2022.17686 (PMC9214587; doi:10.1001/jamanetworkopen.2022.17686)
Supplement: Supplement 2. — eTable 1. Subgroup Analyses for 6 Chronic Conditions eTable 2. Adjusted Follow-up Costs From GEE Models With a Log Link and a Gamma Error Distribution eTable 3. Number of Participants Followed Up by 90-Day Follow-up Period [file jamanetwopen-e2217686-s002.pdf]

Supplementary Online Content

Wagner TH, Hattler B, Stock EM, et al. Costs of endoscopic vs open vein harvesting for coronary artery bypass grafting: a secondary analysis of the REGROUP trial. *JAMA Netw Open*. 2022;5(6):e2217686. doi:10.1001/jamanetworkopen.2022.17686

eTable 1. Subgroup Analyses for 6 Chronic Conditions

eTable 2. Adjusted Follow-up Costs From GEE Models With a Log Link and a Gamma Error Distribution

eTable 3. Number of Participants Followed Up by 90-Day Follow-up Period

This supplementary material has been provided by the authors to give readers additional information about their work.

© 2022 Wagner TH et al. *JAMA Network Open*.

eTable 1. Subgroup Analyses for 6 Chronic Conditions

	Discharge Costs ($)		Follow-up Costs ($)	
	**Mean difference**	**SE**	**Mean difference**	**SE**
Diabetes	-151.01	(4,962.13)	1,141.87	(955.00)
Hypertension	10,209.47	(8,308.75)	678.49	(931.56)
Hyperlipidemia	10,476.73	(7,220.05)	-389.82	(815.18)
Depression	-7,250.83	(5,640.49)	920.69	(644.37)
Renal	-6,508.59	(7,263.01)	414.96	(824.82)
Liver	-2,202.84	(13,137.41)	1,865.94	(1,483.26)

The mean difference represents the interaction between EVH and the chronic condition
None of the mean differences were statistically significant
Statistical models control for follow-up time period, VA hospital site, age, sex, race, diabetes, hypertension, hyperlipidemia, depression, chronic kidney disease, and chronic liver disease. The analysis includes a person random effect.

© 2022 Wagner TH et al. *JAMA Network Open*.

eTable 2. Adjusted Follow-up Costs From GEE Models With a Log Link and a Gamma Error Distribution

	Open Harvest			Endoscopic Harvest			
	Adjusted Estimate	95% CI		Adjusted Estimate	95% CI		Mean difference
VA provided care							
Total Costs	4,060	3,769	4,350	4,346	4,019	4,672	286
Inpatient Costs	479	311	646	549	356	742	70
Med/surg	433	288	579	469	311	626	36
Outpatient costs							
Total	3,627	3,356	3,897	3,853	3,550	4,156	226
Med/surg	1,985	1,916	2,054	2,049	1,978	2,120	64
Pharmacy	640	532	749	667	563	771	27
VA Purchased care	1,461	1,218	1,704	1,579	1,313	1,845	118

Statistical models control for follow-up time period, VA hospital site, age, sex, race, diabetes, hypertension, hyperlipidemia, depression, chronic kidney disease, and chronic liver disease. The analysis includes a person random effect.

None of the mean differences are statistically significant

© 2022 Wagner TH et al. *JAMA Network Open*.

eTable 3. Number of Participants Followed Up by 90-Day Follow-up Period

90 day period	n	percent	cum percent
1	1,149	4.63	4.63
2	1,149	4.63	9.26
3	1,149	4.63	13.88
4	1,149	4.63	18.51
5	1,149	4.63	23.14
6	1,149	4.63	27.77
7	1,149	4.63	32.39
8	1,149	4.63	37.02
9	1,149	4.63	41.65
10	1,149	4.63	46.28
11	1,149	4.63	50.91
12	1,149	4.63	55.53
13	1,149	4.63	60.16
14	1,149	4.63	64.79
15	1,125	4.53	69.32
16	1,082	4.36	73.68
17	1,028	4.14	77.82
18	965	3.89	81.71
19	890	3.58	85.29
20	803	3.23	88.53
21	722	2.91	91.43
22	622	2.51	93.94
23	511	2.06	96.00
24	393	1.58	97.58
25	300	1.21	98.79
26	204	0.82	99.61
27	79	0.32	99.93
28	16	0.06	99.99
29	2	0.01	100.00

Note: the n is based on participants eligible for follow-up based on timing of enrollment.

© 2022 Wagner TH et al. *JAMA Network Open*.
