# Supplementary material for: Costs of Endoscopic vs Open Vein Harvesting for Coronary Artery Bypass Grafting: A Secondary Analysis of the REGROUP Trial
Source: JAMA Netw Open. 2022 Jun 21;5(6):e2217686. doi: 10.1001/jamanetworkopen.2022.17686 (PMC9214587; doi:10.1001/jamanetworkopen.2022.17686)
Supplement: Supplement 3. — Data Sharing Statement [file jamanetwopen-e2217686-s00.pdf]

Data Sharing Statement

Wagner. Costs of Endoscopic vs Open Vein Harvesting for Coronary Artery Bypass Grafting. *JAMA Netw Open*. Published June 21, 2022. doi:10.1001/jamanetworkopen.2022.17686

Data
Data available: No

Additional Information
Explanation for why data not available: The data are the property of the US Department of Veterans Affairs.
